# Supplementary material for: Neuronal non-CG methylation is an essential target for MeCP2 function
Source: Mol Cell. 2021 Mar 18;81(6):1260–1275.e12. doi: 10.1016/j.molcel.2021.01.011 (PMC7980222; doi:10.1016/j.molcel.2021.01.011)
Supplement: Document S1. Figures S1–S7 and Tables S1–S4 [file mmc1.pdf]

**Molecular Cell, Volume *81***

## Supplemental information

# Neuronal non-CG methylation is an essential target for MeCP2 function

**Rebekah Tillotson, Justyna Cholewa-Waclaw, Kashyap Chhatbar, John C. Connelly, Sophie A. Kirschner, Shaun Webb, Martha V. Koerner, Jim Selfridge, David A. Kelly, Dina De Sousa, Kyla Brown, Matthew J. Lyst, Skirmantas Kriaucionis, and Adrian Bird**

## SUPPLEMENTAL INFORMATION

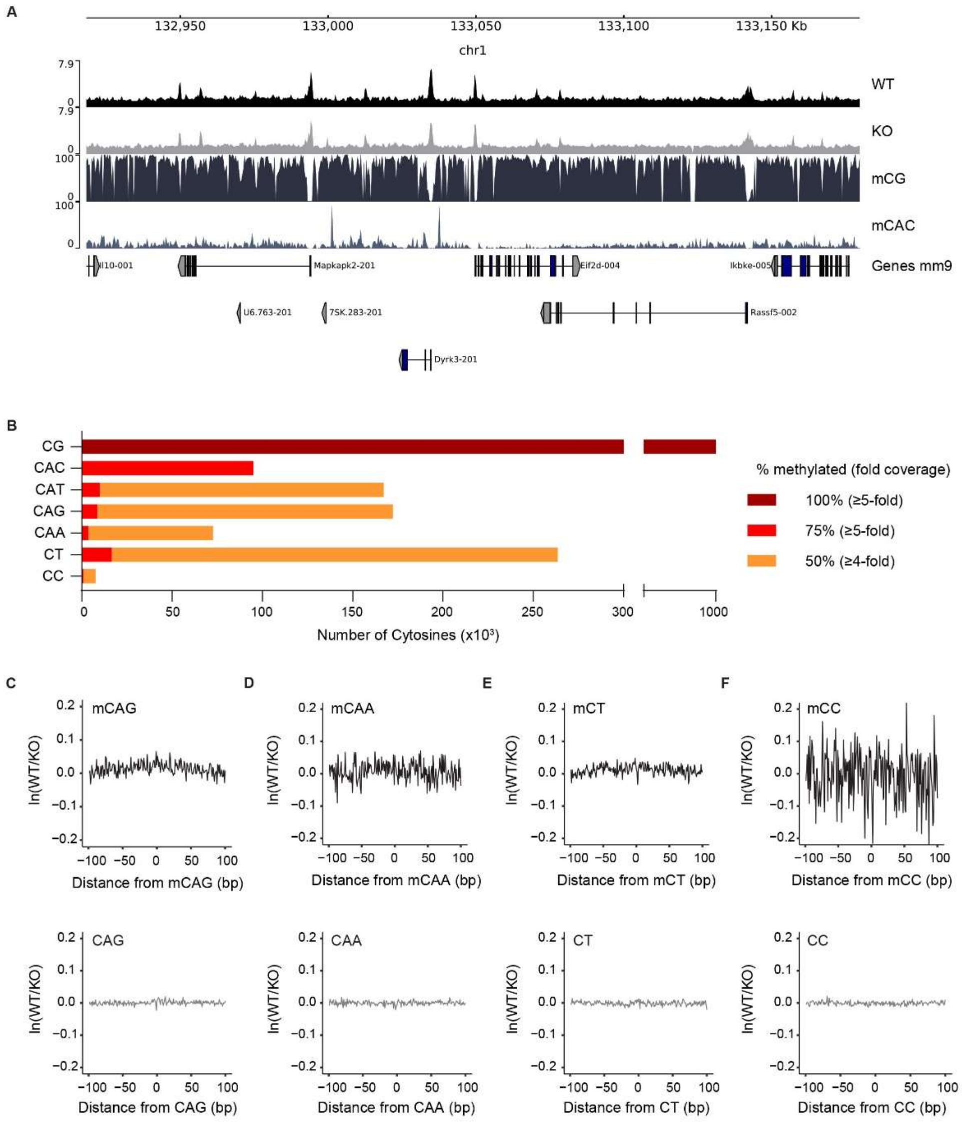


**Figure S1. MeCP2 binds mCG and mCAC and to a lesser extent mCAT.** ***Related to Figure 1***

A. Representative ATAC-seq tracks (cut sites) for WT (black) and KO (grey) shown alongside mCG and mCAC tracks (percentage methylation) from WGBS. Screenshot covers a region of chromosome 1 with several annotated transcriptional units (bottom). Deletion of *Mecp2* does not result in gross changes to chromatin accessibility. B. Bar graph showing the number of highly methylated sites used for ATAC-seq footprinting. mCG sites are 100% methylated on both strands, with a combined ≥5-fold coverage. mCAC sites are ≥75% methylated, with a ≥5-fold coverage. All others are ≥50% methylated, with a ≥4-fold coverage. C-F. ATAC-seq footprints over methylated (upper) and unmethylated (lower) sequences. All CH footprints used 1 million sites with 0% methylation and ≥10-fold coverage.

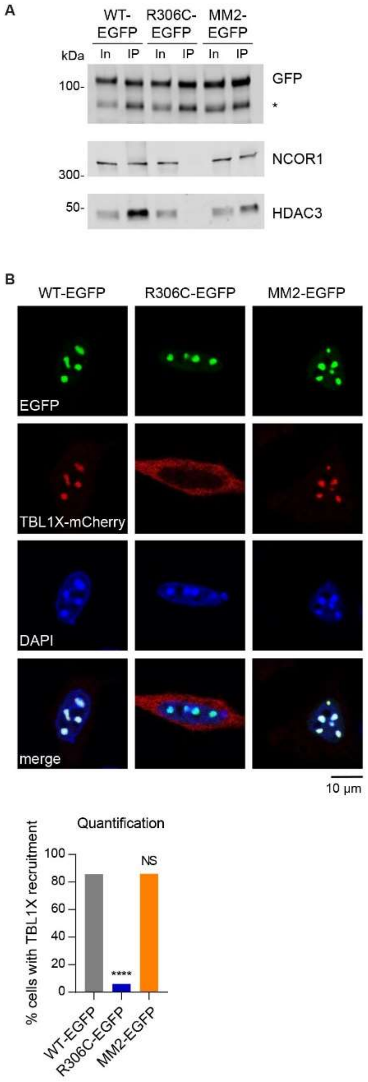


**Figure S2. Chimeric protein MM2 retains the ability to bind to key interaction partners of MeCP2, the NCOR1/2 repressor complexes.** ***Related to Figure 2***

A. EGFP-tagged MM2 immunoprecipitated components of the NCOR1/2 co-repressor complexes: NCOR1 and HDAC3. WT and R306C were used as positive and negative controls for binding, respectively. In = input; IP = immunoprecipitate; * band from degraded protein. B. Representative images showing recruitment of TBL1X–mCherry to heterochromatic foci by EGFP-tagged MM2 when it is co-overexpressed in NIH-3T3 cells. WT and R306C were used as positive and negative controls for TBL1X–mCherry recruitment, respectively. Scale bar, 10 μm. Quantification (below) shows the percentage of cells with focal TBL1X–mCherry localization, evaluated relative to WT-EGFP using Fisher's exact tests: R306C-EGFP, **** $P < 0.0001$; MM2-EGFP, $P > 0.99$. Total numbers of cells counted: WT-EGFP, $n = 156$; R306C-EGFP, $n = 150$; MM2-EGFP, $n = 151$; over three independent transfection experiments.

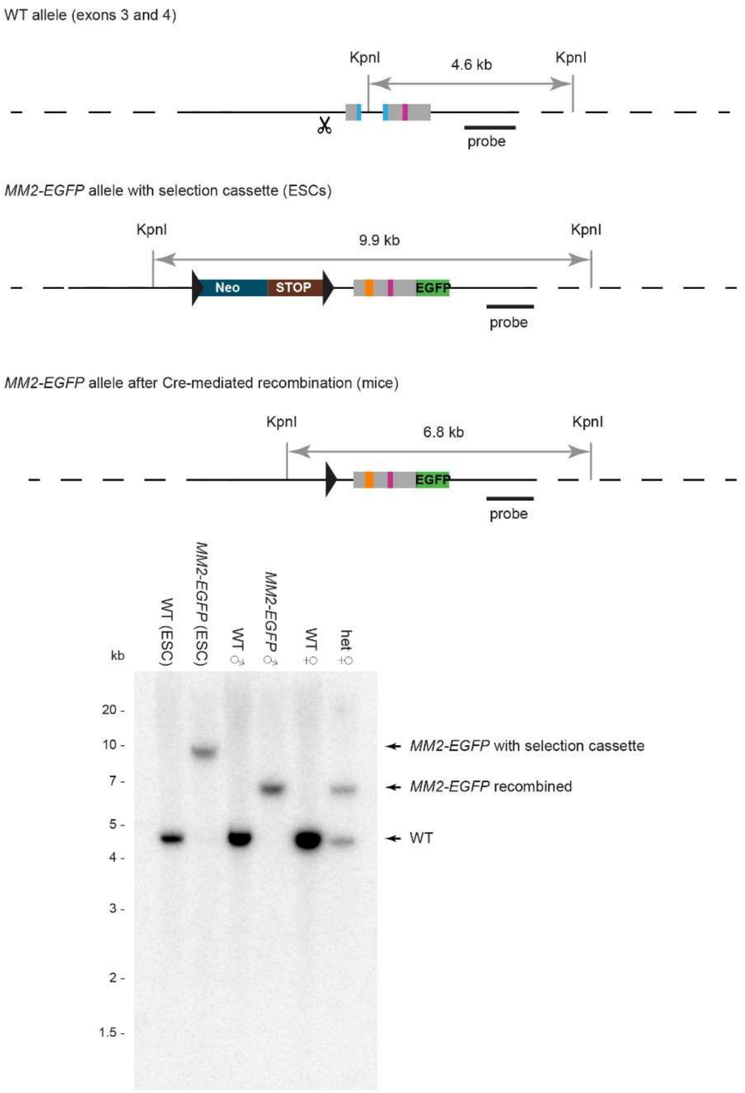


**Figure S3. Generation of *MM2-EGFP* mice, Related to STAR methods: Generation of *MM2-EGFP* mice. *Related to Figure 3***

Diagrammatic representation of *MM2-EGFP* mouseline generation. The endogenous *Mecp2* allele was targeted in male ES cells. The site of Cas9 cleavage in the WT sequence is shown by the scissors symbol. The floxed selection cassette was removed *in vivo* by crossing chimeras with deleter (*CMV-cre*) transgenic mice to produce constitutively expressing *MM2-EGFP* mice. The solid black line represents the sequence encoded in the targeting vector and the dotted lines indicate the flanking regions of mouse genomic DNA. *LoxP* sites are shown as triangles. Key: *Mecp2* MBD = blue, *MM2* MBD = orange, NID = pink, intervening *Mecp2* exonic sequences = grey, EGFP = green, linker = dark green, Neomycin resistance gene = dark blue, transcriptional stop cassette = dark red. Southern blot analysis shows correct targeting of ES cells and successful cassette deletion in the *MM2-EGFP* knock-in mice.

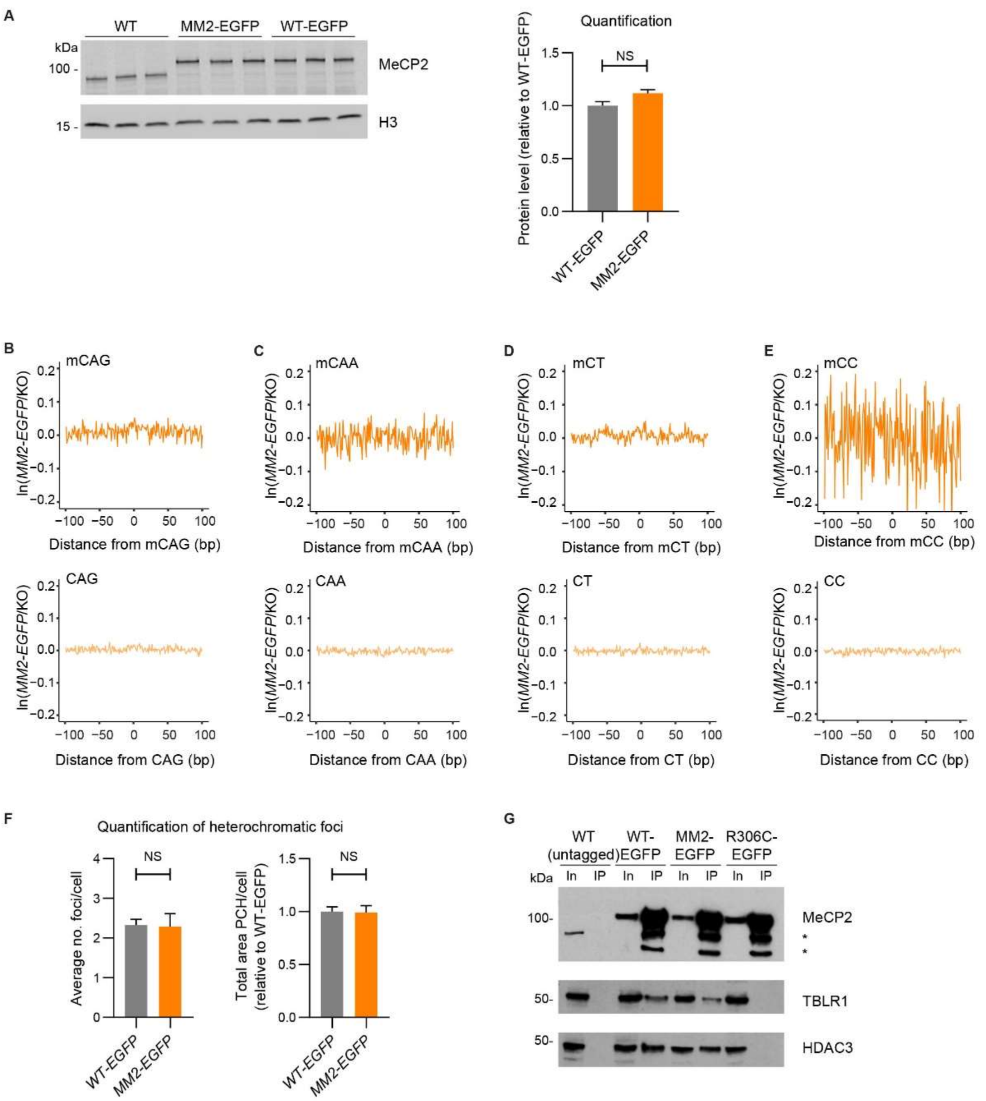


**Figure S4: MM2 binds specifically to mCG *in vivo*. *Related to Figure 3***

A. Western blot analysis of whole-brain extract showing protein sizes and abundance of MM2-EGFP compared to WT and WT-EGFP controls, detected using an N-terminal MeCP2 antibody. Histone H3 was used as a loading control. Quantification (right) of WT-EGFP and MM2-EGFP protein levels, showing mean ± S.E.M. Genotypes were compared using a t-test: NS P = 0.088. B-E. ATAC-seq footprinting of MM2-EGFP at methylated (upper) and unmethylated (lower) CAG (B), CAA (C), CT (D) and CC (E) sites. Equivalent WT ATAC-seq footprinting is shown in Figure S1C-F. F. Quantification of the microscopy analysis shown in Figure 3F. The average number of foci per cell (left) and the total area of pericentromeric heterochromatin per cell (PCH) (right). Total number of cells per genotype: *WT-EGFP* n = 967; *MM2-EGFP* n = 1129, from three biological replicates of each. Graphs show mean ± SEM of biological replicates. Genotypes were compared using t-tests: number of foci NS P = 0.93; total area NS P = 0.94. G. GFP pulldowns from whole brain nuclear extract showing immunoprecipitation of members of the NCOR1/2 co-repressor complexes, TBLR1 and HDAC3, with WT-EGFP and MM2-EGFP. WT (untagged) and R306C were used as negative controls. In = input; IP = immunoprecipitate. * bands from degraded protein.

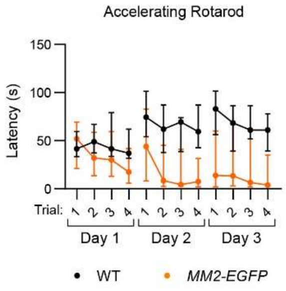


**Figure S5: MM2 knock-in mice display behavioural defects associated with the RTT-like phenotype.** ***Related to Figure 5***

Latency to fall from the accelerating rotarod in four trials was calculated for each of the 3 days of the experiment. Graph shows median and interquartile range between animals. WT animals did not show altered performance over the course of any of the three days: day 1 P = 0.98; day 2 P = 0.39; and day 3 P = 0.08. *MM2-EGFP* animals show fatigue over the course of day 1 * P = 0.022 and day 2 ** P = 0.004, but no change on day 3 P = 0.95. Change in performance was analysed with Friedman tests.

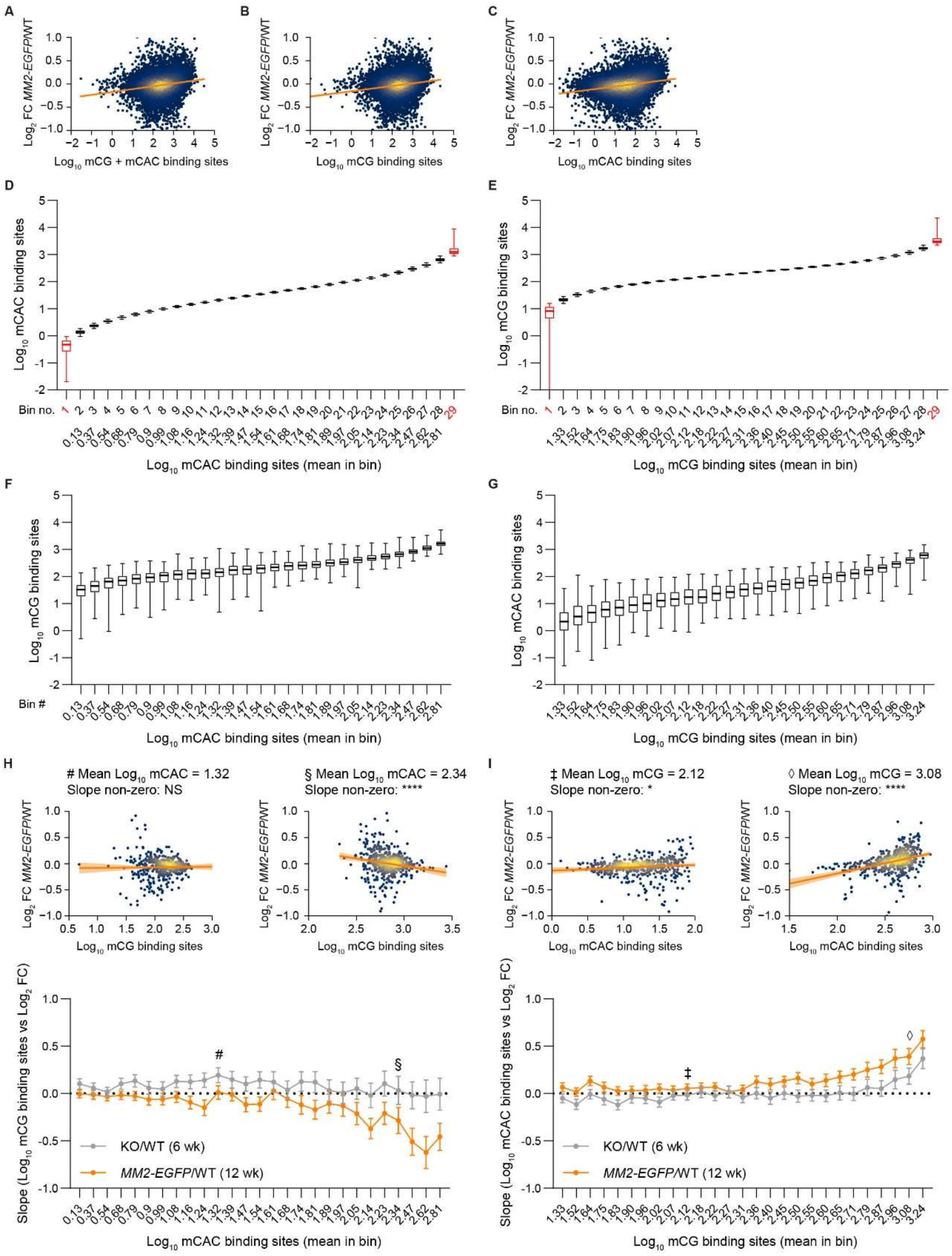


**Figure S6. MM2 represses transcription at mCG but not mCAC sites.** ***Related to Figure 6***

*Legend on next page*

**Figure S6. MM2 represses transcription at mCG but not mCAC sites.** ***Related to Figure 6***

A-C. Correlations between the total number of MeCP2 binding sites per gene (between TSS and TTS): mCG + mCAC (A), mCG (B) and mCAC (C) and transcriptional changes in *MM2-EGFP*/WT hypothalamus tissue at 12 weeks of age. D-G. Genes were binned by the number of mCAC binding sites to determine the effect of mCG on transcription; and by the number of mCG binding sites to determine the effect of mCAC on transcription. Whiskers show minimum-maximum. Bins no. 1 and 29 (shown in red) were excluded due to the high variance in the motif used for binning. D. Number of mCAC binding sites per gene in mCAC bins. E. Number of mCG binding sites per gene in mCG bins. F. Number of mCG binding sites per gene in mCAC bins. G. Number of mCAC sites per gene in mCG bins. H. Correlations between the number of mCG binding sites and transcriptional changes in *MM2-EGFP-EGFP/WT* (12 weeks), in mCAC bins. Two example bins are shown (# Bin 12, mean $\log_{10}$ mCAC = 1.32; and § Bin 25, mean $\log_{10}$ mCAC = 2.34). The slopes of all bins are shown below. I. Correlations between the number of mCAC binding sites and transcriptional changes in *MM2-EGFP-EGFP/WT* (12 weeks), in mCG bins. Two example bins are shown (‡ Bin 11, mean $\log_{10}$ mCG = 2.12; and ◊ Bin 27, mean $\log_{10}$ mCAC = 3.08). The slopes of all bins are shown below. Error on all slopes: 95% CI. See Tables S1-2.

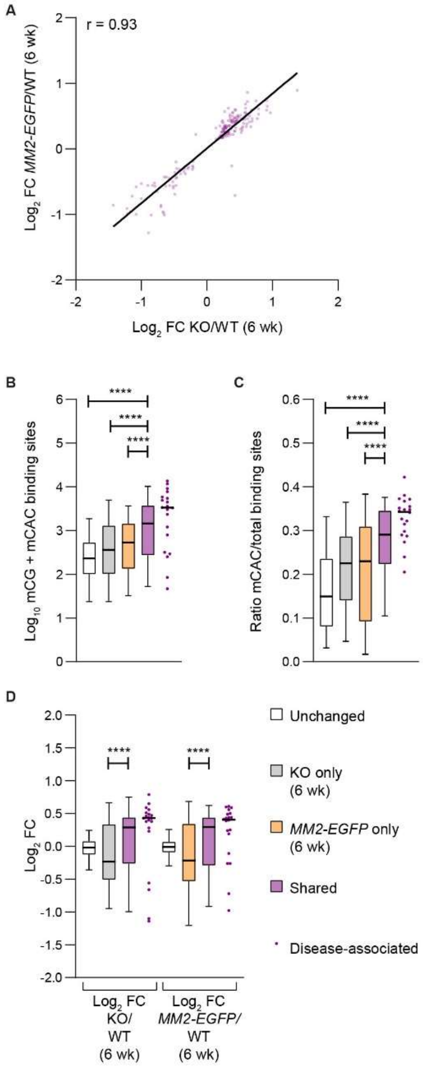


**Figure S7. Shared dysregulated genes are enriched in mCAC and highly upregulated. *Related to Figure 7***

A. Shared genes are dysregulated in the same direction in KO/WT (6 weeks) and *MM2-EGFP*/WT (6 weeks). B. Shared genes have more mCG + mCAC binding sites, compared to genes unchanged in either mutant at 6 weeks and to genes dysregulated in only KO or only *MM2-EGFP* (6 weeks). C. Shared genes have higher mCAC/total binding sites ratios, compared to unchanged genes and to genes dysregulated in only KO or only *MM2-EGFP* (6 weeks). D. Shared genes are more upregulated in both mutants, compared to genes dysregulated in only KO or only *MM2-EGFP* (6 weeks). B-D. Disease-associated genes have exceptionally high mCG + mCAC levels, mCAC/total ratios and are highly upregulated. Median values are indicated by a black bar. Whiskers show 5-95 percentiles. Pairs of gene sets were compared using Mann-Whitney tests: **** P < 0.0001.

**Table S1. Correlations between number of mCG binding sites and transcriptional changes in mCAC bins.** ***Related to Figures 6 and S6***

| Bin no. | Mean mCAC in bin | KO/WT (6 wk) slopes | | | | *MM2-EGFP*/WT (6 wk) slopes | | | | *MM2-EGFP*/WT (12 wk) slopes | | | |
|---|---|---|---|---|---|---|---|---|---|---|---|---|---|
| | | Mean | Min | Max | Non-zero? | Mean | Min | Max | Non-zero? | Mean | Min | Max | Non-zero? |
| 2 | 0.13 | 0.1019 | 0.1565 | 0.04727 | 0.0003 | -0.04461 | -0.000002 | -0.08922 | 0.05 | -0.00426 | 0.03661 | -0.04513 | 0.8378 |
| 3 | 0.37 | 0.05908 | 0.1122 | 0.005918 | 0.0295 | -0.03683 | 0.006171 | -0.07982 | 0.093 | -0.01325 | 0.03468 | -0.06118 | 0.5873 |
| 4 | 0.54 | 0.0173 | 0.06477 | -0.03018 | 0.4744 | -0.09874 | -0.04817 | -0.1493 | 0.0001 | -0.03245 | 0.01221 | -0.0771 | 0.154 |
| 5 | 0.68 | 0.1036 | 0.1612 | 0.04588 | 0.0005 | -0.09853 | -0.04867 | -0.1484 | 0.0001 | -0.01876 | 0.02493 | -0.06245 | 0.3993 |
| 6 | 0.79 | 0.1336 | 0.1981 | 0.06913 | <0.0001 | -0.0094 | 0.04641 | -0.0652 | 0.7409 | -0.02627 | 0.02125 | -0.0738 | 0.278 |
| 7 | 0.9 | 0.05882 | 0.125 | -0.00736 | 0.0814 | -0.03391 | 0.02106 | -0.08889 | 0.2261 | -0.06336 | -0.00488 | -0.1218 | 0.0338 |
| 8 | 0.99 | 0.04805 | 0.1248 | -0.0287 | 0.2193 | -0.04938 | 0.003244 | -0.102 | 0.0658 | -0.06007 | 0.000066 | -0.1202 | 0.0503 |
| 9 | 1.08 | 0.1272 | 0.1909 | 0.06347 | 0.0001 | -0.08529 | -0.02803 | -0.1425 | 0.0036 | -0.03586 | 0.0221 | -0.09382 | 0.2248 |
| 10 | 1.16 | 0.1233 | 0.1961 | 0.05062 | 0.0009 | -0.07127 | 0.002592 | -0.1451 | 0.0586 | -0.09496 | -0.02807 | -0.1618 | 0.0055 |
| 11 | 1.24 | 0.1405 | 0.2175 | 0.06363 | 0.0004 | -0.05294 | 0.01903 | -0.1249 | 0.149 | -0.1497 | -0.06818 | -0.2312 | 0.0003 |
| 12 # | 1.32 | 0.1944 | 0.2732 | 0.1155 | <0.0001 | -0.01177 | 0.05242 | -0.07596 | 0.7188 | 0.01062 | 0.08139 | -0.06015 | 0.7683 |
| 13 | 1.39 | 0.1462 | 0.2347 | 0.05766 | 0.0013 | -0.1526 | -0.08457 | -0.2206 | <0.0001 | -0.01244 | 0.05329 | -0.07816 | 0.7102 |
| 14 | 1.47 | 0.1009 | 0.1778 | 0.02407 | 0.0102 | -0.1109 | -0.04047 | -0.1812 | 0.0021 | -0.1175 | -0.04409 | -0.1908 | 0.0018 |
| 15 | 1.54 | 0.144 | 0.2298 | 0.05826 | 0.001 | -0.1058 | -0.02929 | -0.1823 | 0.0068 | -0.1118 | -0.03542 | -0.1882 | 0.0042 |
| 16 | 1.61 | 0.121 | 0.2295 | 0.01244 | 0.029 | -0.06327 | 0.02889 | -0.1554 | 0.178 | 0.02969 | 0.1231 | -0.06376 | 0.5328 |
| 17 | 1.68 | 0.03768 | -0.05625 | 0.1316 | 0.431 | -0.09179 | -0.01437 | -0.1692 | 0.0202 | -0.05623 | 0.03499 | -0.1474 | 0.2264 |
| 18 | 1.74 | 0.127 | 0.2293 | 0.02469 | 0.0151 | -0.1347 | -0.04546 | -0.2239 | 0.0032 | -0.1194 | -0.02161 | -0.2172 | 0.0168 |
| 19 | 1.81 | 0.1207 | 0.2323 | 0.009107 | 0.0341 | -0.1794 | -0.04696 | -0.3118 | 0.008 | -0.1707 | -0.07098 | -0.2705 | 0.0008 |
| 20 | 1.89 | 0.03366 | 0.1459 | -0.0786 | 0.5561 | -0.09814 | -0.0082 | -0.1881 | 0.0325 | -0.1061 | -0.00581 | -0.2063 | 0.0382 |
| 21 | 1.97 | 0.007044 | 0.1192 | -0.1052 | 0.9019 | -0.131 | -0.032 | -0.2301 | 0.0096 | -0.1311 | -0.02231 | -0.2399 | 0.0183 |
| 22 | 2.05 | 0.05148 | 0.163 | -0.06003 | 0.3648 | -0.1941 | -0.08622 | -0.3019 | 0.0004 | -0.212 | -0.09465 | -0.3294 | 0.0004 |
| 23 | 2.14 | -0.02023 | 0.1098 | -0.1502 | 0.7599 | -0.3301 | -0.2304 | -0.4298 | <0.0001 | -0.3708 | -0.2644 | -0.4773 | <0.0001 |
| 24 | 2.23 | 0.1049 | 0.2365 | -0.02661 | 0.1177 | -0.2197 | -0.0944 | -0.345 | 0.0006 | -0.2083 | -0.09173 | -0.325 | 0.0005 |
| 25 § | 2.34 | 0.0322 | 0.1802 | -0.1158 | 0.6693 | -0.3358 | -0.2159 | -0.4557 | <0.0001 | -0.2851 | -0.1483 | -0.422 | <0.0001 |
| 26 | 2.47 | -0.01775 | 0.1237 | -0.1592 | 0.8053 | -0.4808 | -0.3275 | -0.6341 | <0.0001 | -0.5098 | -0.3681 | -0.6515 | <0.0001 |
| 27 | 2.62 | -0.02933 | 0.1423 | -0.2009 | 0.7371 | -0.5756 | -0.4388 | -0.7125 | <0.0001 | -0.6225 | -0.4547 | -0.7903 | <0.0001 |
| 28 | 2.81 | -0.00665 | 0.1623 | -0.1756 | 0.9384 | -0.4337 | -0.2959 | -0.5715 | <0.0001 | -0.4575 | -0.3142 | -0.6008 | <0.0001 |

Key

Positive correlation, with non-zero slope * P < 0.05, ** P < 0.01, *** P < 0.001, **** P < 0.0001.
Negative correlation, with non-zero slope P < 0.05 > 0.01; (lighter blue), P < 0.01 (darker blue).

**Table S2. Correlations between number of mCAC binding sites and transcriptional changes in mCG bins.** ***Related to Figures 6 and S6***

| Bin no. | Mean mCG in bin | KO/WT (6 wk) slopes | | | | *MM2-EGFP*/WT (6 wk) slopes | | | | *MM2-EGFP*/WT (12 wk) slopes | | | |
|---|---|---|---|---|---|---|---|---|---|---|---|---|---|
| | | Mean | Min | Max | Non-zero? | Mean | Min | Max | Non-zero? | Mean | Min | Max | Non-zero? |
| 2 | 1.33 | -0.04999 | -0.00387 | -0.0961 | 0.0337 | 0.0531 | 0.09544 | 0.01076 | 0.0141 | 0.06635 | 0.1136 | 0.01908 | 0.006 |
| 3 | 1.52 | -0.1146 | -0.06085 | -0.1683 | <0.0001 | 0.03477 | 0.07956 | -0.01002 | 0.1278 | 0.01593 | 0.06003 | -0.02817 | 0.4782 |
| 4 | 1.64 | -0.00743 | 0.04127 | -0.05613 | 0.7646 | 0.1207 | 0.1692 | 0.07229 | <0.0001 | 0.1326 | 0.1817 | 0.08355 | <0.0001 |
| 5 | 1.75 | -0.06007 | -0.00047 | -0.1197 | 0.0482 | 0.1037 | 0.1572 | 0.05017 | 0.0002 | 0.06772 | 0.1205 | 0.01492 | 0.012 |
| 6 | 1.83 | -0.1202 | -0.06815 | -0.1723 | <0.0001 | 0.04979 | 0.08987 | 0.009717 | 0.015 | 0.0267 | 0.07079 | -0.01739 | 0.2346 |
| 7 | 1.90 | -0.04154 | 0.01688 | -0.09995 | 0.163 | 0.04245 | 0.08551 | -0.0006 | 0.0533 | 0.03174 | 0.07704 | -0.01356 | 0.1693 |
| 8 | 1.96 | -0.05237 | -0.00068 | -0.1041 | 0.0471 | 0.04713 | 0.09706 | -0.00279 | 0.0642 | 0.03311 | 0.08658 | -0.02036 | 0.2243 |
| 9 | 2.02 | -0.08982 | -0.03728 | -0.1424 | 0.0008 | 0.04312 | 0.09031 | -0.00406 | 0.0732 | 0.05127 | 0.1009 | 0.001693 | 0.0427 |
| 10 | 2.07 | -0.02131 | 0.02566 | -0.06828 | 0.3732 | 0.08413 | 0.1306 | 0.0377 | 0.0004 | 0.03447 | 0.08002 | -0.01108 | 0.1377 |
| 11 ‡ | 2.12 | -0.01814 | 0.03362 | -0.0699 | 0.4914 | 0.06764 | 0.1123 | 0.02303 | 0.003 | 0.05444 | 0.1016 | 0.007283 | 0.0237 |
| 12 | 2.18 | 0.01455 | 0.07166 | -0.04257 | 0.617 | 0.03627 | 0.0932 | -0.02067 | 0.2113 | 0.0622 | 0.1114 | 0.01295 | 0.0134 |
| 13 | 2.22 | -0.02293 | 0.03024 | -0.0761 | 0.3972 | 0.11 | 0.1547 | 0.06522 | <0.0001 | 0.06964 | 0.1124 | 0.0269 | 0.0015 |
| 14 | 2.27 | 0.01534 | 0.07877 | -0.0481 | 0.635 | 0.07052 | 0.1217 | 0.01934 | 0.007 | 0.01736 | 0.07809 | -0.04337 | 0.5746 |
| 15 | 2.31 | -0.03987 | 0.01721 | -0.09695 | 0.1706 | 0.05309 | 0.1023 | 0.003867 | 0.0346 | 0.04217 | 0.08883 | -0.0045 | 0.0764 |
| 16 | 2.36 | 0.003028 | 0.06179 | -0.05573 | 0.9194 | 0.122 | 0.1724 | 0.0716 | <0.0001 | 0.124 | 0.1851 | 0.06295 | <0.0001 |
| 17 | 2.40 | -0.0467 | 0.01897 | -0.1124 | 0.163 | 0.07351 | 0.1462 | 0.000788 | 0.0476 | 0.09874 | 0.1589 | 0.03856 | 0.0013 |
| 18 | 2.45 | 0.003569 | 0.06852 | -0.06138 | 0.9141 | 0.1414 | 0.1922 | 0.0905 | <0.0001 | 0.1328 | 0.181 | 0.08452 | <0.0001 |
| 19 | 2.50 | -0.0281 | 0.03647 | -0.09268 | 0.3929 | 0.1268 | 0.1862 | 0.06744 | <0.0001 | 0.1624 | 0.2214 | 0.1034 | <0.0001 |
| 20 | 2.55 | -0.01817 | 0.04249 | -0.07884 | 0.5564 | 0.1139 | 0.1686 | 0.05923 | <0.0001 | 0.1011 | 0.1602 | 0.04188 | 0.0009 |
| 21 | 2.60 | -0.02415 | 0.0367 | -0.085 | 0.4359 | 0.1478 | 0.2102 | 0.08536 | <0.0001 | 0.1388 | 0.2053 | 0.07225 | <0.0001 |
| 22 | 2.65 | 0.007451 | 0.0836 | -0.06869 | 0.8476 | 0.1503 | 0.2089 | 0.09167 | <0.0001 | 0.1748 | 0.2355 | 0.1141 | <0.0001 |
| 23 | 2.71 | -0.0032 | 0.06665 | -0.07304 | 0.9284 | 0.2298 | 0.2893 | 0.1703 | <0.0001 | 0.2029 | 0.2622 | 0.1435 | <0.0001 |
| 24 | 2.79 | 0.0633 | 0.1487 | -0.02207 | 0.1458 | 0.2407 | 0.3155 | 0.1659 | <0.0001 | 0.2515 | 0.3294 | 0.1736 | <0.0001 |
| 25 | 2.87 | 0.05235 | 0.1343 | -0.02956 | 0.2098 | 0.2316 | 0.3053 | 0.1578 | <0.0001 | 0.2825 | 0.3549 | 0.2102 | <0.0001 |
| 26 | 2.96 | 0.1464 | 0.2437 | 0.04904 | 0.0033 | 0.3486 | 0.435 | 0.2623 | <0.0001 | 0.3694 | 0.4665 | 0.2722 | <0.0001 |
| 27 ◊ | 3.08 | 0.1834 | 0.2686 | 0.0982 | <0.0001 | 0.3241 | 0.4011 | 0.2472 | <0.0001 | 0.391 | 0.475 | 0.307 | <0.0001 |
| 28 | 3.24 | 0.3674 | 0.471 | 0.2638 | <0.0001 | 0.5107 | 0.598 | 0.4233 | <0.0001 | 0.5743 | 0.6647 | 0.484 | <0.0001 |

Key

Positive correlation, with non-zero slope P < 0.05 > 0.01; (lighter orange), P < 0.01 (darker orange).
Negative correlation, with non-zero slope P < 0.05 > 0.01; (lighter blue), P < 0.01 (darker blue).

**Table S3: Disease Ontology analysis of shared dysregulated genes.** ***Related to Figures 7 and S7***

| DOID | Description | Gene ratio | BgRatio | P value | Adj. P | q value | Genes |
|---|---|---|---|---|---|---|---|
| 0060040 | pervasive developmental disorder | 16/155 | 201/8007 | 1.5E-06 | 0.00056 | 0.0005 | *MEF2C; RBFOX1; AVPR1A; SEMA5A; GRM8; AUTS2; GRIP1; DOCK4; NOS1AP; CNTNAP2; BDNF; OXTR; GRIN2A; NTNG1; TAC1; CNTN4* |
| 0060041 | autism spectrum disorder | 15/155 | 190/8007 | 3.6E-06 | 0.00056 | 0.0005 | *MEF2C; RBFOX1; AVPR1A; SEMA5A; GRM8; AUTS2; GRIP1; DOCK4; NOS1AP; CNTNAP2; BDNF; OXTR; GRIN2A; TAC1; CNTN4* |
| 12849 | autistic disorder | 15/155 | 190/8007 | 3.6E-06 | 0.00056 | 0.0005 | *MEF2C; RBFOX1; AVPR1A; SEMA5A; GRM8; AUTS2; GRIP1; DOCK4; NOS1AP; CNTNAP2; BDNF; OXTR; GRIN2A; TAC1; CNTN4* |
| 0060037 | developmental disorder of mental health | 20/155 | 373/8007 | 3.1E-05 | 0.00361 | 0.0035 | *MEF2C; RBFOX1; AVPR1A; SEMA5A; GRM8; AUTS2; KIRREL3; GRIP1; DOCK4; KCNK9; NOS1AP; CNTNAP2; CCSER1; CA8; BDNF; OXTR; GRIN2A; NTNG1; TAC1; CNTN4* |

Key

DOID: Disease Ontology Unique Identification Number

Gene ratio: Ratio of genes which are part of the disease ontology in the query/input list (subset of the overlap of KO vs WT and MM2 vs WT which are associated with any disease term, not every gene is associated with disease ontology)

BgRatio: Ratio of genes which are part of the disease ontology in the background list (subset of all the genes that are associated with any disease ontology, not every gene is associated with disease ontology)

P-value: p-value associated with the hypergeometric test

Adj. P: Adjusted p-value after performing Benjamini-Hochberg correction

q value/FDR: False Discovery Rate after performing the Benjamini-Hochberg correction

**Table S4: Primer sequences.** ***Related to STAR methods***

| Primer name | Sequence |
|---|---|
| Primers for cloning MBD2[MBD] into pNIC28-Bsa4 | |
| MBD2 F: | TACTTCCAATCCATGAGGGGACCCCGGGCCAC |
| MBD2 R: | TATCCACCTTTACTGTCATCTCTGTTTGTTCTTCTGTAATTTAC |
| Primers for cloning MeCP2[N-term] and MM2[N-term] into pET30b | |
| N-term NdeI F: | GATATACATATGGTAGCTGGGATGTTAGGG |
| N-term His-tag EcoRI R: | AGCTCGAATTCTCAGTGGTGGTGGTGGTGGTGTTCTGATGCTGCTGCCT TTG |
| Primers for site-directed mutagenesis to insert R169G into MM2 | |
| MM2 RG F: | CTGAGCCCTGATTTTCCGATCACTTCCTCCTTC |
| MM2 RG R: | GAAGGAGGAAGTGATCGGAAAATCAGGGCTCAG |
| Primers for amplifying fragments for recombineering MM2 targeting vector | |
| RN F: | AAGCCTCGGCTTCCCCCAAACAGCGGCGCTCCATTATCCGTGACCGGGG AGGCCTGGTGATGATGGCGGGATCG |
| RN R: | GGAGCTTTGGGAGATTTGGGCTTCTTAGGTGGTTTCTGCTCTCTCCTGG ATCAGAAGAACTCGTCAAGAAGGCG |
| TV F: | CCCCAGCAGTGCCAGAA |
| TV R: | CGACCCCTGCCAGTTCC |
| Primers for screening ES cell clones (5' end) | |
| F4: | TCACCATAACCAGCCTGCTCGC |
| R5: | TGCTAAAGCGCATGCTCCAGACT |
| R3: | ATTCGATGACCTCGAGGATCCG |
| Primers for screening ES cell clones (3' end) | |
| F1: | AGTTCTGGAATGGTGAGCAAGG |
| F2: | GACGTAAACGGCCACAAGTTCA |
| R2: | TCAAAAGCAGAGCAGGCAAAAG |
| Primers for sequencing predicted gRNA off-target | |
| Dock5 F: | AGTAAAGTAGATTTGTGGGTTCCG |
| Dock5 R: | GTAGAATGTGGAGATTTCCTCTAGC |
| MM2 genotyping primers | |
| P5: | TGGTAAAGACCCATGTGACCCAAG |
| P7: | GGCTTGCCACATGACAAGAC |
| Cre genotyping primers | |
| Cre F: | GACCGTACACCAAAATTTGCCTGC |
| Cre R: | TTACGTATATCCTGGCAGCGATC |
