# Supplementary material for: Neuronal non-CG methylation is an essential target for MeCP2 function
Source: Mol Cell. 2021 Mar 18;81(6):1260–1275.e12. doi: 10.1016/j.molcel.2021.01.011 (PMC7980222; doi:10.1016/j.molcel.2021.01.011)
Supplement: Document S2. Article plus Supplemental information [file mmc3.pdf]

Molecular Cell

Article

Neuronal non-CG methylation is an essential target for MeCP2 function

Graphical Abstract

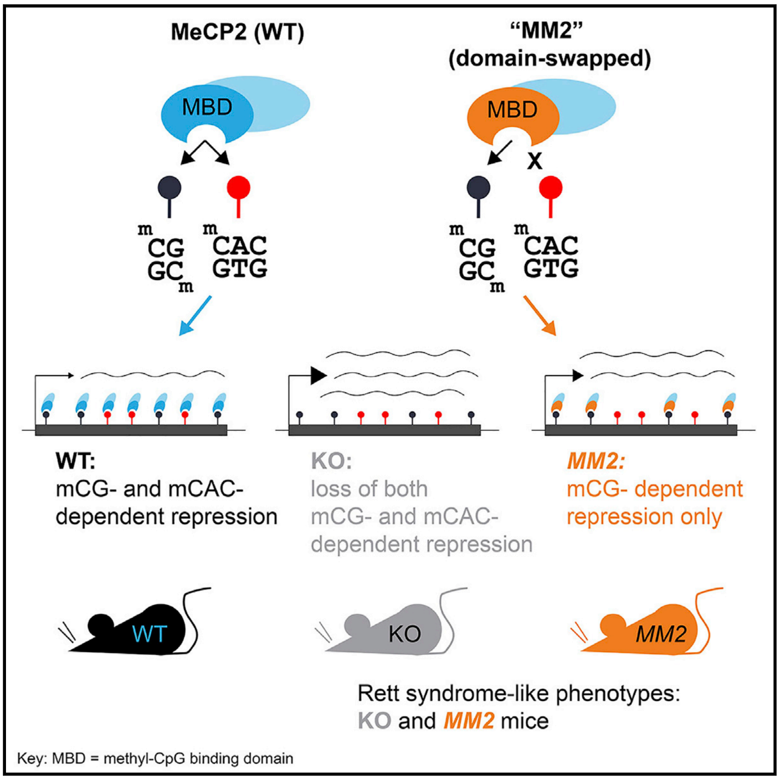

Highlights

- MeCP2 has dual-binding specificity for mCG and mCAC motifs
- Chimeric protein MM2 contains a similar DNA binding domain that only recognizes mCG
- Knockin mice expressing MM2 display Rett-syndrome-like phenotypes
- Genes dysregulated in both *MM2* and *Mecp2* null mice may contribute to Rett syndrome

Authors

Rebekah Tillotson,
Justyna Cholewa-Waclaw,
Kashyap Chhatbar, ..., Matthew J. Lyst,
Skirmantas Kriaucionis, Adrian Bird

Correspondence

a.bird@ed.ac.uk

In Brief

MeCP2 is an epigenetic reader of DNA methylation in two sequence contexts: mCG and mCAC. Tillotson et al. show that mice expressing a chimeric MeCP2 protein that can no longer bind mCAC exhibit severe Rett-syndrome-like phenotypes. The results demonstrate that mCAC binding is an essential property of MeCP2.

Tillotson et al., 2021, Molecular Cell *81*, 1260–1275
March 18, 2021 © 2021 The Authors. Published by Elsevier Inc.
https://doi.org/10.1016/j.molcel.2021.01.011

CellPress

Molecular Cell

Article

Neuronal non-CG methylation is an essential target for MeCP2 function

Rebekah Tillotson,[1,3,4] Justyna Cholewa-Waclaw,[1,3] Kashyap Chhatbar,[1] John C. Connelly,[1] Sophie A. Kirschner,[2] Shaun Webb,[1] Martha V. Koerner,[1] Jim Selfridge,[1] David A. Kelly,[1] Dina De Sousa,[1] Kyla Brown,[1] Matthew J. Lyst,[1] Skirmantas Kriaucionis,[2] and Adrian Bird[1,5,*]
[1]Wellcome Centre for Cell Biology, University of Edinburgh, The Michael Swann Building, King's Buildings, Max Born Crescent, Edinburgh EH9 3BF, UK
[2]Ludwig Institute for Cancer Research, Nuffield Department of Medicine, University of Oxford, Old Road Campus Research Building, Oxford OX3 7DQ, UK
[3]These authors contributed equally
[4]Present address: Genetics and Genome Biology Program, The Hospital for Sick Children, The Peter Gilgan Centre for Research and Learning, Toronto, ON M5G 0A4, Canada
[5]Lead contact
*Correspondence: a.bird@ed.ac.uk
https://doi.org/10.1016/j.molcel.2021.01.011

SUMMARY

DNA methylation is implicated in neuronal biology via the protein MeCP2, the mutation of which causes Rett syndrome. MeCP2 recruits the NCOR1/2 co-repressor complexes to methylated cytosine in the CG dinucleotide, but also to sites of non-CG methylation, which are abundant in neurons. To test the biological significance of the dual-binding specificity of MeCP2, we replaced its DNA binding domain with an orthologous domain from MBD2, which can only bind mCG motifs. Knockin mice expressing the domain-swap protein displayed severe Rett-syndrome-like phenotypes, indicating that normal brain function requires the interaction of MeCP2 with sites of non-CG methylation, specifically mCAC. The results support the notion that the delayed onset of Rett syndrome is due to the simultaneous post-natal accumulation of mCAC and its reader MeCP2. Intriguingly, genes dysregulated in both *Mecp2* null and domain-swap mice are implicated in other neurological disorders, potentially highlighting targets of relevance to the Rett syndrome phenotype.

INTRODUCTION

Heterozygous loss-of-function mutations in the X-linked *MECP2* gene result in Rett syndrome (RTT), a neurological disorder affecting ~1 in 10,000 live female births (Amir et al., 1999). MeCP2 protein was initially identified by its ability to bind methylated CG dinucleotides and shown to repress transcription via the recruitment of histone deacetylase complexes (Lewis et al., 1992; Nan et al., 1997, 1998). Recent evidence indicates that MeCP2 negatively regulates the expression of hundreds of genes via the recruitment of the HDAC3-containing NCOR1/2 co-repressor complexes (Cholewa-Waclaw et al., 2019; Gabel et al., 2015; Lagger et al., 2017; Lyst et al., 2013). In the absence of functional MeCP2, indirect mechanisms also lead to the downregulation of many genes, perhaps connected with a global reduction in total RNA levels (Kinde et al., 2016; Lagger et al., 2017; Li et al., 2013; Yazdani et al., 2012). This multitude of subtle changes to neuronal gene expression is thought to underlie RTT.

In addition to canonical mCG dinucleotides in duplex DNA, MeCP2 targets 5-methylcytosine in a non-CG context (or mCH, where H = A, C, or T). This study focuses on the biological significance of this dual DNA-binding specificity. During early mammalian embryogenesis, the methylation of CG dinucleotides is at first depleted, then re-established, reaching high levels in the bulk genome while absent at unmethylated CpG islands (Bird et al., 1985; Deaton and Bird, 2011). *De novo* CG methylation is laid down by the DNA methyltransferases DNMT3A and DNMT3B and is maintained through cell division by the action of DNMT1 at hemi-methylated sites (Reik, 2007). Unlike plants, which have specific DNA methyltransferases that target CHG and CHH sites (Wendte and Schmitz, 2018), mCH in mammals relies on "off-target" activity by DNMT3A and DNMT3B (Gabel et al., 2015; Guo et al., 2014; Lister et al., 2013; Ramsahoye et al., 2000; Stroud et al., 2017). There appears to be no mechanism to maintain asymmetrical CH methylation, which means that CH methylation is lost in replicating cells (He and Ecker, 2015). Post-mitotic neurons are unique among mammalian somatic cell types in that they accumulate high levels of CH methylation, most prevalently in CAC trinucleotides (Guo et al., 2014; Varley et al., 2013; Xie et al., 2012). This modification arises postnatally due to high levels of DNMT3A, which persist throughout adulthood (Feng et al., 2005; Stroud et al., 2017). The discovery that MeCP2 targets mCAC sites preferentially over all other forms of mCH (Gabel et al., 2015; Lagger et al.,

 © 2021 The Authors. Published by Elsevier Inc.
This is an open access article under the CC BY license (http://creativecommons.org/licenses/by/4.0/).

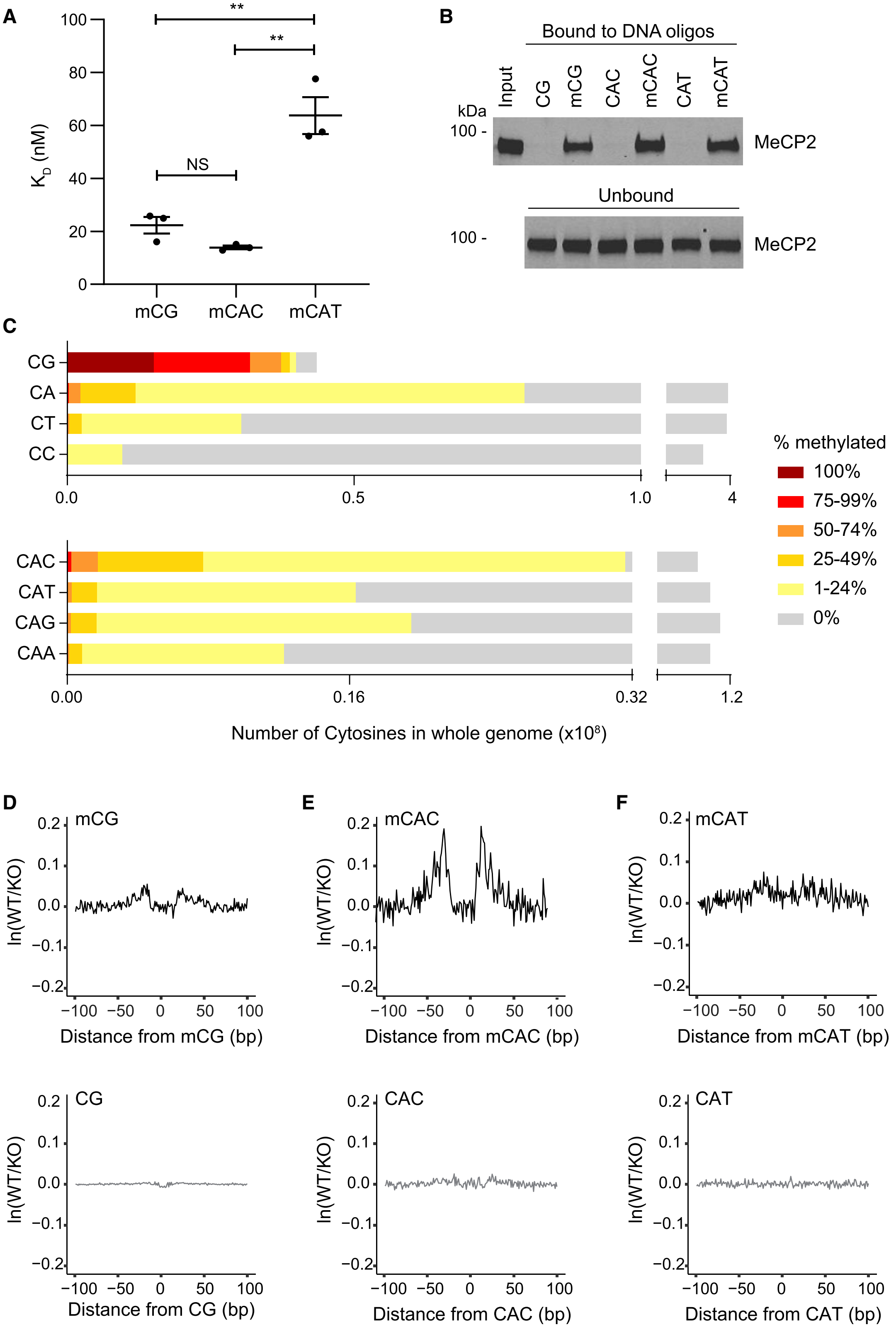

(legend on next page)

2017; Liu et al., 2018; Sperlazza et al., 2017) raised the possibility that this DNA-binding specificity contributes to neuronal function. Non-CG methylation almost doubles the number of available MeCP2 binding sites in neurons (Lagger et al., 2017; Lister et al., 2013; Mo et al., 2015), and the timing of increased CAC methylation coincides with the increase in neuronal MeCP2 protein levels during the first few weeks of life (Guo et al., 2014; Lister et al., 2013; Skene et al., 2010; Stroud et al., 2017).

It is difficult to disentangle the influence of mCG versus mCAC on the regulation of gene expression by MeCP2, as these marks are interspersed with one another (Guo et al., 2014; Lavery et al., 2020). In the present study, we have assessed their relative importance using molecular genetic approaches to separate the effects of mCG and mCAC on MeCP2 function. We directly visualized footprints of MeCP2 bound to mCG and mCAC in native brain chromatin, as well as a subtle footprint over the rarer motif, mCAT (Lagger et al., 2017). To determine the biological importance of the ability of MeCP2 to bind mCAC sites, we took advantage of the fact that the conserved methyl-CpG binding domain (MBD) of the related protein, MBD2, confers binding to mCG sites only. This allowed us to create a chimeric MeCP2-MBD2 (MM2) protein by domain swapping. Despite the retention of mCG binding, knockin mice expressing MM2 developed severe phenotypic features that largely mirrored those seen in mouse models of RTT. We conclude that binding to mCG alone is insufficient for MeCP2 to fulfill its role in the maintenance of neurological function. At a molecular level, the inability to bind mCAC leads to global transcriptional changes in *MM2* mice, with one-third of dysregulated genes also altered in *Mecp2* null mice. Intriguingly, this shared subset is mostly upregulated, highly methylated, and enriched for genes associated with neurological disease, highlighting potentially important contributors to the RTT-like phenotype.

RESULTS

MeCP2 footprints in native brain chromatin

MeCP2 binding to mCG and mCAC sites has been characterized *in vitro* using multiple techniques, and the co-crystal structures of both of these interactions have been solved (Ho et al., 2008; Lagger et al., 2017; Lei et al., 2019; Lewis et al., 1992; Mellén et al., 2012; Sperlazza et al., 2017). A third potential MeCP2 binding motif, mCAT, has been proposed, although this interaction appears to be weaker and is barely detectable in some assays (Lagger et al., 2017; Liu et al., 2018). We used BLI (bio-layer interferometry) to quantify the interactions between the MBD of MeCP2 and DNA probes containing each of three methylated motifs (Figure 1A). MeCP2 binds to both mCG and mCAC, with dissociation constants (K_D) of 22.25 ± 3.12 and 13.90 ± 0.64 nM, respectively. In contrast, binding to mCAT is ~3- to 5-fold weaker, with a K_D of 63.17 ± 6.94 nM. Previously, full-length MeCP2 was shown to bind readily to all three sequences when co-overexpressed with methylated oligonucleotides in HEK293 cells (Lagger et al., 2017). To assess this further, we used beads coated with double-stranded oligonucleotides to pull down endogenous MeCP2 from rat brain lysates. Like mCG and mCAC (Connelly et al., 2020), mCAT-containing DNA efficiently enriched MeCP2 protein in a DNA methylation-dependent manner (Figure 1B). Defining MeCP2 binding sites in native brain chromatin has proven more challenging due to the high abundance of both MeCP2 protein and its short target sequences. Also, the proportion of methylation at individual mCH motifs is, on average, low, in contrast to CGs that are each highly methylated in the bulk genome (Figure 1C). Chromatin immunoprecipitation sequencing (ChIP-seq) analysis gives a relatively featureless signal across the genome, except for a sharp drop at non-methylated CpG islands (Chen et al., 2015; Kinde et al., 2016; Lagger et al., 2017; Skene et al., 2010). Nevertheless, peak-calling algorithms detect enrichment *in vivo* of mCG and mCAC (but not mCAT), coincident with the summits of MeCP2 binding (Cholewa-Waclaw et al., 2019; Gabel et al., 2015; Lagger et al., 2017). To test the specificity of genomic binding more robustly, we adopted the assay for transposase-accessible chromatin using sequencing (ATAC-seq) to reveal MeCP2-specific footprints (Figure S1A). By dividing the ATAC-seq signal from wild-type (WT) samples by the equivalent signal from *Mecp2* null (knockout [KO]) samples, the method has revealed protected genomic regions attributable to MeCP2 at mCG in cultured human neurons (Cholewa-Waclaw et al., 2019). Here, we detect a MeCP2 binding footprint over methylated CG dinucleotides in mouse hypothalamus (Figure 1D). To visualize a MeCP2 binding footprint at mCAC sites with this method, we focused on the subset of sites with >75% methylation (Figures 1E and S1B). The greater prominence of the mCAC footprint compared to the mCG footprint in the hypothalamus does not signify stronger binding to the trinucleotide motif, due to several unquantifiable factors, including site selection criteria and sequence context (e.g., methylated tandem CAC repeats). Footprints were not initially detectable at mCAT, but by lowering stringency to exclude sites <50% methylated (Figure S1B), a subtle footprint at mCAT was observed, but not at other mC-containing motifs (Figures 1F and S1C–S1F). No footprints were detected at unmethylated cytosine in any sequence context (Figures 1D–1F and S1C–S1F). We conclude that the main binding sites for MeCP2 are mCG and mCAC. While binding to mCAT moieties

Figure 1. MeCP2 binds mCG and mCAC, and to a lesser extent, mCAT

(A) Bio-layer interferometry (BLI) analysis of the interaction between the MBD of MeCP2 and methylated DNA probes containing a single mCG, mCAC, or mCAT site. K_D values: mCG 22.25 ± 3.12 nM; mCAC 13.90 ± 0.64 nM; mCAT 63.71 ± 6.94 nM (means ± SEMs). Binding affinity to mCG and mCAC is similar (not significant [NS] p = 0.06); binding to mCAT is significantly weaker than mCG and mCAC (**p = 0.006 and **p = 0.002, respectively), t tests.

(B) Western blot analysis of MeCP2 following pull-down from rat brain nuclear extracts using immobilized DNA containing unmethylated and methylated CG, CAC, and CAT (top) and unbound MeCP2 in the supernatant (bottom).

(C) Whole-genome bisulfite sequencing analysis in the mouse hypothalamus (Lagger et al., 2017) showing the distribution of methylation at each CN dinucleotide (top) and each CAN trinucleotide (bottom). Sites were binned by level of methylation.

(D–F) ATAC-seq footprinting analysis of MeCP2 over methylated (top) and unmethylated (bottom) CG (D), CAC (E), and CAT (F) sites in the mouse hypothalamus.

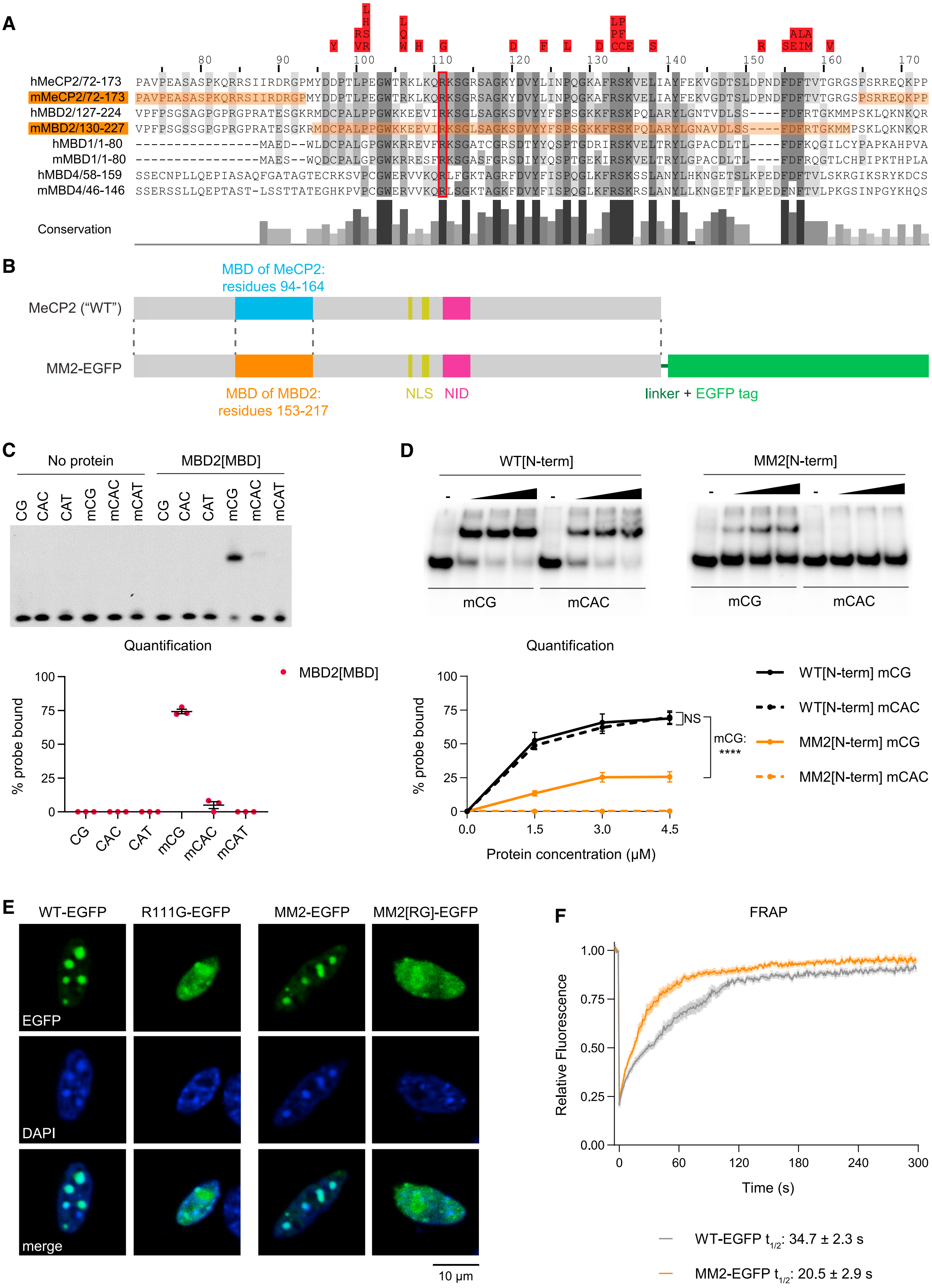

(legend on next page)

is detectable, it is likely to be of less biological significance due to its reduced affinity and lower abundance.

A chimeric MeCP2 that selectively binds mCG

To determine the biological importance of the dual-binding specificity of MeCP2, we sought to modify MeCP2 so that it bound to only one of these motifs. A DNA binding domain that exclusively binds mCAC is unavailable, but domains that only bind mCG are present in other members of the MBD protein family. In particular, the evolutionarily most ancient member of the MBD family, MBD2 (Hendrich and Bird, 1998; Tweedie et al., 1999), is reported to only bind to mCG (Guo et al., 2014; Liu et al., 2018). We substituted the MeCP2 MBD (residues 94–164) with the equivalent domain from MBD2 (residues 153–217) to create a chimeric "MM2" protein (Figures 2A and 2B). After confirming by electrophoretic mobility shift assay (EMSA) analysis that the MBD of MBD2 readily binds to mCG only (Figure 2C), we performed a similar analysis using N-terminal fragments of MM2 and WT MeCP2. As anticipated, MM2 adopted the binding specificity of MBD2, displaying affinity for a DNA probe containing mCG but no affinity for the mCAC probe (Figure 2D).

To test the ability of the chimeric protein to bind mCG sites *in vivo*, we transiently overexpressed the EGFP-tagged MM2 in cultured mouse fibroblasts, in which mCG is highly concentrated in heterochromatic foci (Nan et al., 1996). MM2-EGFP colocalized efficiently with pericentromeric heterochromatin in a manner indistinguishable from WT-EGFP (Figure 2E). To confirm that mCG binding is mediated by the introduced MBD, we showed that mutation of the essential arginine equivalent to R111 of MeCP2 (see Figure 2A) to glycine (Kudo et al., 2003) disrupts the localization of MeCP2 (Figure 2E). To assess the binding dynamics between MM2-EGFP and heterochromatin, we performed transient transfection followed by fluorescence recovery after photobleaching (FRAP). WT-EGFP binding recovered with a half-life of 34.7 ± 2.3 s (Figure 2F), consistent with previous studies (Ghosh et al., 2010; Klose et al., 2005; Ludwig et al., 2017). The half-life of MM2-EGFP was 20.5 ± 2.9 s, indicating that the chimeric protein exchanges slightly faster ($p < 0.0001$, Mann-Whitney test). This may reflect a somewhat reduced binding affinity for mCG, as also suggested by the lower fraction of probe bound in the EMSA assay (Figure 2D). Notably, this difference is much less severe than that of RTT-causing mutations within the MBD domain (Ballestar et al., 2000; Schmiedeberg et al., 2009).

The key function of MeCP2 is to form a bridge between methylated DNA and the NCOR1/2 co-repressor complexes dependent on the MBD and NID (NCOR1/2 interaction domain), respectively (Guy et al., 2018; Kruusvee et al., 2017; Lyst et al., 2013; Tillotson et al., 2017). MM2 retains the NID (residues 298–309) and, as expected, was able to pull down NCOR1/2 complex subunits HDAC3 and NCOR1 when transiently overexpressed in HeLa cells (Figure S2A). Likewise, MM2 retains the ability to recruit TBL1X (its direct binding partner in the NCOR1/2 complexes) to mCG-rich heterochromatic foci when co-expressed in cultured fibroblasts (Figure S2B). The NID-abolishing RTT mutation R306C served as a negative control in both of these assays (Lyst et al., 2013). Overall, the behavior of MM2 in all *in vitro* and cell culture-based assays supports the notion that this chimeric protein retains the essential properties of MeCP2, with the major exception that its DNA binding is restricted to mCG sites.

MM2 selectively binds mCG *in vivo* in knockin mice

To determine the function of MM2 *in vivo*, we produced knockin mice by replacing the endogenous *Mecp2* allele with DNA sequence encoding *MM2* fused to a C-terminal EGFP tag (Figure S3). MM2-EGFP levels in whole brain are slightly higher than WT-EGFP and untagged WT protein by both western blotting and flow cytometry (~116% of WT-EGFP; Figures 3A and S4A). To determine the binding specificity of MM2-EGFP *in vivo*, we used ATAC-seq footprinting of hypothalamus (see Figures 1D–1F) by dividing the ATAC-seq profiles for MM2-EGFP by the equivalent *Mecp2* null (KO) data. A footprint was detected at mCG sites but not at mCAC or mCAT sites (Figures 3B–3D). There was also no MM2-EGFP binding at methylated motifs that MeCP2 cannot recognize (mCAG, mCT, or mCC) or at unmethylated motifs (Figures 3B–3D and S4B–S4F). Therefore, the binding specificity of the chimeric protein is restricted to mCG sites *in vivo*, consistent with the *in vitro* data (Figure 2D). Compared to WT protein, MM2-EGFP had a lower affinity for mCG in EMSA and FRAP (Figures 2D and 2F), yet the similar

Figure 2. Chimeric protein MM2 has the DNA binding properties of MBD2 in the context of MeCP2

(A) Alignment of human (h) and mouse (m) MeCP2, MBD2, MBD1, and MBD4, shaded by percentage identity, with conservation below. Annotations: RTT-causing missense mutations within the MBD (red, above); conserved arginine at position 111 in MeCP2 (boxed in red); sequences from mMeCP2 and mMBD2 used in MM2 (shaded in orange).

(B) Schematic showing the design of the chimeric protein MM2, where the MBD of MBD2 (residues 153–217) was inserted into the MeCP2 protein sequence to replace the endogenous MBD (residues 94–164). MM2 was tagged at the C terminus with EGFP.

(C) EMSA analysis of the MBD of human MBD2 (2 μM) binding to DNA probes containing a single unmethylated or methylated CG, CAC, or CAT site. Quantification is shown below (means ± SEMs).

(D) EMSA analysis of N-terminal fragments (residues 1–205) of wild-type (WT) MeCP2 and MM2 using DNA probes containing a single mCG or mCAC site (protein concentrations 0–4.5 μM). Quantification is shown below (means ± SEMs). Binding affinity was compared using 2-way ANOVA: WT mCG versus mCAC, NS $p = 0.613$; mCG WT versus MM2, ****$p < 0.0001$.

(E) Representative images showing localization of EGFP-tagged MeCP2 (WT) and MM2 proteins to mCG-rich heterochromatic foci following transient overexpression in mouse fibroblasts. Mutation of the MBD (R111G and the equivalent mutation in MM2) abolished binding, resulting in diffuse localization. Scale bar, 10 μm.

(F) FRAP analysis at heterochromatic foci. Total numbers of cells analyzed: WT-EGFP, n = 27; MM2-EGFP, n = 28 (3 independent transfections). Graph shows fluorescence relative to prebleach, means ± SEMs. Half-lives: WT-EGFP = 34.66 ± 2.32 s; MM2-EGFP = 20.46 ± 2.88 s (means ± SEMs). Half-lives were compared using a Mann-Whitney test: ****$p < 0.0001$.

CellPress
OPEN ACCESS

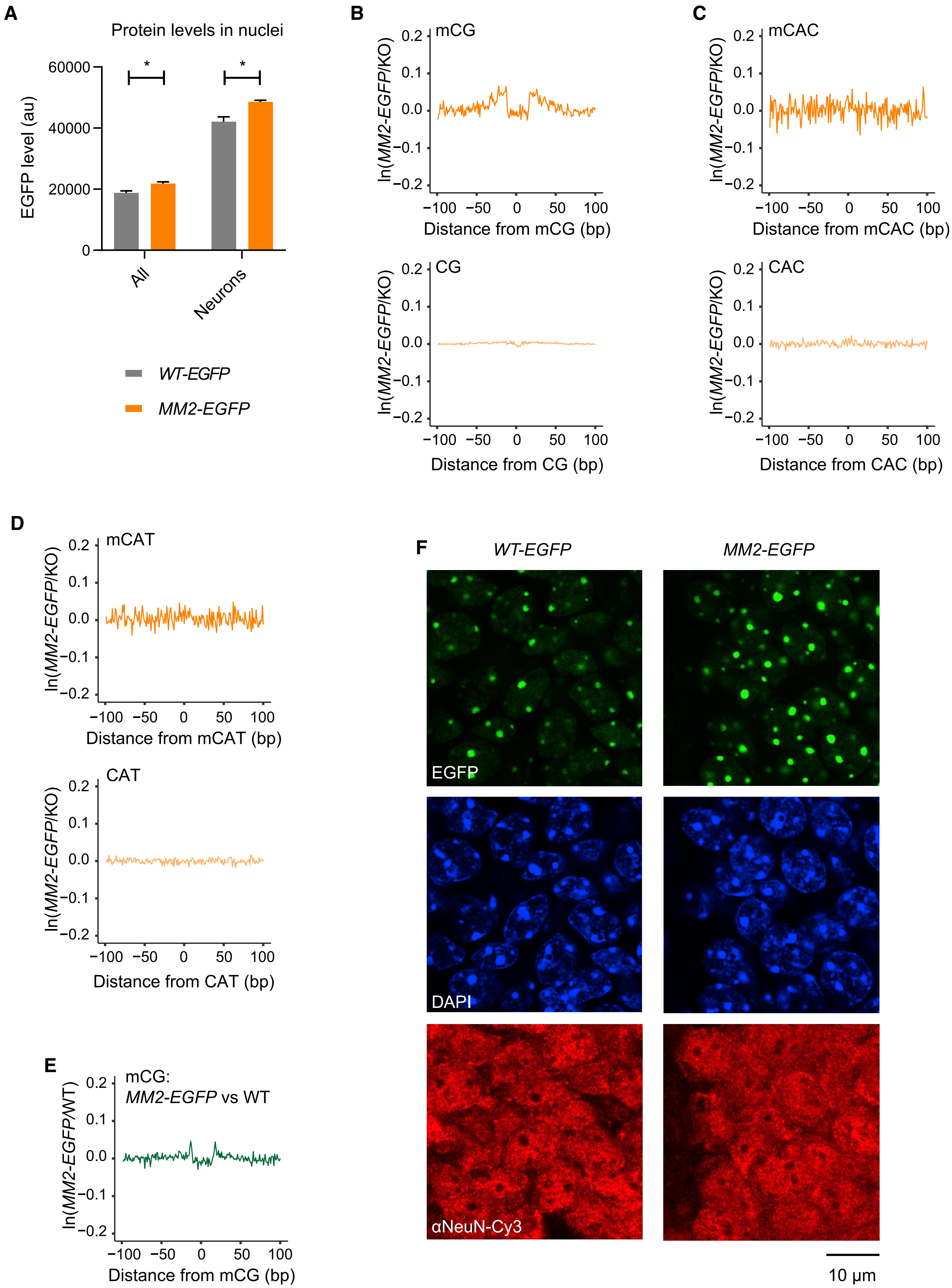

(legend on next page)

appearance of ATAC-seq footprints (Figures 1D and 3B) suggested similar occupancy of these sites *in vivo*. To address this further, we normalized the *MM2-EGFP* ATAC-seq profile to WT instead of KO profiles. As expected, if occupancy is approximately equivalent, this almost canceled out the signals, leaving a barely discernible footprint at mCG. The weak residual MM2-EGFP/WT footprint implies that MM2-EGFP is slightly more enriched than WT at mCG motifs, perhaps due to the availability of extra MM2-EGFP molecules that would otherwise be sequestered at mCAC sites. Efficient binding of MM2-EGFP to mCG *in vivo* was further evidenced by its colocalization to heterochromatic foci in the brains of the knockin mice (Figure 3F). This did not appear to affect the structure of heterochromatin as the number and size of DAPI foci was unchanged (Figure S4F). In addition to its DNA binding properties, we used IP to confirm that MM2-EGFP retains the ability to bind members of the NCOR1/2 co-repressor complex in brain tissue (Figure S4G). These results show that the MBD domain swap has uncoupled the DNA binding specificities of MeCP2, abolishing its interaction with sites of non-CG methylation in the brain.

MM2 knockin mice display an RTT-like phenotype

To determine the phenotypic consequences of the domain swap, we assessed weekly a cohort of hemizygous male *MM2-EGFP* mice and WT littermate controls (n = 10 per genotype) for overt symptoms associated with mouse models of RTT: hypoactivity, abnormal gait, hind-limb clasping, tremor, irregular breathing, and deterioration of general condition (Guy et al., 2007). Like mice with RTT-causing mutations, *MM2-EGFP* mice were born healthy and the onset of symptoms occurred shortly after weaning (Figure 4A). The phenotypes became progressively more severe until animals reached their humane endpoint, with a median survival of 29.5 weeks (Figure 4B). *MM2-EGFP* mice weighed less than WT littermates throughout their lifetime (Figure 4C), consistent with RTT models on a C57BL/6J background (Brown et al., 2016; Guy et al., 2001). In addition, acute loss of body weight, which accompanies severe disease in RTT models, was correlated with morbidity in the *MM2-EGFP* mice. Mouse models of RTT vary in severity depending on MeCP2 mutation, mirroring trends observed in human patients (Brown et al., 2016; Cuddapah et al., 2014). *MM2-EGFP* mice lie in the middle of the RTT severity spectrum, closely following the progression seen in *R306C-EGFP* mutant mice (Figures 4D–4F). More detailed analysis of the six overt symptoms that were scored weekly revealed that with the exception of tremors, *MM2-EGFP* mice recapitulated the RTT phenotypic signature (Figure 4G). This was most obvious when phenotypic severity scores of *MM2-EGFP*, *R306C-EGFP*, and *R133C-EGFP* mice were aligned to focus on the 5 weeks before reaching their humane endpoint (Figure 4H).

A second cohort of *MM2-EGFP* male mice and WT littermates (n = 10 per genotype) underwent a series of behavioral tests at 10–11 weeks of age. Like RTT models (Brown et al., 2016), *MM2-EGFP* mice displayed decreased anxiety in the elevated plus maze, spending significantly more time in the open arms than WT littermate controls (Figure 5A). *MM2* mice were slightly hyperactive in the open field test (Figure 5B), which differs from the reduced activity characteristic of RTT models. However, both *MM2-EGFP* and RTT models displayed progressive loss of spontaneous activity as phenotypes worsened (Figures 4G and 4H). *MM2-EGFP* mice exhibited a trend toward reduced motor coordination in the hanging wire test (Figure 5C). Motor function was better assessed by the accelerating rotarod, in which WT mice showed an increasing ability to stay on the rotarod over the 3 days, but the *MM2-EGFP* mice had significantly impaired performance compared to the controls on days 2 and 3 (Figure 5D). The poor performance of the *MM2-EGFP* mice is due to exercise fatigue (Figure S5), in addition to lack of motor learning. Motor deficits are observed in all mouse models of RTT (Brown et al., 2016; Goffin et al., 2011; Tillotson and Bird, 2019). In summary, *MM2-EGFP* mice share most of the phenotypic features found in mice that are deficient in functional MeCP2, including delayed symptom onset, hind limb clasping, motor defects, decreased anxiety, and premature death.

MM2 represses transcription at mCG but not mCAC sites

Global transcriptional changes caused by the deficiency of functional MeCP2 are thought to underlie RTT (Chahrour et al., 2008; Chen et al., 2015; Gabel et al., 2015; Kinde et al., 2016; Lagger et al., 2017; Renthal et al., 2018). However, the relative contribution of mCG and mCAC to MeCP2-dependent transcriptional regulation is not known. To address this, we performed RNA-seq using hypothalamus isolated from *Mecp2* null (KO) and WT littermate controls at 6 weeks of age, when KO mice are symptomatic but have not reached their humane endpoint. For comparison, we harvested tissue from *MM2-EGFP* mice and WT littermate controls at 2 time points: 6 weeks (age matched with KO) and 12 weeks (when the phenotypes of the 2 mutants are approximately similar). As shown previously (Kinde et al., 2016), gene expression changes in KO compared to WT controls are positively correlated with the total number of MeCP2 binding sites (mCG + mCAC) in gene bodies (Figure 6A). Plotting these transcriptional changes against each motif separately appears to

Figure 3. MM2 binds mCG but not mCAC or mCAT *in vivo*

(A) Flow cytometry analysis of protein levels in nuclei from whole brain (All) and the high-NeuN subpopulation (Neurons) in WT-EGFP (n = 3) and MM2-EGFP (n = 3), detected using EGFP fluorescence. Graph shows means ± SEMs, and genotypes were compared by t tests. All: MM2-EGFP levels are 116% ± 0.03% of WT-EGFP, *p = 0.030; Neurons: 116% ± 0.01% of WT-EGFP, *p = 0.018; au, arbitrary units.

(B–D) ATAC-seq footprinting analysis of MM2-EGFP over methylated (top) and unmethylated (bottom) CG (B), CAC (C), and CAT (D) sites in the mouse hypothalamus. Equivalent WT ATAC-seq footprinting is shown in Figures 1D–1F.

(E) Direct comparison of ATAC-seq profiles over mCG sites between *MM2-EGFP* and WT samples.

(F) WT-EGFP and MM2-EGFP are both localized at mCG-rich pericentromeric heterochromatin in the brains of knockin mice (representative images of the dentate gyrus of the hippocampus). WT-EGFP and MM2-EGFP were visualized by EGFP fluorescence, heterochromatic foci appear as DAPI bright spots, and NeuN staining shows neuronal nuclei. Scale bar, 10 μm.

CellPress
OPEN ACCESS

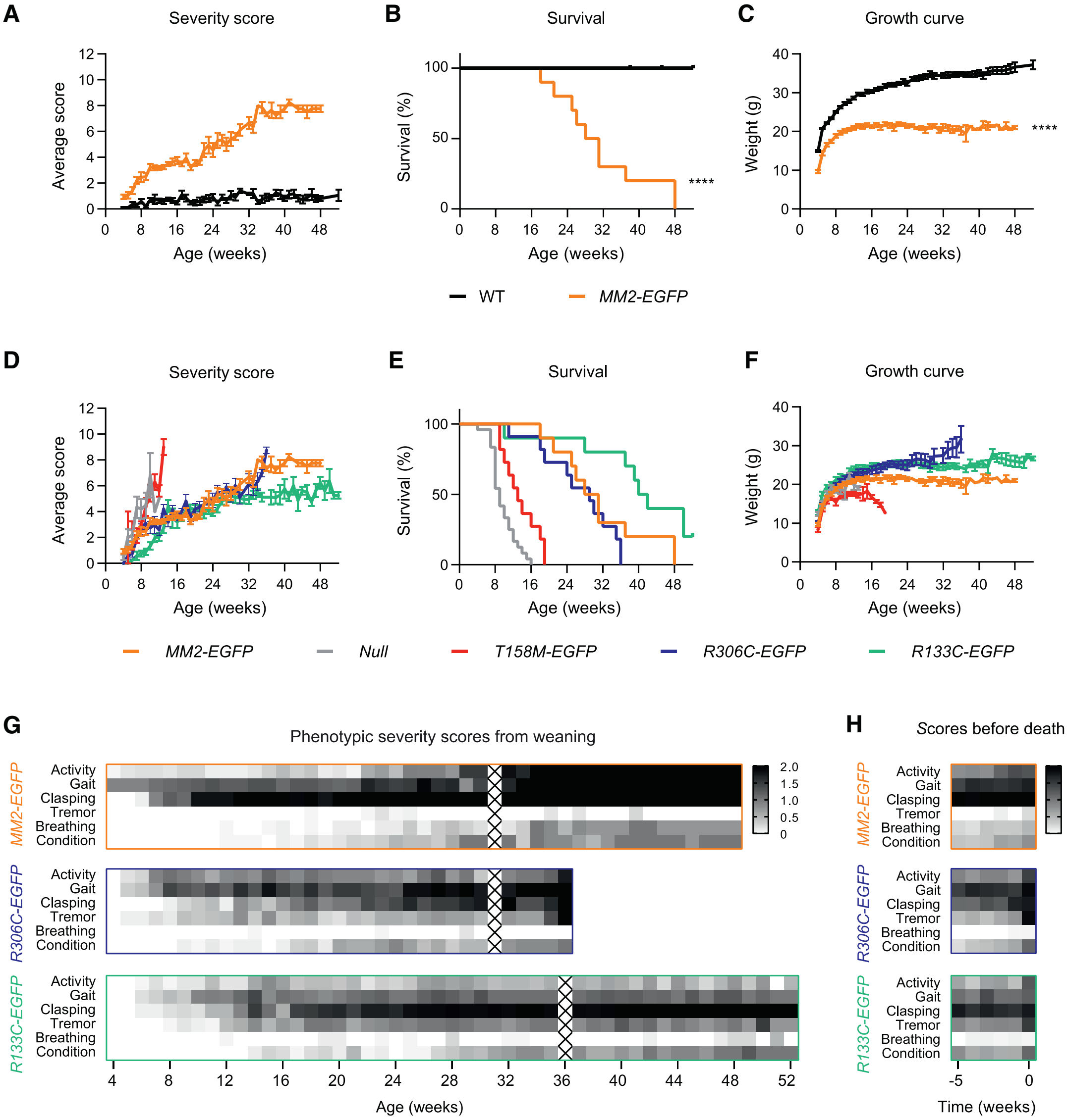

Figure 4. *MM2* knockin mice display overt RTT-like neurological defects

(A) Phenotypic severity scores of hemizygous male *MM2-EGFP* mice (n = 10) compared with their WT littermates (n = 10) over 1 year. Graph shows mean scores ± SEMs.

(B) Kaplan-Meier plot of survival of the scoring cohort. Two WT controls were culled due to injuries, aged 38 and 45 weeks (censored). Genotypes were compared using a Mantel-Cox test: ****$p < 0.0001$.

(C) Growth curve of the scoring cohort. Graph shows mean values ± SEMs. Genotypes were compared using mixed-effects analysis: ****$p < 0.0001$.

(D–F) Phenotypic severity score (D), survival (E), and growth curves (F) of *MM2-EGFP* mice over 1 year compared to *Mecp2* null mice (n = 12/24/20) and models carrying patient mutations: *T158M-EGFP* (n = 7/11/15), *R306C-EGFP* (n = 11), and *R133C-EGFP* (n = 10) (Brown et al., 2016).

(G and H) Heatmaps of the phenotypic scores of *MM2-EGFP*, *R306C-EGFP*, and *R133C-EGFP* mice shown in (D), divided into the 6 categories. Plots are shaded according to the mean score for each category. Scores were analyzed according to age (G) and for the 5 weeks before each mouse reaches its humane endpoint (H). Weeks in which mice were not assessed are marked with an X.

show the loss of both mCG- and mCAC-dependent repression in KO mice, as expected (Figures 6B and 6C), but the strong correlation between the number of mCG and mCAC sites in genes (Figure 6D; r = 0.90) means that this analysis with respect to one motif will also be substantially influenced by the other motif. This could account for the positive trends observed between *MM2-EGFP*/WT transcriptional changes and both mCG and mCAC abundance (Figures 6E–6G and S6A–S6C), despite the predicted loss of only mCAC-dependent repression in *MM2-EGFP* mice.

To distinguish the effects of the two motifs, we binned genes according to the number of mCAC sites per transcription unit to determine the effect of mCG (Figure S6D). In all of the bins except the first and last, the low variation in mCAC level but

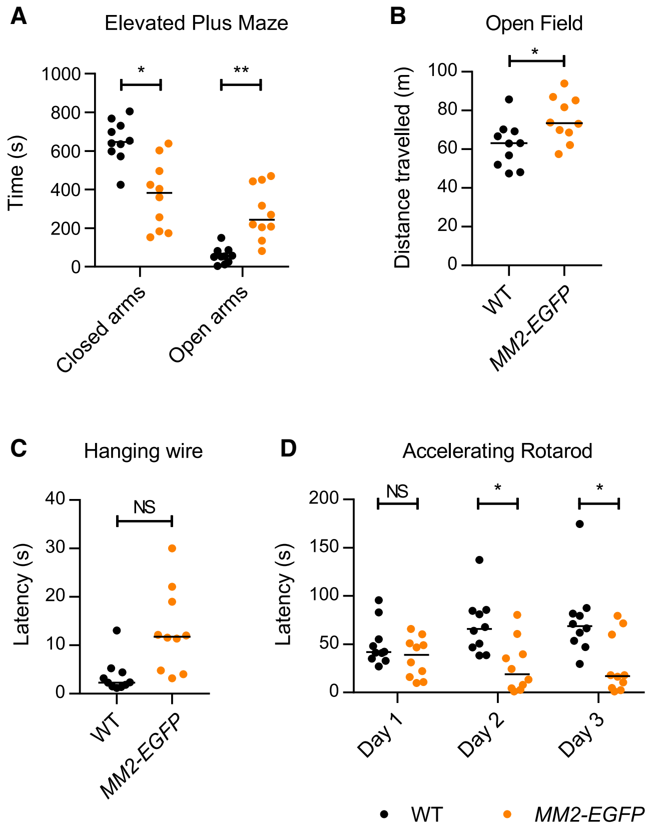

Figure 5. MM2 knockin mice display behavioral defects associated with the RTT-like phenotype

(A–D) Behavioral analysis of a separate cohort performed at 10–11 weeks of age: *MM2-EGFP* (n = 10), compared with their WT littermates (n = 10). Graphs show individual values and medians, and statistical significance as follows: NS $p > 0.05$, *$p < 0.05$, **$p < 0.01$.

(A) Time spent in the closed and open arms of the elevated plus maze. Genotypes were compared using Kolmogorov-Smirnov (KS) tests: closed arms, *$p = 0.015$; open arms, **$p = 0.003$.

(B) Distance traveled in open field test. Genotypes were compared using a t test: *$p = 0.022$.

(C) Mean time taken for animals to bring a hind paw to the wire in the hanging wire test in 3 trials (each up to 30 s). Genotypes were compared using a KS test: $p = 0.055$.

(D) Mean latency to fall from the accelerating rotarod in 4 trials was calculated for each of the 3 days of the experiment. Genotypes were compared using KS tests: day 1, $p = 0.401$; day 2, *$p = 0.015$; and day 3, *$p = 0.015$. WT animals displayed significant improvement over the 3 days (*$p = 0.026$); *MM2-EGFP* animals did not change significantly ($p = 0.314$); analyzed with Friedman tests.

high variation in mCG across the constituent genes enabled the assessment of mCG-dependent repression. We therefore used bins nos. 2–28 for this analysis. We similarly binned by mCG number to assess mCAC-dependent repression (Figures S6D–S6G). Methylation levels in each bin were plotted against $\log_2$-fold changes in KO/WT or *MM2-EGFP*/WT to give a line whose slope reflects the effect of mCG or mCAC on transcription (Figures 6H and 6I). Applying this first to KO mouse hypothalamus, we observed the loss of mCG-dependent repression (slopes above zero) compared to WT controls (Figure 6H; Table S1) in bins with lower levels of methylation, exemplified by the bin with an average of $\log_{10}$ 1.32 mCAC motifs (denoted by #). Genes with higher mCAC levels showed no evidence of repression (example bin denoted by §), indicating that mCG-dependent repression is minimal where mCAC is relatively abundant. In contrast, mCAC-dependent repression was restricted to bins with higher mCG (Figure 6I; Table S2), as exemplified by the bin with an average of $\log_{10}$ 3.08 mCG motifs per gene (denoted by ‡), whereas at lower mCG levels, mCAC-dependent repression was not apparent (example bin denoted by ‡). In line with this interpretation, the proportion of all binding sites that are mCG is higher in bins showing mCG-dependent repression (Figure 6J), while the proportion of mCAC binding sites is highest in bins showing mCAC-dependent repression (Figure 6K).

Having separated the effects of mCG and mCAC in the complete absence of MeCP2, we next performed this analysis on transcriptional changes in *MM2-EGFP* mice compared to 6-week-old WT controls. The profile of mCAC-dependent transcriptional deregulation closely tracked that of KO/WT profiles across mCG bins, consistent with the absence of mCAC-mediated repression in both mutants (Figure 6I; Table S2). However, the profile of mCG-dependent deregulation differed from that seen in KO/WT. As expected, if mCG-dependent repression is preserved in *MM2-EGFP* mice, the positive slope observed for KO/WT in bins with lower levels of mCAC was lost in *MM2-EGFP*/WT (slopes of ~0), (Figure 6H, example bin #; Table S1). We noted that in bins with higher mCAC methylation, transcriptional changes were negatively correlated with the number of mCG binding sites, suggesting somewhat increased mCG-dependent repression of these genes in *MM2-EGFP* mice compared to WT controls. As WT MeCP2 exerts little inhibitory effect at mCG sites in these high-mCAC genes, this effect may be due to the slight increase in MM2-EGFP abundance compared to native protein (Figures 3A and S4A). Also, the inability of MM2-EGFP to target mCAC may make it more available for mCG-mediated repression. This effect is not sufficient to prevent the upregulation of the highly methylated genes, however, as the primary driver of their repression is normally mCAC, which MM2-EGFP does not recognize. These results were replicated in the hypothalamus tissue harvested at 12 weeks (Figures S6H and S6I). In summary, the evidence demonstrates that MM2-EGFP protein has specifically lost the ability to repress the transcription at mCAC sites, disproportionally affecting genes with higher levels of methylation. This implicates loss of mCAC-dependent repression as the primary cause of the severe RTT-like phenotypes that we observe in *MM2-EGFP* mice.

Shared dysregulated genes are implicated in disease

RTT may be caused by the aggregate effect of small transcriptional changes at hundreds of genes. Alternatively, a few dysregulated genes may be primarily responsible for the phenotypes, the remainder being relatively neutral. Given the remarkable similarity between the phenotypes of *MM2-EGFP* and RTT mice, these genes are expected to be affected in both mutants. Significantly changed genes overlapped by approximately one-third in both the age-matched and symptom-matched datasets (Figure 7A, purple shading). Importantly, for almost all of the 316 shared genes, the direction of transcriptional change was the same in KO and *MM2-EGFP* mice (Figures 7B and S7A). To

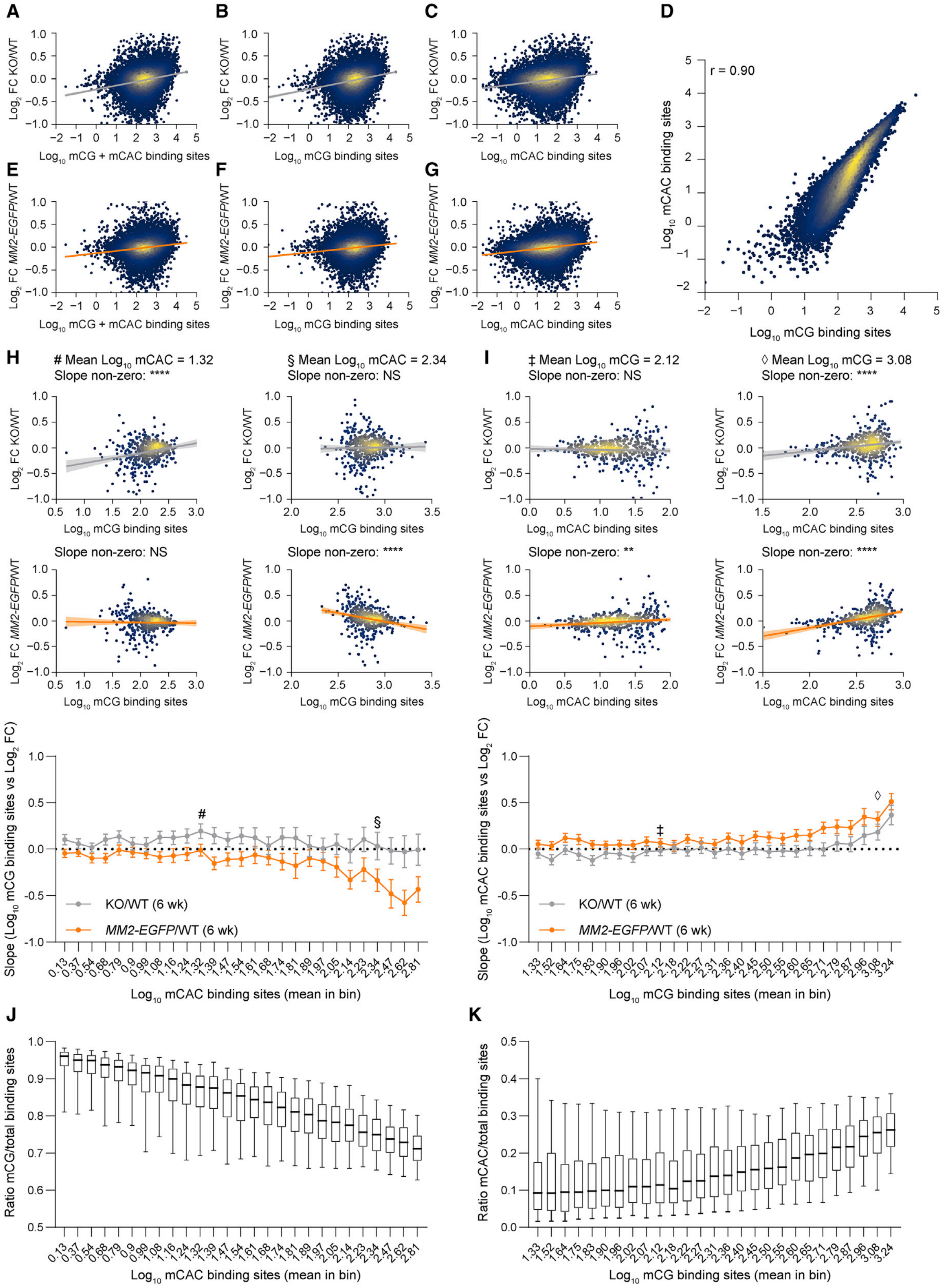

(legend on next page)

explore the possibility that the shared genes could lead us to candidates whose altered expression contributes strongly to the RTT phenotype, we performed Disease Ontology analysis on each of the three groups: shared (purple), KO only (gray), and *MM2-EGFP* only (orange). We found that the shared genes were enriched in genes associated with four categories of neurological disease: "pervasive developmental disorder," "autism spectrum disorder," "autistic disorder," and "developmental disorder of mental health" (Table S3). It is notable that the same analysis on genes dysregulated only in KO mice or only in *MM2-EGFP* mice gave no significant disease hits. There was a strong overlap between the four neurological disease categories, with all 20 genes associated with developmental disorder of mental health present in one or more of the other categories. The candidate genes were similarly dysregulated in both mutants in this study and an independent hypothalamus dataset (Figure 7C) (Chen et al., 2015). Many of these genes were also dysregulated in KO cortical tissue (Figure 7C) (Boxer et al., 2019), suggesting consistency between different regions of the RTT brain.

Differential gene expression could contribute to the disease state regardless of the direction of change, but 15 of 20 candidate genes were upregulated in both mutants (Figure 7C), suggesting that they are direct MeCP2 targets. Comparisons between all of the shared dysregulated genes and those affected in KO or *MM2-EGFP* only or unaffected in either mutant showed that shared genes had the highest number of methylated binding sites (mCG + mCAC) (Figures 7D and S7B), highest mCAC:total ratios (Figures 7E and S7C), and tended to be more highly upregulated (Figures 7F and S7D). Interestingly, the 20 candidate genes have exceptionally high levels of methylation, proportion of mCAC sites, and transcriptional upregulation (Figures 7D–7F and S7B–S7D). These genes represent strong candidates for future investigation as their dysregulation may contribute disproportionately to the neurological features of RTT.

DISCUSSION

This study investigated the biological significance of the dual-binding specificity of MeCP2. MBD functionality is essential for the role of MeCP2 protein, but as RTT-causing missense mutations in this domain disrupt both mCG and mCAC binding (Brown et al., 2016), the importance of its ability to target mCAC sites was previously unknown. Our results show that mice dependent on a derivative of MeCP2 (MM2) that can bind mCG but not mCAC develop severe RTT-like symptoms. We note that MM2 protein is slightly more abundant (1.16-fold) and has altered mCG binding dynamics, compared to native MeCP2. However, these differences are quite modest and are therefore likely to have minimal phenotypic consequences. For comparison, 2-fold overexpression of WT MeCP2 results in neurological defects that are considerably milder than RTT models (Collins et al., 2004). Despite the reduced affinity for mCG in the *in vitro* and cell-based assays (Figures 2D and 2F), MM2-EGFP occupies mCG sites in the brain at least as highly as WT protein (Figures 3E and 3F). We therefore conclude that the ability of MeCP2 to target mCAC is essential to avoid the neurological problems associated with RTT and cannot be compensated for by binding to mCG alone.

The deposition and interpretation of non-CG methylation is essential for neurological function

Previous studies have investigated whether loss of the mCAC writer DNMT3A has similar consequences to loss of the reader MeCP2 (Gabel et al., 2015; Lavery et al., 2020; Stroud et al., 2017). Although DNMT3A preferentially methylates CG over CH (Ramsahoye et al., 2000), its expression in postnatal neurons, particularly during the first 3 weeks of life (Feng et al., 2005), mainly leads to mCH (predominantly mCAC) deposition, as the majority of CG dinucleotides have already been methylated in early development (Lavery et al., 2020; Stroud et al., 2017). The difference in timing means that writing of these marks can be uncoupled by Cre-mediated deletion *of Dnmt3a* after the early embryonic deposition of mCG but before the postnatal deposition of mCH. Accordingly, the deletion of *Dnmt3a* in the central nervous system (CNS) between embryonic day (E) 7.5 and E15.5 using *Nestin-Cre* abolishes mCH in the brain with a much lower impact on mCG (Dubois et al., 2006; Gabel et al., 2015). These mice display neurological defects, including hypoactivity, hind limb clasping, and gait and motor defects, resulting in premature death at 18 weeks of age (Nguyen et al., 2007; Stroud et al., 2017). The strong overlap with the RTT phenotype is consistent with the interpretation of this mark by MeCP2. At the molecular level, loss of either DNMT3A or MeCP2 results in overlapping transcriptional changes (Gabel et al., 2015; Lavery et al., 2020). Our *MM2* mice build on this evidence by showing that specifically abolishing the ability of MeCP2 to read mCH is sufficient to cause many of the neurological defects and transcriptional changes associated with the RTT phenotype.

It has been speculated that the postnatal deposition of mCH could explain the delayed onset of the RTT phenotype (Chen et al., 2015; Lavery et al., 2020), implying that MeCP2 binding

Figure 6. MM2 represses transcription at mCG but not mCAC sites

(A–C and E–G) Correlations between the total number of MeCP2 binding sites per gene (between TSS and TTS): mCG + mCAC, mCG, and mCAC and transcriptional changes in KO/WT (A–C) and *MM2-EGFP*/WT (E–G) hypothalamus tissue at 6 weeks of age.

(D) Correlation between mCG and mCAC binding sites per gene in the hypothalamus at 6 weeks. Regression (r) = 0.90.

(E–G) Correlations between MeCP2 binding sites and transcriptional changes in *MM2-EGFP*/WT.

(H) Genes were binned by the number of mCAC binding sites to determine the effect of mCG on transcription in KO/WT and *MM2-EGFP/WT* (6 weeks). Two example bins are shown (# bin 12, mean $\log_{10}$ mCAC = 1.32; and § bin 25, mean $\log_{10}$ mCAC = 2.34). The slopes (±95% confidence interval) of all of the bins are shown below. See Table S1.

(I) Genes were binned by the number of mCG binding sites to determine the effect of mCAC on transcription. Two example bins are shown (‡ bin 11, mean $\log_{10}$ mCG = 2.12; and ‡ bin 27, mean $\log_{10}$ mCAC = 3.08). The slopes (±95% confidence interval) of all of the bins are shown below. See Table S2.

(J) The ratio of mCG/total binding sites per gene in each mCAC bin.

(K) The ratio of mCAC/total binding sites per gene in each mCG bin. Whiskers show 5th–95th percentiles.

CellPress
OPEN ACCESS

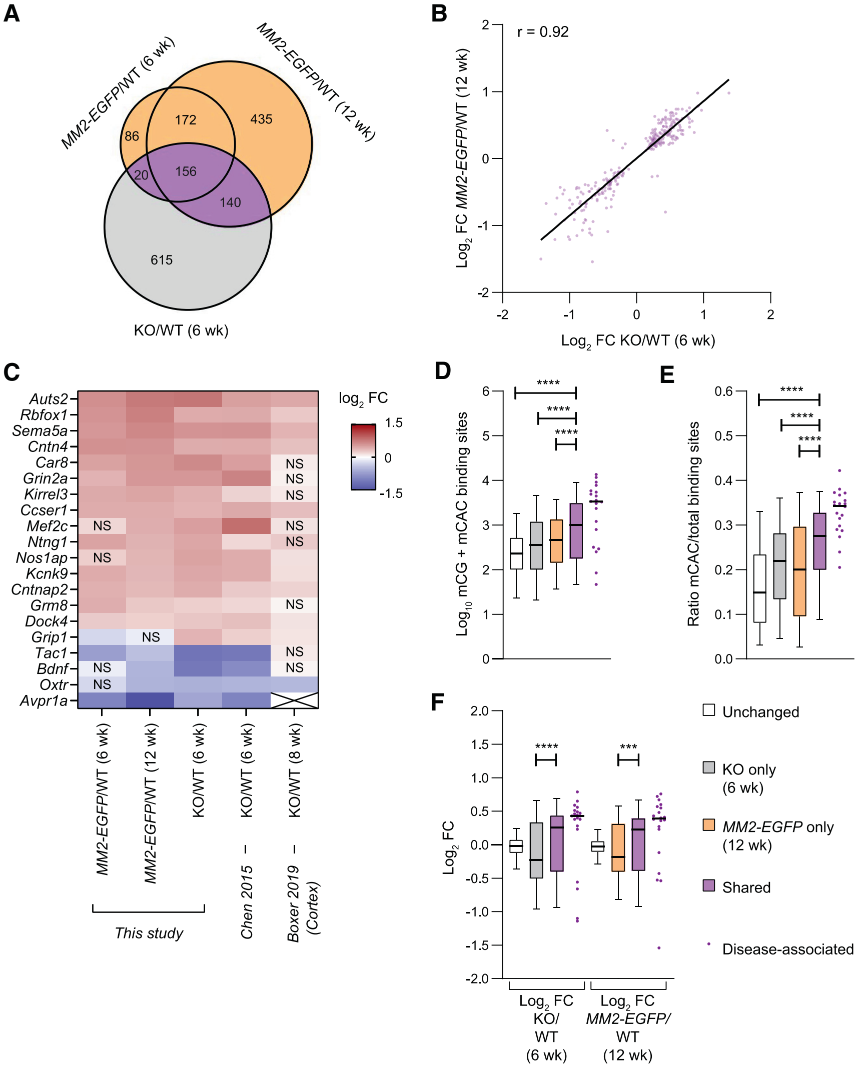

Figure 7. Shared dysregulated genes are linked to neurological disease

(A) Venn diagram showing the overlap between significantly dysregulated genes in KO (6 weeks), *MM2-EGFP* (6 weeks), and *MM2-EGFP* (12 weeks), each compared to age-matched WT controls. Significance cutoff: adjusted p (padj) < 0.05.

(B) Shared genes are dysregulated in the same direction in KO/WT (6 weeks) and *MM2-EGFP*/WT (12 weeks).

(C) Heatmap showing transcriptional changes in the 20 neurological disease-associated genes. The 3 datasets in this study were compared to an independent KO/WT (6 weeks) hypothalamus dataset (Chen et al., 2015) and a KO/WT (8 weeks) cortex dataset (Boxer et al., 2019). *Avpr1a* is not expressed in cortex (X).

(D) Comparison of mCG + mCAC binding sites between unchanged genes, and dysregulated genes in KO only, *MM2-EGFP* only, both mutants and disease-associated genes.

(E) Ratios of mCAC to total binding sites in these gene categories.

(F) Expression changes in KO/WT and *MM2-EGFP*/*WT* in these gene categories. Black bars show median values; whiskers show 5th–95th percentiles. Pairs of gene sets were compared using Mann-Whitney tests: ***$p < 0.001$, ****$p < 0.0001$.

to canonical mCG sites may be of lesser importance. mCG binding is largely dispensable in early development (before symptom onset at weaning) and at all developmental stages in peripheral tissues where only mCG sites are available (Guy et al., 2001; Ross et al., 2016). In neurons, however, MeCP2 levels are much higher than any other cell type, plateauing at ~5 weeks of age at a level that allows MeCP2 to coat neuronal chromatin (Skene et al., 2010). In the absence of any evidence that MeCP2 interprets mCG and mCAC sites differently, the relative importance of these two motifs likely depends on their abundance and location. Considering first abundance, in mature neurons, the evidence suggests that mCG and mCH binding sites occur with approximately equal frequencies (Mo et al., 2015). It is therefore possible that the accumulation of mCAC simply increases the number of available MeCP2 binding sites. If so, then replacing MeCP2 with MM2, which retains the ability to confer transcriptional repression at more than half of the MeCP2 binding sites, would be equivalent to halving MeCP2 protein levels. Our findings rule out this possibility, as mice expressing MeCP2 at ~50% normal levels display only very mild behavioral phenotypes (Samaco et al., 2008), compared with the severe RTT-like symptoms seen in *MM2-EGFP* mice.

It is therefore likely that differences in the patterning of mCG and mCAC sites underlie their distinct functional significance. CG dinucleotides are globally depleted in the genome, but highly methylated from an early stage of development. This means that the number of mCGs per gene approaches the number of CG dinucleotides and is relatively uniform between cell types. In contrast, the number of CH motifs is more than an order of magnitude higher than CG, but they become sparsely methylated in neurons (predominantly at CAC) after mCG patterns have already been laid down. Importantly, the application of mCAC methylation is not uniform, but targets genes in inverse proportion to their expression level during the early postnatal period (Stroud et al., 2017). We show here that, on average, highly methylated genes acquire a greater proportion of mCAC motifs, so they are disproportionally affected by mCAC-dependant repression by MeCP2. Loss of mCAC-dependent repression results in their upregulation in both *Mecp2* null and *MM2-EGFP* mice, suggesting that their dysregulation contributes substantially to neurological defects. Patterns of mCAC methylation have also been reported to be more cell type specific as a result of differences in expression during its deposition (Mo et al., 2015; Stroud et al., 2017). Future work will address the roles of neuronal subtype-specific patterns of mCAC-dependent transcriptional modulation of highly methylated genes for maintaining brain function.

Candidate genes may be responsible for most RTT symptoms

Since the discovery that MeCP2 controls transcription, the RTT field has searched for key target genes. While some genes (e.g., *Bdnf*) have been shown to have larger effects (Chang et al., 2006), the preferred view is that the neurological condition is an aggregate consequence of subtle changes in hundreds of genes. In addition, many of these genes are downregulated, a likely consequence of smaller cell size (Lagger et al., 2017; Li et al., 2013; Yazdani et al., 2012). In this study, we focused on genes dysregulated in both mutants, given their overlapping phenotypes. This list comprises one-third of the genes altered in *Mecp2* null mice (n = 316), which tend to be highly methylated and therefore direct MeCP2 targets. Of the 20 candidates identified by Disease Ontology analysis, 15 were markedly upregulated in both mutants. Neurological disease is often linked with haploinsufficiency of these genes, raising the possibility that dosage may be critical, with their overexpression also being deleterious, as is the case for *MECP2* (Van Esch et al., 2005). Genes implicated in neurological disease typically encode proteins falling into two categories: those with specific roles in neuronal function and chromatin-associated factors. The candidates identified in this study span both classes. For example, CNTN4 is a neuronal membrane glycoprotein (Oguro-Ando et al., 2017) and AUTS2 activates transcription in association with non-canonical PRC1 (Gao et al., 2014). Time will tell whether the dysregulation of these candidates is involved in RTT pathology and whether any could be targeted therapeutically.

Concluding remarks

It is interesting to speculate on the origin of the dual-binding specificity of MeCP2. There is evidence that MeCP2 originally evolved as a mCG-specific DNA binding protein (Hendrich and Tweedie, 2003), as other members of the MBD protein family cannot bind to mCH (Guo et al., 2014; Liu et al., 2018). We suggest that during the course of evolution, MeCP2 has added to its repertoire the ability to bind mCAC due to the relative flexibility of the side chain of arginine 133, which is one of a pair of key arginines involved in DNA recognition (Ho et al., 2008; Lagger et al., 2017; Lei et al., 2019). In contrast, the equivalent arginine side chain in MBD2 is constrained by interactions with other parts of the protein (Liu et al., 2018; Scarsdale et al., 2011). According to this view, the coevolution of DNMT3A and MeCP2 has turned two somewhat peripheral, perhaps originally biologically unimportant, properties of each protein (mCAC methylation and mCAC binding, respectively) into essential contributors to brain stability and function (see Bird, 2020).

Limitations

Experiments designed to distinguish alternative explanations for our findings are presented throughout. MM2 is a hybrid protein in which the native DNA binding domain has been replaced by a related sequence that we show *in vivo* and *in vitro* binds only one of the two MeCP2 target motifs. Our hope that mCG binding by MM2 would resemble mCG binding by native MeCP2 was broadly supported by the evidence, but it remains possible that a component of the deleterious phenotype in mice is due to differences in mCG binding affinity. We explain in the first paragraph of the Discussion why any effect on the interaction with mCG is likely to be minor; hence our conclusion that failure to bind mCAC is primarily responsible for the RTT-like defects. The list of genes that we implicate in RTT relies on evidence that the phenotypes of *MM2-EGFP* and *Mecp2* null male mice have the same root cause. Despite their impressive credentials as MeCP2 target genes (high levels of DNA methylation; unusually strong upregulation in both mutants), their contributions to the RTT phenotype are so far untested and will be the subject of future work.

STAR★METHODS

Detailed methods are provided in the online version of this paper and include the following:

- KEY RESOURCES TABLE
- RESOURCE AVAILABILITY
  - Lead contact
  - Materials availability
  - Data and code availability
- EXPERIMENTAL MODEL AND SUBJECT DETAILS
  - Bacterial strains
  - Cell lines
  - Mouse lines
- METHOD DETAILS
  - Design of MM2
  - Plasmids
  - Generation of MM2-EGFP mice
  - Southern blotting
  - Protein purification
  - Bio-Layer Interferometry (BLI)
  - Electromobility shift assay (EMSA)
  - DNA pull-down assay
  - MeCP2 localization and TBL1X–mCherry recruitment assay
  - Fluorescence recovery after photobleaching (FRAP)
  - Immunohistochemistry
  - Immunoprecipitation and western blotting
  - Flow cytometry
  - ATAC-seq library preparation
  - RNA-seq library preparation
  - Phenotypic analysis
- QUANTIFICATION AND STATISTICAL ANALYSIS
  - Evolutionary conservation among MBD proteins
  - BLI quantification
  - EMSA quantification
  - Quantification of TBL1X recruitment
  - FRAP analysis
  - Quantification of western blots
  - Quantification of flow cytometry
  - Quantification of microscopy
  - Quantification of genomic cytosine methylation
  - Analysis of ATAC-seq footprinting
  - Analysis of RNA-seq datasets
  - Disease Ontology analysis
  - Statistical analysis of behavioral data

CellPress
OPEN ACCESS

SUPPLEMENTAL INFORMATION

Supplemental Information can be found online at https://doi.org/10.1016/j.molcel.2021.01.011.

ACKNOWLEDGMENTS

This work was funded by a Rett Syndrome Research Trust consortium grant, a Wellcome Centre grant (091580/Z/10/Z), a Wellcome Investigator Award (107930/Z/15/ Z), and a European Research Council grant (EC 694295 GenEpix) to A.B., a Ludwig Institute for Cancer Research grant, a Biotechnology and Biological Sciences Research Council (BBSRC) grant (BB/M001873/1), and a Conrad N. Hilton Foundation grant to S.K. R.T. was funded by a BBSRC Doctoral Training Partnership studentship. We thank the following people for assistance: A. Cook (advice on designing the chimeric protein), S. Picaud (providing purified MBD2[MBD] protein), A. McClure (animal husbandry), M. Waterfall (flow cytometry), and A. Kerr (statistics). We also thank members of the Bird, Kriaucionis, M.E. Greenberg, G. Mandel, and M.J. Justice laboratories for helpful discussions. A.B. and M.J.L. are members of the Simons Initiative for the Developing Brain at the University of Edinburgh. R.T. is currently a Sir Henry Wellcome Postdoctoral Fellow (210913/Z/18/Z).

AUTHOR CONTRIBUTIONS

Conceptualization, A.B., R.T., M.J.L., and S.K.; methodology, R.T., J.C.-W., S.A.K., and S.K.; software, K.C., S.W., and D.A.K.; formal analysis, K.C., S.W., R.T., J.C.-W., J.C.C., and S.A.K.; investigation, R.T., J.C.-W., S.A.K., J.C.C., M.V.K., J.S., M.J.L., D.D.S., and K.B.; writing – original draft, A.B. and R.T.; writing – review & editing, all of the authors; visualization, R.T., K.C., S.W., S.A.K., and J.C.C.; data curation, K.C., S.W., and D.A.K.; supervision, A.B. and S.K.; project administration, J.S. and D.D.S.; funding acquisition, A.B. and S.K.

DECLARATION OF INTERESTS

The authors declare no competing interests.

Received: July 3, 2020
Revised: November 17, 2020
Accepted: January 7, 2021
Published: February 8, 2021

REFERENCES

Amir, R.E., Van den Veyver, I.B., Wan, M., Tran, C.Q., Francke, U., and Zoghbi, H.Y. (1999). Rett syndrome is caused by mutations in X-linked MECP2, encoding methyl-CpG-binding protein 2. Nat. Genet. *23*, 185–188.

Ballestar, E., Yusufzai, T.M., and Wolffe, A.P. (2000). Effects of Rett syndrome mutations of the methyl-CpG binding domain of the transcriptional repressor MeCP2 on selectivity for association with methylated DNA. Biochemistry *39*, 7100–7106.

Bird, A. (2020). The Selfishness of Law-Abiding Genes. Trends Genet. *36*, 8–13.

Bird, A., Taggart, M., Frommer, M., Miller, O.J., and Macleod, D. (1985). A fraction of the mouse genome that is derived from islands of nonmethylated, CpG-rich DNA. Cell *40*, 91–99.

Bolger, A.M., Lohse, M., and Usadel, B. (2014). Trimmomatic: a flexible trimmer for Illumina sequence data. Bioinformatics *30*, 2114–2120.

Boxer, L.D., Renthal, W., Greben, A.W., Griffith, E.C., Bonev, B., Greenberg, M.E., Boxer, L.D., Renthal, W., Greben, A.W., Whitwam, T., et al. (2019). MeCP2 Represses the Rate of Transcriptional Initiation of Highly Methylated Long Genes Article MeCP2 Represses the Rate of Transcriptional Initiation of Highly Methylated Long Genes. Mol. Cell *77*, 1–16.

Brown, K., Selfridge, J., Lagger, S., Connelly, J., De Sousa, D., Kerr, A., Webb, S., Guy, J., Merusi, C., Koerner, M.V., and Bird, A. (2016). The molecular basis of variable phenotypic severity among common missense mutations causing Rett syndrome. Hum. Mol. Genet. *25*, 558–570.

Chahrour, M., Jung, S.Y., Shaw, C., Zhou, X., Wong, S.T.C., Qin, J., and Zoghbi, H.Y. (2008). MeCP2, a Key Contributor to Neurological Disease, Activates and Represses Transcription. Science *320* (5880), 1224–1229.

Chang, Q., Khare, G., Dani, V., Nelson, S., and Jaenisch, R. (2006). The disease progression of Mecp2 mutant mice is affected by the level of BDNF expression. Neuron *49*, 341–348.

Chen, L., Chen, K., Lavery, L.A., Baker, S.A., Shaw, C.A., Li, W., and Zoghbi, H.Y. (2015). MeCP2 binds to non-CG methylated DNA as neurons mature, influencing transcription and the timing of onset for Rett syndrome. Proc. Natl. Acad. Sci. USA *112*, 5509–5514.

Cheval, H., Guy, J., Merusi, C., De Sousa, D., Selfridge, J., and Bird, A. (2012). Postnatal inactivation reveals enhanced requirement for MeCP2 at distinct age windows. Hum. Mol. Genet. *21*, 3806–3814.

Cholewa-Waclaw, J., Shah, R., Webb, S., Chhatbar, K., Ramsahoye, B., Pusch, O., Yu, M., Greulich, P., Waclaw, B., and Bird, A.P. (2019). Quantitative modelling predicts the impact of DNA methylation on RNA polymerase II traffic. Proc. Natl. Acad. Sci. USA *116*, 14995–15000.

Collins, A.L., Levenson, J.M., Vilaythong, A.P., Richman, R., Armstrong, D.L., Noebels, J.L., David Sweatt, J., and Zoghbi, H.Y. (2004). Mild overexpression of MeCP2 causes a progressive neurological disorder in mice. Hum. Mol. Genet. *13*, 2679–2689.

Cong, L., Ran, F.A., Cox, D., Lin, S., Barretto, R., Hsu, P.D., Wu, X., Jiang, W., and Marraffini, L.A. (2013). Multiplex Genome Engineering Using CRISPR/VCas Systems. Science *339*, 819–823.

Connelly, J.C., Cholewa-Waclaw, J., Webb, S., Steccanella, V., Waclaw, B., and Bird, A. (2020). Absence of MeCP2 binding to non-methylated GT-rich sequences in vivo. Nucleic Acids Res. *48*, 3542–3552.

Cuddapah, V.A., Pillai, R.B., Shekar, K.V., Lane, J.B., Motil, K.J., Skinner, S.A., Tarquinio, D.C., Glaze, D.G., McGwin, G., Kaufmann, W.E., et al. (2014). Methyl-CpG-binding protein 2 (MECP2) mutation type is associated with disease severity in Rett syndrome. J. Med. Genet. *51*, 152–158.

Deaton, A.M., and Bird, A. (2011). CpG islands and the regulation of transcription. Genes Dev. *25*, 1010–1022.

Dobin, A., Davis, C.A., Schlesinger, F., Drenkow, J., Zaleski, C., Jha, S., Batut, P., Chaisson, M., and Gingeras, T.R. (2013). STAR: ultrafast universal RNA-seq aligner. Bioinformatics *29*, 15–21.

Dubois, N.C., Hofmann, D., Kaloulis, K., Bishop, J.M., and Trumpp, A. (2006). Nestin-Cre transgenic mouse line Nes-Cre1 mediates highly efficient Cre/loxP mediated recombination in the nervous system, kidney, and somite-derived tissues. Genesis *44*, 355–360.

Feng, J., Chang, H., Li, E., and Fan, G. (2005). Dynamic expression of de novo DNA methyltransferases Dnmt3a and Dnmt3b in the central nervous system. J. Neurosci. Res. *79*, 734–746.

Gabel, H.W., Kinde, B., Stroud, H., Gilbert, C.S., Harmin, D.A., Kastan, N.R., Hemberg, M., Ebert, D.H., and Greenberg, M.E. (2015). Disruption of DNA-methylation-dependent long gene repression in Rett syndrome. Nature *522*, 89–93.

Gao, Z., Lee, P., Stafford, J.M., von Schimmelmann, M., Schaefer, A., and Reinberg, D. (2014). An AUTS2-Polycomb complex activates gene expression in the CNS. Nature *516*, 349–354.

Ghosh, R.P., Horowitz-Scherer, R.A., Nikitina, T., Shlyakhtenko, L.S., and Woodcock, C.L. (2010). MeCP2 binds cooperatively to its substrate and competes with histone H1 for chromatin binding sites. Mol. Cell. Biol. *30*, 4656–4670.

Goffin, D., Allen, M., Zhang, L., Amorim, M., Wang, I.-T.J., Reyes, A.-R.S., Mercado-Berton, A., Ong, C., Cohen, S., Hu, L., et al. (2011). Rett syndrome mutation MeCP2 T158A disrupts DNA binding, protein stability and ERP responses. Nat. Neurosci. *15*, 274–283.

Guo, J.U., Su, Y., Shin, J.H., Shin, J., Li, H., Xie, B., Zhong, C., Hu, S., Le, T., Fan, G., et al. (2014). Distribution, recognition and regulation of non-CpG methylation in the adult mammalian brain. Nat. Neurosci. *17*, 215–222.

Guy, J., Hendrich, B., Holmes, M., Martin, J.E., and Bird, A. (2001). A mouse Mecp2-null mutation causes neurological symptoms that mimic Rett syndrome. Nat. Genet. *27*, 322–326.

Guy, J., Gan, J., Selfridge, J., Cobb, S., and Bird, A. (2007). Reversal of neurological defects in a mouse model of Rett syndrome. Science *315*, 1143–1147.

Guy, J., Alexander-Howden, B., FitzPatrick, L., DeSousa, D., Koerner, M.V., Selfridge, J., and Bird, A. (2018). A mutation-led search for novel functional domains in MeCP2. Hum. Mol. Genet. *27*, 2531–2545.

He, Y., and Ecker, J.R. (2015). Non-CG Methylation in the Human Genome. Annu. Rev. Genomics Hum. Genet. *16*, 55–77.

Hendrich, B., and Bird, A. (1998). Identification and characterization of a family of mammalian methyl-CpG binding proteins. Mol. Cell. Biol. *18*, 6538–6547.

Hendrich, B., and Tweedie, S. (2003). The methyl-CpG binding domain and the evolving role of DNA methylation in animals. Trends Genet. *19*, 269–277.

Ho, K.L., McNae, I.W., Schmiedeberg, L., Klose, R.J., Bird, A.P., and Walkinshaw, M.D. (2008). MeCP2 binding to DNA depends upon hydration at methyl-CpG. Mol. Cell *29*, 525–531.

Kinde, B., Wu, D.Y., Greenberg, M.E., and Gabel, H.W. (2016). DNA methylation in the gene body influences MeCP2-mediated gene repression. Proc. Natl. Acad. Sci. USA *113*, 15114–15119.

Klose, R.J., Sarraf, S.A., Schmiedeberg, L., McDermott, S.M., Stancheva, I., and Bird, A.P. (2005). DNA binding selectivity of MeCP2 due to a requirement for A/T sequences adjacent to methyl-CpG. Mol. Cell *19*, 667–678.

Krishnaraj, R., Ho, G., and Christodoulou, J. (2017). RettBASE: Rett syndrome database update. Hum. Mutat. *38*, 922–931.

Kruusvee, V., Lyst, M.J., Taylor, C., Tarnauskaitė, Ž., Bird, A.P., and Cook, A.G. (2017). Structure of the MeCP2-TBLR1 complex reveals a molecular basis for Rett syndrome and related disorders. Proc. Natl. Acad. Sci. USA *114*, E3243–E3250.

Kudo, S., Nomura, Y., Segawa, M., Fujita, N., Nakao, M., Schanen, C., and Tamura, M. (2003). Heterogeneity in residual function of MeCP2 carrying missense mutations in the methyl CpG binding domain. J. Med. Genet. *40*, 487–493.

Lagger, S., Connelly, J.C., Schweikert, G., Webb, S., Selfridge, J., Ramsahoye, B.H., Yu, M., He, C., Sanguinetti, G., Sowers, L.C., et al. (2017). MeCP2 recognizes cytosine methylated tri-nucleotide and di-nucleotide sequences to tune transcription in the mammalian brain. PLoS Genet. *13*, e1006793.

Lavery, L.A., Ure, K., Wan, Y.W., Luo, C., Trostle, A.J., Wang, W., Jin, H., Lopez, J., Lucero, J., Durham, M.A., et al. (2020). Losing Dnmt3a dependent methylation in inhibitory neurons impairs neural function by a mechanism impacting Rett syndrome. eLife *9*, 1–27.

Lei, M., Tempel, W., Chen, S., Liu, K., and Min, J. (2019). Plasticity at the DNA recognition site of the MeCP2 mCG-binding domain. Biochim. Biophys. Acta. Gene Regul. Mech. *1862*, 194409.

Lewis, J.D., Meehan, R.R., Henzel, W.J., Maurer-Fogy, I., Jeppesen, P., Klein, F., and Bird, A. (1992). Purification, sequence, and cellular localization of a novel chromosomal protein that binds to methylated DNA. Cell *69*, 905–914.

Li, Y., Wang, H., Muffat, J., Cheng, A.W., Orlando, D.A., Lovén, J., Kwok, S.M., Feldman, D.A., Bateup, H.S., Gao, Q., et al. (2013). Global transcriptional and translational repression in human-embryonic-stem-cell-derived Rett syndrome neurons. Cell Stem Cell *13*, 446–458.

Liao, Y., Smyth, G.K., and Shi, W. (2014). featureCounts: an efficient general purpose program for assigning sequence reads to genomic features. Bioinformatics *30*, 923–930.

Lister, R., Mukamel, E.A., Nery, J.R., Urich, M., Puddifoot, C.A., Johnson, N.D., Lucero, J., Huang, Y., Dwork, A.J., Schultz, M.D., et al. (2013). Global epigenomic reconfiguration during mammalian brain development. Science *341*, 1237905.

Liu, K., Xu, C., Lei, M., Yang, A., Loppnau, P., Hughes, T.R., and Min, J. (2018). Structural basis for the ability of MBD domains to bind methyl-CG and TG sites in DNA. J. Biol. Chem. *293*, 7344–7354.

Love, M.I., Huber, W., and Anders, S. (2014). Moderated estimation of fold change and dispersion for RNA-seq data with DESeq2. Genome Biol. *15*, 550.

Ludwig, A.K., Zhang, P., Hastert, F.D., Meyer, S., Rausch, C., Herce, H.D., Müller, U., Lehmkuhl, A., Hellmann, I., Trummer, C., et al. (2017). Binding of MBD proteins to DNA blocks Tet1 function thereby modulating transcriptional noise. Nucleic Acids Res. *45*, 2438–2457.

Lyst, M.J., Ekiert, R., Ebert, D.H., Merusi, C., Nowak, J., Selfridge, J., Guy, J., Kastan, N.R., Robinson, N.D., de Lima Alves, F., et al. (2013). Rett syndrome mutations abolish the interaction of MeCP2 with the NCoR/SMRT co-repressor. Nat. Neurosci. *16*, 898–902.

Mellén, M., Ayata, P., Dewell, S., Kriaucionis, S., and Heintz, N. (2012). MeCP2 binds to 5hmC enriched within active genes and accessible chromatin in the nervous system. Cell *151*, 1417–1430.

Mellén, M., Ayata, P., and Heintz, N. (2017). 5-Hydroxymethylcytosine accumulation in postmitotic neurons results in functional demethylation of expressed genes. Proc. Natl. Acad. Sci. USA *114*, E7812–E7821.

Mo, A., Mukamel, E.A., Davis, F.P., Luo, C., Henry, G.L., Picard, S., Urich, M.A., Nery, J.R., Sejnowski, T.J., Lister, R., et al. (2015). Epigenomic Signatures of Neuronal Diversity in the Mammalian Brain. Neuron *86*, 1369–1384.

Nan, X., Meehan, R.R., and Bird, A. (1993). Dissection of the methyl-CpG binding domain from the chromosomal protein MeCP2. Nucleic Acids Res. *21*, 4886–4892.

Nan, X., Tate, P., Li, E., and Bird, A. (1996). DNA methylation specifies chromosomal localization of MeCP2. Mol. Cell. Biol. *16*, 414–421.

Nan, X., Campoy, F.J., and Bird, A. (1997). MeCP2 is a transcriptional repressor with abundant binding sites in genomic chromatin. Cell *88*, 471–481.

Nan, X., Ng, H.H., Johnson, C.A., Laherty, C.D., Turner, B.M., Eisenman, R.N., and Bird, A. (1998). Transcriptional repression by the methyl-CpG-binding protein MeCP2 involves a histone deacetylase complex. Nature *393*, 386–389.

Nguyen, S., Meletis, K., Fu, D., Jhaveri, S., and Jaenisch, R. (2007). Ablation of de novo DNA methyltransferase Dnmt3a in the nervous system leads to neuromuscular defects and shortened lifespan. Dev. Dyn. *236*, 1663–1676.

Oguro-Ando, A., Zuko, A., Kleijer, K.T.E., and Burbach, J.P.H. (2017). A current view on contactin-4, -5, and -6: implications in neurodevelopmental disorders. Mol. Cell. Neurosci. *81*, 72–83.

Piccolo, F.M., Liu, Z., Dong, P., Hsu, C.-L., Stoyanova, E.I., Rao, A., Tjian, R., and Heintz, N. (2019). Mecp2 Nuclear Dynamics in Live Neurons Results from Low and High Affinity Chromatin Interactions. Elife *8*, e51449.

Ramsahoye, B.H., Biniszkiewicz, D., Lyko, F., Clark, V., Bird, A.P., and Jaenisch, R. (2000). Non-CpG methylation is prevalent in embryonic stem cells and may be mediated by DNA methyltransferase 3a. Proc. Natl. Acad. Sci. USA *97*, 5237–5242.

Reik, W. (2007). Stability and flexibility of epigenetic gene regulation in mammalian development. Nature *447*, 425–432.

Renthal, W., Boxer, L.D., Hrvatin, S., Li, E., Silberfeld, A., Nagy, M.A., Griffith, E.C., Vierbuchen, T., and Greenberg, M.E. (2018). Characterization of human mosaic Rett syndrome brain tissue by single-nucleus RNA sequencing. Nat. Neurosci. *21*, 1670–1679.

Ross, P.D., Guy, J., Selfridge, J., Kamal, B., Bahey, N., Tanner, K.E., Gillingwater, T.H., Jones, R.A., Loughrey, C.M., McCarroll, C.S., et al. (2016). Exclusive expression of MeCP2 in the nervous system distinguishes between brain and peripheral Rett syndrome-like phenotypes. Hum. Mol. Genet. *25*, 4389–4404.

Samaco, R.C., Fryer, J.D., Ren, J., Fyffe, S., Chao, H.T., Sun, Y., Greer, J.J., Zoghbi, H.Y., and Neul, J.L. (2008). A partial loss of function allele of methyl-CpG-binding protein 2 predicts a human neurodevelopmental syndrome. Hum. Mol. Genet. *17*, 1718–1727.

Savitsky, P., Bray, J., Cooper, C.D.O., Marsden, B.D., Mahajan, P., Burgess-Brown, N.A., and Gileadi, O. (2010). High-throughput production of human proteins for crystallization: the SGC experience. J. Struct. Biol. *172*, 3–13.

Scarsdale, J.N., Webb, H.D., Ginder, G.D., and Williams, D.C., Jr. (2011). Solution structure and dynamic analysis of chicken MBD2 methyl binding

CellPress
OPEN ACCESS

domain bound to a target-methylated DNA sequence. Nucleic Acids Res. *39*, 6741–6752.

Schmiedeberg, L., Skene, P., Deaton, A., and Bird, A. (2009). A temporal threshold for formaldehyde crosslinking and fixation. PLoS ONE *4*, e4636.

Schwenk, F., Baron, U., and Rajewsky, K. (1995). A cre-transgenic mouse strain for the ubiquitous deletion of loxP-flanked gene segments including deletion in germ cells. Nucleic Acids Res. *23*, 5080–5081.

Skene, P.J., Illingworth, R.S., Webb, S., Kerr, A.R., James, K.D., Turner, D.J., Andrews, R., and Bird, A.P. (2010). Neuronal MeCP2 is expressed at near histone-octamer levels and globally alters the chromatin state. Mol. Cell *37*, 457–468.

Sperlazza, M.J., Bilinovich, S.M., Sinanan, L.M., Javier, F.R., and Williams, D.C., Jr. (2017). Structural basis of MeCP2 distribution on non-CpG methylated and hydroxymethylated DNA. J. Mol. Biol. *429*, 1581–1594.

Stroud, H., Su, S.C., Hrvatin, S., Greben, A.W., Renthal, W., Boxer, L.D., Nagy, M.A., Hochbaum, D.R., Kinde, B., Gabel, H.W., and Greenberg, M.E. (2017). Early-Life Gene Expression in Neurons Modulates Lasting Epigenetic States. Cell *171*, 1151–1164.e16.

Tillotson, R., and Bird, A. (2019). The Molecular Basis of MeCP2 Function in the Brain. J. Mol. Biol. *432*, 1602–1623.

Tillotson, R., Selfridge, J., Koerner, M.V., Gadalla, K.K.E., Guy, J., De Sousa, D., Hector, R.D., Cobb, S.R., and Bird, A. (2017). Radically truncated MeCP2 rescues Rett syndrome-like neurological defects. Nature *550*, 398–401.

Tweedie, S., Ng, H.H., Barlow, A.L., Turner, B.M., Hendrich, B., and Bird, A. (1999). Vestiges of a DNA methylation system in Drosophila melanogaster? Nat. Genet. *23*, 389–390.

Van Esch, H., Bauters, M., Ignatius, J., Jansen, M., Raynaud, M., Hollanders, K., Lugtenberg, D., Bienvenu, T., Jensen, L.R., Gecz, J., et al. (2005). Duplication of the MECP2 region is a frequent cause of severe mental retardation and progressive neurological symptoms in males. Am. J. Hum. Genet. *77*, 442–453.

Varley, K.E., Gertz, J., Bowling, K.M., Parker, S.L., Reddy, T.E., Pauli-Behn, F., Cross, M.K., Williams, B.A., Stamatoyannopoulos, J.A., Crawford, G.E., et al. (2013). Dynamic DNA methylation across diverse human cell lines and tissues. Genome Res. *23*, 555–567.

Wendte, J.M., and Schmitz, R.J. (2018). Specifications of Targeting Heterochromatin Modifications in Plants. Mol. Plant *11*, 381–387.

Xie, W., Barr, C.L., Kim, A., Yue, F., Lee, A.Y., Eubanks, J., Dempster, E.L., and Ren, B. (2012). Base-resolution analyses of sequence and parent-of-origin dependent DNA methylation in the mouse genome. Cell *148*, 816–831.

Yazdani, M., Deogracias, R., Guy, J., Poot, R.A., Bird, A., and Barde, Y.A. (2012). Disease modeling using embryonic stem cells: MeCP2 regulates nuclear size and RNA synthesis in neurons. Stem Cells *30*, 2128–2139.

Yu, G., Wang, L.G., Han, Y., and He, Q.Y. (2012). clusterProfiler: an R package for comparing biological themes among gene clusters. OMICS *16*, 284–287.

STAR★METHODS

KEY RESOURCES TABLE

REAGENT or RESOURCE	SOURCE	IDENTIFIER
Antibodies		
Anti-MeCP2 antibody, mouse monoclonal, clone Men-8	Sigma	Cat#M7443 Lot 125M4851; RRID:AB_477235
Anti-MeCP2 antibody, mouse monoclonal, clone Mec-168	Sigma	Cat#M6818 RRID:AB_262075
Anti-GFP antibody, rabbit monoclonal, clone D5.1	New England Biolabs	Cat#2956 Lot 4; RRID: AB_1196615
Anti-NCOR1 antibody, rabbit polyclonal	Bethyl	Cat# A301-146A; RRID: AB_873086
Anti-TBL1XR1 antibody, mouse monoclonal	Santa Cruz	Cat# sc-100908; RRID: AB_1130006
Anti-HDAC3 antibody, mouse monoclonal, clone 3E11	Sigma	Cat# WH0008841M2 Lot D3011-3E11; RRID: AB_1841895
Anti-Histone H3 antibody, rabbit polyclonal	Abcam	Cat#ab1791 Lot GR242682-2; RRID: AB_302613
Anti-NeuN Antibody, clone A60	Millipore	Cat#MAB377 Lot NG1876252; RRID:AB_2298772
Anti-NeuN Antibody, clone A60, Cy3 Conjugate	Millipore	Cat#MAB377C3; RRID: AB_10918200
IRDye® 800CW Donkey anti-Mouse	LI-COR	Cat#926-32212; RRID: AB_621847
IRDye® 800CW Donkey anti-Rabbit	LI-COR	Cat#926-32213; RRID: AB_621848
IRDye® 680LT Donkey anti-Rabbit	LI-COR	Cat#926-68023; RRID:AB_10706167
Sheep Anti-Mouse IgG - HRP Conjugated	GE Healthcare	Cat#NA931; RRID: AB_772210
Donkey Anti-Rabbit IgG, HRP Conjugated	GE Healthcare	Cat#NA934; RRID: AB_772206
Bacterial and virus strains		
BL21(DE3)RIPL	Agilent	Cat#230280
BL21(DE3)-R3- pRARE2	Oxford Structural Genomic Consortium (SGC); Savitsky et al., 2010	N/A
BL21(DE3)pLysS	Promega	Cat#L119B
Chemicals, peptides, and recombinant proteins		
MeCP2 77-167, mouse	This manuscript	N/A
MBD2 139-224, human	This manuscript	N/A
MeCP2 N-term, mouse	This manuscript	N/A
MM2 N-term, derived from mouse MeCP2 and MBD2	This manuscript	N/A
Critical commercial assays		
GFP-Trap®_A beads for protein pulldown	Chromotek	Cat#gta
Puregene Core Kit A	QIAGEN	Cat#1042601
Deposited data		
RettBASE: RettSyndrome.org Variation Database	Krishnaraj et al., 2017	http://mecp2.chw.edu.au
Whole genome bisulphite sequencing, mouse hypothalamus	Lagger et al., 2017	GEO: GSE84533

(*Continued on next page*)

Continued

REAGENT or RESOURCE	SOURCE	IDENTIFIER
ATAC-seq and RNA-seq data	This manuscript	GEO: GSE152801
Independent hypothalamus RNA-seq (*Mecp2*-null versus WT)	Chen et al., 2015	GEO: GSE66870
Cortex RNA-seq (*Mecp2*-null versus WT)	Boxer et al., 2019	GEO: GSE128178
All Mendeley data	This manuscript	http://DOI.org/10.17632/4bj84x6kcy.1
**Experimental models: cell lines**		
mESC, MM2 knockin, clone 5C7	This manuscript	N/A
E14 TG2a ES cells (derived from 129/Ola male mouse embryo)	Andrew Smith at the Centre for Genome Research (now Institute for Stem Cell Research), University of Edinburgh	N/A
HeLa	Bird lab	N/A
NIH 3T3	ATCC	RRID:CVCL_0594
**Experimental models: organisms/strains**		
Mouse: *WT-EGFP*: C57BL/6J-Mecp2$^{tm3.1Bird}$	Brown et al., 2016	RRID: IMSR_JAX:014610
Mouse: *MM2-EGFP*: C57BL/6J-Mecp2$^{em7.1Bird}$	This manuscript	N/A
Mouse: *Mecp2*-null: C57BL/6J.CBA/CA-Mecp2^{tm2Bird}	Guy et al., 2001	RRID: IMSR_JAX:006849
Mouse: *CMV-Cre*; C57BL/6J- Tg(CMV-cre)1Cgn	Schwenk et al., 1995	RRID: IMSR_JAX:006054
Mouse*: T158M-EGFP*: C57BL/6J-Mecp2$^{tm4.1Bird}$	Brown et al., 2016	RRID:IMSR_JAX:026762
Mouse*: R306C-EGFP*: C57BL/6J-Mecp2$^{tm5.1Bird}$	Brown et al., 2016	RRID:IMSR_JAX:026847
Mouse*: R133C-EGFP*: C57BL/6J-Mecp2$^{tm6.1Bird}$	Brown et al., 2016	RRID:IMSR_JAX:026848
**Oligonucleotides**		
See Table S4 for primer sequences	N/A	N/A
BLI probes (methylated C is underlined): CG sense: 5′-Bio-TCTGGAACGGAATTCTTCTA-3′	Integrated DNA Technologies (IDT)	N/A
CG antisense: 5′-ATAGAAGAATTCCGTTCCAG-3′	IDT	N/A
CAC sense: 5′-Bio-TCTGGAACACAATTCTTCTA-3′	IDT	N/A
CAC antisense: 5′-ATAGAAGAATTGTGTTCCAG-3′	IDT	N/A
CAT sense: 5′-Bio-TCTGGAACATAATTCTTCTA-3′	IDT	N/A
CAT antisense: 5′-ATAGAAGAATTATGTTCCAG-3′	IDT	N/A
MBD2[MBD] EMSA probes (methylated C is underlined) CG sense: 5′-FAM-TCTGGAACGGAATTCTTCTA-3′	IDT	N/A
CG antisense: 5′-ATAGAAGAATTCCGTTCCAG-3′	IDT	N/A
CAC sense: 5′-FAM-TCTGGAACACAATTCTTCTA-3′	IDT	N/A
CAC antisense: 5′-ATAGAAGAATTGTGTTCCAG-3′	IDT	N/A
CAT sense: 5′-FAM-TCTGGAACATAATTCTTCTA-3′	IDT	N/A
CAT antisense: 5′-ATAGAAGAATTATGTTCCAG-3′	IDT	N/A
N-term EMSA probes (methylated C is underlined): CG sense: 5′-AAGCATGCAATGCCCTGGAACGGAATTC TTCTAATAAAAGATGTATCATTTTAAATGC-3′	Biomers, Germany; Lagger et al., 2017	N/A
CG antisense: 5′-GCATTTAAAATGATACATCTTTTA TTAGAAGAATTCCGTTCCAGGGCATTGCATGCTT-3′	Biomers, Germany; Lagger et al., 2017	N/A
CAC sense: 5′-AAGCATGCAATGCCCTGGAACACAATTC TTCTAATAAAAGATGTATCATTTTAAATGC-3′	Biomers, Germany; Lagger et al., 2017	N/A
CAC antisense: 5′- GCATTTAAAATGATACATCTTTTA TTAGAAGAATTGTGTTCCAGGGCATTGCATGCTT-3′	Biomers, Germany; Lagger et al., 2017	N/A

(*Continued on next page*)

Continued

REAGENT or RESOURCE	SOURCE	IDENTIFIER
Oligos for DNA pull down assay (all Cs are methylated):<br>CG probe: 5′-ACGTATATACGATTTACGTTATACGATT<br>ACGATATACGATTTA CGTTAATACGTTTACGATTATT<br>ACGAATTTACGTTTTTACGAATATACGAAATACGTTT<br>AATACGTAATTACGTATATTACGTATATACGATTTAC<br>GAATTACG-3′	PCR-generated;<br>Connelly et al., 2020	N/A
CAC probe: 5′-GCACACACGCACTTTGCACTATGC<br>ACTTGCACTATGCACTTTG CACTAATGCACTTGCA<br>CTTATTGCACACTTGCACTTTTGCACACACGCACA<br>ATGCACTTAATGCACAATTGCACACACTGCACACA<br>CGCACTTTGCACACTGCA-3′	PCR-generated;<br>This manuscript	N/A
CAT probe: 5′-GCATATATGCATTTTGCATTATGC<br>ATTTG CATTATGCATTTTGCATTAATGCATTTGC<br>ATTTATTGCATATTTGCATTTTTGCATATATGCA<br>TAATGCATTTAATGCATAATTGCATATATTGCAT<br>ATATGCATTTTGCATATTGCA-3′	PCR-generated;<br>This manuscript	N/A
**Recombinant DNA**		
pET28_MeCP2[MBD]	SK lab	N/A
pNIC28-Bsa4_MBD2[MBD]	This manuscript	N/A
MM2 fragment for cloning (PstI sites underlined): 5′-<br>CCACCATTCTGCAGAGCCAGCAGAGGCAGGCAA<br>AGCAGAA ACATCAGAAAGCTCAGGCTCTGCCCC<br>AGCAGTGCCAGAAGCCTCGGCTTCCCCCAAACA<br>GCGGCGCTCCATTATCCGTGACCGGGGACCTAT<br>GGACTGCCCGGCCCTCCCCCCCGGATGGAAGA<br>AGGAGGAAGTGATCCGAAAATCAGGGCTCAGTGC<br>TGGCAAGAGCGATGTCTACTACTTCAGTCCAAGT<br>GGTAAGAAGTTCAGAAGTAAACCTCAGCTGGCAA<br>GATACCTGGGAAATGCTGTTGACCTTAGCAGTTT<br>TGACTTCAGGACCGGCAAGATGATGCC<br>TTCCAGGAGAGAGCAGAAACCACCTAAGAAGCC<br>CAAATCTCCCAAAGCTCCAGGAACTGGCAGGGG<br>TCGGGGACGCCCCAAAGGGAGCGGCAC<br>TGGGAGACCAAAGGCAGCAGCATCAGAAGGTG<br>TTCAGGTGAAAAGGGTCCTGGAGAAGAGCCCTG<br>GGAAACTTGTTGTCAAGATGCCTTTCCAAGCATC<br>GCCTGGGGGTAAGGGTGAGGGAGGTGGGGCTA<br>CCACATCTGCCCAGGTCATGGTGATCAAACGCCC<br>TGGCAGAAAGCGAAAAGCTGAAGCTGACCCCCA<br>GGCCATTCCTAAGAAACGGGGTAGAAAGCCTGG<br>GAGTGTGGTGGCAGCTGCTGCAGCTGAGGCCA-3′	ThermoFisher Scientific<br>(GeneArt Strings)	N/A
pEGFP-N1_MeCP2(WT)	Tillotson et al., 2017	RRID: Addgene_110186
pEGFP-N1_MeCP2(R111G)	Tillotson et al., 2017	RRID: Addgene_110187
pEGFP-N1_MeCP2(R306C)	Tillotson et al., 2017	RRID: Addgene_110188
pEGFP-N1_MeCP2(MM2)	This manuscript	N/A
pEGFP-N1_MeCP2(MM2-RG)	This manuscript	N/A
pmCherry-N1–TBL1X	Lyst et al., 2013	N/A
pET30b_MeCP2[N-term]	This manuscript	N/A
pET30b_MM2[N-term]	This manuscript	N/A
pX330-U6-Chimeric_BB-CBh-hSpCas9 with gRNA<br>(GGTTGTGACCCGCCATGGAT)	Tillotson et al., 2017	N/A
**Software and algorithms**		
Jalview 2.8.2	Barton lab, University of Dundee	www.jalview.org
FRAP Macro	This paper	GitHub: https://doi.org/10.5281/Zenodo.2654602

(*Continued on next page*)

CellPress
OPEN ACCESS

Continued

REAGENT or RESOURCE	SOURCE	IDENTIFIER
FACSDIVA version 8.0.1	BD Biosciences	N/A
ANYMaze	Stoelting	N/A
Prism 8	GraphPad	N/A
LAS AF software	Leica	N/A
Zen Black	Zeiss	N/A
Fiji	ImageJ	N/A
Image Studio Lite Ver 5.2	LI-COR	N/A
LightCycler 480 SW 1.5	Roche	N/A

RESOURCE AVAILABILITY

Lead contact

Further information and requests for resources and reagents should be directed to and will be fulfilled by the Lead Contact, Adrian Bird a.bird@ed.ac.uk.

Materials availability

All unique/stable reagents generated in this study are available from the Lead Contact without restriction.

Data and code availability

The accession number for the ATAC-seq and RNA-seq datasets reported in this paper is GEO: GSE152801. The code for the analysis of fluorescence recovery after photobleaching (FRAP) data is available on GitHub: https://doi.org/10.5281/Zenodo.2654602. Original data have been deposited to Mendeley Data, https://doi.org/10.17632/4bj84x6kcy.1

EXPERIMENTAL MODEL AND SUBJECT DETAILS

Bacterial strains

BL21(DE3)RIPL competent cells (Agilent) were used to express MeCP2[MBD].

BL21(DE3)-R3-pRARE2 competent cells (Savitsky et al., 2010) were used to express MBD2[MBD].

BL21(DE3)pLysS competent cells (Promega) were used to express MeCP2[N-term] and MM2[N-term].

Cell lines

HeLa and NIH 3T3 (ATCC, RRID:CVCL_0594) cells were grown in DMEM (GIBCO) supplemented with 10% fetal bovine serum (FBS; GIBCO) and 1% penicillin– streptomycin (GIBCO). Low passage male E14 TG2a ES cells (derived from 129/Ola mice) were a gift from Andrew Smith at the Centre for Genome Research at the University of Edinburgh (now Institute for Stem Cell Research). ES cells were grown in Glasgow MEM (GIBCO) supplemented with 10% FBS (GIBCO, batch tested), 1% non-essential amino acids (GIBCO), 1% sodium pyruvate (GIBCO), 0.1% β -mercaptoethanol (GIBCO) and 1,000 units/ml LIF (ESGRO). Mouse ES cell status was confirmed regularly by production of germline chimeric mice from the cells (e.g., Tillotson et al., 2017). All cells were grown at 37°C, 5% CO_2.

Mouse lines

All mice used in this study were bred and maintained at the University of Edinburgh animal facilities under standard conditions, and procedures were carried out by staff licensed by the UK Home Office and in accordance with the Animal and Scientific Procedures Act 1986. All mice were housed in a specific-pathogen-free (SPF) facility. They were maintained on a 12-h light/dark cycle and given *ad libitum* access to food (RM1 or RM3) and water. They were housed in open top cages with wood chippings, tissue bedding and additional environmental enrichment in groups of up to ten animals. Mutant mice were caged with their wild-type littermates.

MM2-EGFP mice were generated in this study from edited ES cells (detailed method described below). The edited allele contained a floxed selection cassette, which was removed by breeding chimeras with CMV-Cre deleter mice (C57BL/6J- Tg(CMV-cre)1Cgn, RRID: IMSR_JAX:006054). This line was further backcrossed onto C57BL/6J to N4 before behavioral phenotyping and to at least N4 for biochemical analysis. Hemizygous *MM2-EGFP* males (and wild-type controls) were used in all experiments as symptoms develop earlier and progress more rapidly than heterozygous females (as with other *Mecp2* mutant mice). To produce behavioral cohorts, male pups from timed matings were pooled at weaning and randomly assigned to the two groups. The first cohort was monitored for overt RTT-like symptoms, weight and survival from four weeks of age until each animal's humane end-point (wild-type controls were monitored to one year of age). The second cohort underwent behavioral testing at 10-11 weeks of age. Tissues for biochemical analysis were harvested from animals at 6-19 weeks of age.

WT-EGFP (C57BL/6J-Mecp2$^{tm3.1Bird}$, RRID: IMSR_JAX:014610) mice (Brown et al., 2016) were used as age- and sex-matched controls in some biochemical assays. They were maintained on a C57BL/6J background.

Mecp2 null mice (C57BL/6J;CBA/CA-Mecp2^{tm2Bird}, RRID: IMSR_JAX:006849) mice (Guy et al., 2001) were used for ATAC-seq analysis to determine the footprints of wild-type MeCP2 and MM2-EGFP and for RNA-seq analysis. They were maintained on a mixed C57BL/6J;CBA/CA background, as breeding difficulties occur after backcrossing onto a C57BL/6J background. Hypothalamus tissue was harvested at 6 weeks of age.

Phenotypic data was compared to RTT models: *Mecp2 null* mice (C57BL/6J-Mecp2^{tm2Bird}, RRID: IMSR_JAX:006849), *T158M-EGFP* (C57BL/6J-Mecp2$^{tm4.1Bird}$, RRID:IMSR_JAX:026762), *R306C-EGFP* (C57BL/6J-Mecp2$^{tm5.1Bird}$, RRID:IMSR_JAX:026847) and *R133C-EGFP* (C57BL/6J-Mecp2$^{tm6.1Bird}$, RRID:IMSR_JAX:026848). This data was previously published in Brown et al. (2016).

METHOD DETAILS

Design of MM2

According to convention, all amino-acid numbers given refer to the e2 isoform. Numbers refer to homologous amino acids in human (NCBI accession P51608) and mouse (NCBI accession Q9Z2D6) protein, until residue 385 where there is a two-amino-acid insertion in the human protein. In this study, the MBD of mouse MeCP2 was defined as residues 94-164. This region was replaced by the MBD of mouse MBD2 (NCBI accession Q9Z2E1), comprising residues 153-217. Note: the region of MeCP2 replaced was shorter than the minimal fragment required for binding to methylated DNA (Nan et al., 1993). This region comprises the highly conserved sequence among MBD family members and contains all RTT-causing missense mutations in the MBD cluster, listed on RettBASE: http://mecp2.chw.edu.au/ (Krishnaraj et al., 2017). The region is flanked by proline residues (disrupting protein structure), which were selected as the junctions in MM2 (P93 and P165 of MeCP2). This protein was tagged at the C terminus by EGFP, connected by a short linker. To be consistent with the WT-EGFP mice (Brown et al., 2016), the linker sequence was CKDPPVAT (DNA sequence: TGTAAGGATCCACCGGTCGCCACC). Intron 3 (located in the MBD of MeCP2) was not inserted into MM2 as it was deemed dispensable due to poor evolutionary conservation.

Plasmids

For expression of MeCP2[MBD] in bacteria, cDNA encoding MeCP2 (residues 77-167) was cloned into pET28 using NdeI and EcoRI restriction sites. The protein sequence of this fragment is identical in human and mouse.

For expression of MBD2[MBD] in bacteria, cDNA encoding human MBD2 (residues 139-224, NCBI accession number NP_003918.1, from the Mammalian Gene Collection: BC032638.1, IMAGE: 5496721) was amplified by polymerase chain reaction (PCR) (primer sequences in Table S4) in the presence of Herculase II fusion DNA polymerase (Agilent Technologies). PCR products were purified (QIAquick PCR Purification Kit, QIAGEN UK) and further sub-cloned into a pET28-derived expression vector, pNIC28-Bsa4 using ligation-independent cloning (Savitsky et al., 2010). This vector includes sites for ligation-independent cloning and a Tobacco Etch Virus (TEV)-cleavable N-terminal His6-tag (extension MHHHHHHSSGVDLGTENLYFQ*SM-). After digestion with TEV protease, the protein retains an additional serine and methionine on the N terminus. The construct was transformed into competent Mach1 cells (Invitrogen, UK) to yield the final plasmid DNA.

For expression of MeCP2 WT and derivatives in mammalian cells, plasmids expressing EGFP-tagged e2 isoforms were used. Previously published (Tillotson et al., 2017) plasmids were used to express WT (RRID: Addgene_110186), R111G (RRID: Addgene_110187) and R306C (RRID: Addgene_110188). To clone MM2, the MBD of MBD2 and flanking MeCP2 sequences was synthesized (GeneStrings, Thermo Fisher Scientific; sequence in Key resources table) and cloned into the pEGFP-N1_MeCP2[WT] plasmid to replace the MeCP2 MBD sequence using PstI restriction sites (NEB). R169G was inserted into the MM2 vector using a QuikChange II XL Site-Directed Mutagenesis Kit (Agilent Technologies) (primer sequences in Table S4). pmCherry-N1_TBL1X (Lyst et al., 2013) was used in the recruitment assay. For expression of MeCP2[N-term] and MM2[N-term] in bacteria for protein production, residues 1-205 of MeCP2 and the equivalent fragment of MM2 were subcloned from the pEGFP-N1 vectors into pET30b (Novagen). The forward primer introduced a NdeI restriction site and the reverse primer introduced a C-terminal 6xHis tag and EcoRI site (primer sequences in Table S4). To overcome low efficiency, the fragments were blunt cloned into pCR4 using the Zero Blunt TOPO kit (Invitrogen) before cloning into the pET30b vector using NdeI and EcoRI (NEB).

For targeting the endogenous *Mecp2* locus in ES cells, a gene-targeting construct containing exons 3 and 4 of wild-type MeCP2 with a C-terminal tag (Tillotson et al., 2017) was edited by recombineering. The MBD region was first replaced by the counter-selection rpsL-neo cassette amplified by PCR to give 50 nt homology arms (primer sequences in Table S4). Positive clones were selected by kanamycin resistance. This cassette was subsequently replaced by the MBD of MBD2 flanked by MeCP2 sequences amplified from pEGFP-N1_MM2 (primer sequences in Table S4). Positive clones were selected by Streptomycin resistance and sequence verified. CRISPR– Cas9 technology was used to increase the targeting efficiency: the guide RNA sequence (GGTTGTGACCCGCCATGGAT) was cloned into pX330-U6-Chimeric_BB-CBh-hSpCas9 (a gift from F. Zhang; Addgene plasmid 42230 (Cong et al., 2013)).

All plasmids were sequence verified.

CellPress
OPEN ACCESS

Generation of MM2-EGFP mice

The *MM2-EGFP* targeting vector was linearized using NotI (NEB). 129/Ola E14 TG2a ES cells were passaged every two days for one week and 20x10^6 cells were collected in 600 μL HBS containing 15 μg linearized targeting vector and 15 μg of the CRISPR/Cas9 plasmid. The cells were electroporated (GenePulser X, Biorad) in a 0.4 cm cuvette at 240 V, 500 μF, ∞ resistance. The guide RNA targeted intron 2 of the wild-type gene (at the site of the NeoSTOP cassette in the targeting vector). G418-resistant clones with correct targeting at the *Mecp2* locus were identified by PCR and Southern blot screening (method described below). PCR screens were performed using Phusion polymerase (NEB): primer sequences in Table S4. Screening identified one positive clone (5C7), which was further verified by Sanger sequencing of the *Mecp2* locus and the top predicted intragenic off-target locus, *Dock5* (primer sequences in Table S4*)*. The cell line was also karyotyped by inducing metaphase arrest by treatment for 3 hours with 0.1 μg/ml colemid (KARYOMAX, GIBCO). Pelleted cells were resuspended in 300 μL growth media plus 5 mL 0.4% (w/v) KCl and incubated for 10 min. 100 μL fixative (3 parts methanol: 1 part acetic acid) was added and mixed gently. Cells were pellets at 300 *g*, 5 min and the pellet was resuspended in 5 mL fixative and incubated at room temperature for 20 min. Cells were pelleted again and resuspended in 500 μL fixative. They were dropped onto pre-chilled glass slides from ~30 cm and left to dry overnight before mounting with Vectashield containing DAPI (H-1200). Cells were photographed on a Zeiss AxioImager (Carl Zeiss UK, Cambridge) equipped with a Photometrics Prime sCMOS camera (Teledyne Photometrics) and chromosomes were counted manually using ImageJ (NIH).

Cells from *MM2-EGFP* clone 5C7 were injected into blastocysts (E3.5) obtained from C57BL/6J females after natural mating. As with previous studies, e.g., Guy et al. (2001), 15 ES cells were injected into each blastocyst and 12 injected blastocysts were transferred to pseudo-pregnant recipient females (F1 C57BL/6J;CBA/CA). Chimeric pups were recognized by coat color. This targeted locus contained a neomycin resistance gene followed by a transcriptional STOP cassette flanked by *loxP* sites ('floxed') in intron 2. This cassette was removed *in vivo* by crossing chimeras with homozygous females from the transgenic CMV-cre deleter strain (Tg(CMV-cre)1Cgn, RRID: IMSR_JAX:006054) on a C57BL/6J background. The CMV-cre transgene was subsequently bred out. Genotyping primers for the *Mecp2* locus and Cre transgenes are in Table S4.

Southern blotting

Genomic DNA was purified from ES cells using Puregene Core Kit A (QIAGEN) according to manufacturer's instructions. Genomic DNA was purified from snap-frozen mouse brain (harvested at 7-19 weeks of age) by homogenization in 3 mL lysis buffer (50 mM Tris HCl pH7.5, 100 mM NaCl, 5 mM EDTA). Proteinase K was added to a final concentration of 0.4 mg/ml and SDS to 1% (w/v). After overnight incubation at 55°C, samples were treated with 0.1 mg/ml RNase at 37°C for 1-2 hours and gDNA was extracted with 3 mL PCI (phenol:chloroform:isoamyl alcohol, Sigma). gDNA was precipitated from the aqueous phase with 2.5 volumes 100% ethanol and 0.1 volumes 3M NaOH, and dissolved in TE. For Southern blotting, 10 μg gDNA was digested with 3 μL enzyme (NEB) in a total volume of 40 μl, and ran on 0.8% gels. Gels were incubated in 0.25 M HCl (15 min) and neutralised with 0.4 M NaOH (45 min) before transferring onto ZetaProbe membranes (Biorad). 25 ng of a probe homologous to the 3′ homology arm (1038 pb fragment digested with SpeI and BamHI) was radioactively labeled with [α^{32}]dCTP (Perkin Elmer) using the Prime-a-Gene Labeling System (Promega) according to manufacturer's instructions. Membranes were blocked in Church buffer containing ~50 μg/ml Herring Sperm DNA (Sigma) at 65°C. After 30 min, the probe was added and incubated overnight. Membranes were then washed three times with 3xSSC/0.2% SDS, followed by up to three washes with 1xSSC/0.2% SDS. The blots were exposed for 1-5 days on Phosphorimager plates (GE Healthcare) and scanned using a Typhoon FLA 7000. BamHI, BsrGI and KpnI were used to screen ES cell clones and KpnI was used to verify deletion of the NeoSTOP cassette in *MM2-EGFP* knock-in mice.

Protein purification

The expression plasmid containing MeCP2[MBD] was transformed into competent BL21 (DE3)-RIPL cells (Life Technologies) and the MBD2[MBD] plasmid was transformed into competent BL21 (DE3)-R3-pRARE2 cells (a phage-resistant derivative of the BL21 (DE3) strain), with a pRARE plasmid encoding rare codon tRNAs (Invitrogen). Freshly grown colonies were cultured overnight in lysogeny broth (LB) supplemented with 50 mg/ml kanamycin and 34 mg/mL chloramphenicol at 37°C. One liter of pre-warmed terrific broth (TB) was inoculated with 10 mL of the overnight culture and incubated at 37°C. At an optical density at 600 nm (OD600) of 2.5, the culture was cooled to 18°C and expression was induced overnight at 18°C with 0.1 mM isopropyl-b-D-thiogalactopyranoside (IPTG). Cells were harvested by centrifugation (8700 x g, 15 min, 4°C) in a Beckman Coulter Avanti J-20 XP centrifuge, and then re-suspended in lysis buffer (50 mM HEPES pH 7.5 at 20°C, 1500 mM NaCl, 5% Glycerol, 1 mM tris(2-carboxyethyl)phosphine (TCEP) and 1:1000 (v/v) Protease Inhibitor Cocktail III (Calbiochem)). Cells were lysed using a high-pressure homogenizer (Emulsiflex C5, Avestin) at ~100 mPa equipped with a recirculating cooler (F250, Julabo). DNA was precipitated on ice for 30 min with 0.15% (v/v) PEI (Polyethyleneimine, Sigma) and the lysate was cleared by centrifugation (16,000 x g for 1 hour at 4C, JA 25.50 rotor, Beckman Coulter Avanti J-20 XP centrifuge) before being applied to a Nickel affinity column (nickel nitrilotriacetic acid (Ni-NTA) resin, QIAGEN Ltd., 5 ml, equilibrated with 20 mL lysis buffer). Protein was eluted with an imidazole step gradient (50 to 250 mM) in a 500 mM NaCl lysis buffer.

For MeCP2[MBD], the eluted protein after Ni-NTA purification was concentrated with 10 kDa MWCO Amicon® Ultra (EMD Millipore) concentrators and further purified by size exclusion chromatography on a HiPrep 16/60 Sephacryl S-200 gel filtration column (GE Healthcare) on an ÄktaPrime plus systems (GE/Amersham Biosciences). For MBD2[MBD], the eluted protein after Ni-NTA affinity purification was treated overnight at 4°C with TEV protease to remove the 6 × His tag and untagged proteins were further purified by

size exclusion chromatography on a Superdex 75 16/60 HiLoad gel filtration column (GE Healthcare Life Sciences). All eluted fractions were monitored by SDS-PAGE and concentrated in gel filtration buffer (10 mM HEPES pH 7.5, 500 mM NaCl and 5% glycerol) using Amicon® Ultra (EMD Millipore) concentrators with a 10 kDa MWCO cut-off. Proteins were aliquoted, flash frozen in liquid nitrogen and stored at −80°C until further use.

Expression plasmids containing MeCP2[N-term] and MM2[N-term] with a C-terminal 6xHis tag were transformed into BL21(DE3) pLysS competent cells and plated. Colonies were scraped into an overnight starter culture in LB with 50 μg/ml kanamycin and 17 μg/ml chloramphenicol at 37∘C. This was expanded in 500 mL and protein production was induced with 1 mM IPTG at 30∘C for 3 hours when the OD_{600} reached 0.6-0.8. Frozen bacterial cell pellets were mashed in 30 mL ice-cold lysis buffer (50 mM NaH_2PO_4, 100 mM NaCl, 10% glycerol, 30 mM imidazole, 0.1% NP40, and protease inhibitor tablet (Roche), 5.7 mM β-mercaptoethanol) and passed through a 21G needle. 750 U benzonase (Sigma) was added and samples were sonicated for 10 cycles of 30 s ON/OFF at 30% amplitude (Branson Digital Sonifier). NaCl concentration was increased to 300 mM and samples were centrifuged at 31,000 *g* (30 min, 4∘C) and the supernatant was transferred to new tubes. His-tagged proteins were purified using 0.5 mL $NiSO_4$-coated Chelating Sepharose Fast Flow beads (GE healthcare), incubated in lysates for 1 hr at 4∘C. Beads were washed three times in 12 mL lysis buffer (with 300 mM NaCl) and protein was eluted in five fractions each of 0.5 mL lysis buffer with 250 mM imidazole. Fractions were pooled and diluted with 5-10 mL HEPES buffer (20 mM HEPES, 300 mM NaCl, 1 mM EDTA). The samples subsequently purified using a HiTRap Sp Hp 1 mL Column (GE Healthcare) to select for positively charged proteins. Columns were washed with 10 mL HEPES buffer and protein was eluted in 1 mL HEPES buffer with 0.7 M NaCl (fraction 1) and then 1 mL 1 M NaCl (fraction 2). Fraction 2 was used in the EMSA experiments.

Bio-Layer Interferometry (BLI)

Single-stranded DNA probes were purchased from Integrated DNA Technologies (IDT) and annealed by heating at 95°C for 5 min and slowly cooled down at room temperature. See Key resources table for sequences.

The dissociation constant (K_D) of MeCP2[MBD] to different DNA probes was determined by using bio-layer interferometry on the Octet RED384 system (ForteBio, Pall). All assays were performed in low-binding black 96 well plates (Greiner) with 1,000 rpm orbital shaking and samples were diluted in freshly prepared and filtered BLI buffer (150mM NaCl, 10mM HEPES, pH8.0 and 0.05% Tween20).

First, streptavidin biosensors (18-5019, ForteBio, Pall) were hydrated in 200 μl of BLI buffer for 20 min at room temperature. Then, 5′-biotinylated double-stranded DNA probes (6 nM, final concentration) were loaded on the streptavidin biosensors for 600sec followed by quenching with biotin (5 μg/ml, final concentration) for 50 s. The DNA-loaded sensors were then submerged in wells containing increasing concentrations of MeCP2[MBD] (0-375nM) in the presence of poly(dI-dC) (1μg/ml, final concentration) (LightShift, Thermo) for 600 s followed by 300 s of dissociation time.

Electromobility shift assay (EMSA)

For the EMSA with MBD2[MBD], single-stranded FAM-labeled DNA probes were purchased from Integrated DNA Technologies (IDT) and annealed by heating at 95°C for 5 min and slowly cooled down at room temperature. See Key resources table for sequences. Assays were assembled in 10 μl reactions in binding buffer containing 20 mM HEPES (pH7.9), 150 mM KCl, 8% Ficoll, 1 mM EDTA and 0.5 mM DTT. The protein (2 μM final concentration) was incubated in binding buffer for 10 min at room temperature, then poly(dI-dC) (LightShift, Thermo) was added at a final concentration of 40 ng/uL and the reaction incubated for 10 min at room temperature. 5′-FAM-labeled DNA probe (10 nM final concentration) was added and incubated a further 25 min at room temperature. The reactions were run on a 4.0% acrylamide (ratio 37:1, Sigma) Tris Borate EDTA gel in 0.25X TBE, 150V for 3 hours at 4°C. The bandshifts were exposed at 473 nm on a fluorescent scanner (FujiFilm FLA-5100) and gel images were visualized with the software FujiFilm MultiGauge.

EMSAs with MeCP2[N-term] and MM2[N-term] were performed as described previously (Brown et al., 2016). Single-strand oligonucleotide probes from the mouse *Bdnf* promoter region were purchased from Biomers, Germany, annealed by heating at 100°C for 10 min and slowly cooled to room temperature. Probe sequences contained a central methylated, or unmethylated CG or CAC site (see Key resources table for sequences). 500 ng of probe was radio-labeled using T4 polynucleotide kinase (NEB) and purified (MinElute PCR Purification Kit, QIAGEN) according to manufacturer's instructions. 1 ng probe and 1 μg poly deoxyadenylic-thymidylic acid competitor DNA (Sigma-Aldrich) were added with 0, 1.5, 3.0 or 4.5 uM polypeptide in reaction buffer (5% glycerol, 0.1 mM EDTA, 10mM Tris HCl pH 7.5, 150 mM KCl, 0.1 mg/ml BSA) on ice for 20 minutes. Samples were run at 120 V for 70 min on a 10% acrylamide Tris Borate EDTA gel (0.075% APS, 0.00125% TEMED) in chilled TBE. The gels were exposed overnight and imaged using the Typhoon FLA 9500 scanner (GE Healthcare).

DNA pull-down assay

This assay performed as described previously (Connelly et al., 2020)(Piccolo et al., 2019). PCR-generated, biotin end-labeled 147 bp DNA probes (2 μg) were coupled to M280-streptavidin Dynabeads according to the manufacturer's instructions (Invitrogen). All cytosines in the probes were either non-methylated or methylated and only occurred in a single sequence context (CG, CAC or CAT), sequences in the Key resources table. Bead-DNA complexes were then co-incubated with 20 μg of rat brain nuclear protein extract (Mellén et al., 2017) for 1.5 hours at 4∘C. Following extensive washing, bead-bound proteins were eluted using Laemmli buffer

(Sigma) and resolved on a 4%–15% SDS-polyacrylamide gel (NEB). The presence of MeCP2 was assayed by western blot using MeCP2 (Sigma M6818; RRID:AB_262075) diluted 1:1000; with secondary detection employing Li-COR IRDye 800CW Donkey anti-Mouse (926-32212) diluted 1:10,000, then scanned using a LI-COR Odyssey CLx machine.

MeCP2 localization and TBL1X–mCherry recruitment assay

Analysis of MeCP2 localization and recruitment of TBL1X was performed as done previously (Lyst et al., 2013; Tillotson et al., 2017). NIH 3T3 cells were seeded on coverslips in six-well plates (25,000 cells per well) and transfected with 2 μg plasmid DNA (pEGFP-N1–MeCP2 alone or pEGFP- N1–MeCP2 and pmCherry-N1–TBL1X5) using JetPEI (PolyPlus Transfection). After 48 h, cells were fixed with 4% (w/v in PBS) paraformaldehyde (Sigma), stained with 4′, 6- diamidino-2-phenylindole (DAPI; Sigma) and then mounted using ProLong Diamond (Life Technologies). Images were acquired on a Leica SP5 laser scanning confocal and a HCX PLAPO 63x/1.4 objective with laser lines for DAPI (405nm), GFP (488nm) and mCherry (594nm) selected using LAS AF software (Leica).

Fluorescence recovery after photobleaching (FRAP)

NIH 3T3 cells were seeded on poly-L-lysine-coated coverslips in six-well plates (200,000 cells per well) and transfected with 2 μg plasmid DNA using JetPEI (PolyPlus Transfection). After 40-48 h, coverslips were used for live cell imaging at 37°C, 5% CO2. Images were acquired on a Zeiss LSM880 laser scanning confocal equipped with a GaSP detector and a Plan-Apochromat 63x/1.4 objective. A z stack was acquired at each time point comprising of six Z positions spaced 1 μm apart to account for any vertical movement of the bleach position in the cell. Images were acquired using a 488 nm laser at 0.2% power to minimize any photobleaching, except for the bleach spot (diameter 10 pixels = 1.6 μm) that was exposed to 100% laser power for 0.5 s (sufficient to produce an 80% decrease in the intensity at the bleach spot). A total of 295 post bleach images (1.02 s apart) were taken to ensure any recovery was measured to a steady state. Five images were taken before the bleach, the mean of which was used as the "preBleach" values. At least two cells were included in each frame, the first containing the bleach spot and the second as a control to correct for photobleaching during the course of the experiment.

Immunohistochemistry

Mice were culled using CO_2 at 14-15 weeks, and as soon as death could be confirmed, the rib cage was opened up to expose the heart. Animals were perfused with ~1 mL Lidocaine (1% in PBS) followed by ~25 mL 4% paraformaldehyde (w/v) in PBS (Sigma) pumped at 7 ml/min through the circulatory system. Brains were stored overnight in 4% PFA (w/v) in PBS before incubation in 30% sucrose (w/v) in PBS overnight and flash frozen in isopentane. 10 μm sections were cut on a Leica CM1900 cryostat at −18°C. Sections were washed twice in PBS, permeablised in 0.1% (v/v) Triton-100 in PBS (15 min), washed again and blocked in 1.5% goat serum. They were then probed with a Cy3-conjugated antibody against NeuN (Millipore MAB377C3; RRID: AB_10918200) overnight at 4°C. Sections were washed once in PBS, incubated in 1 μg/ml DAPI (10 min), washed twice more and mounted with coverslips using Prolong Diamond (ThermoFisher Scientific). Images of sections were acquired on a Zeiss LSM880 laser scanning confocal and a Plan Apochromat 63x/1.4 objective with laser lines for DAPI (405nm), GFP (488nm) and Cy3 (561 nm) selected using Zen Black software (Zeiss). Images show neurons in the dentate gyrus of the hippocampus.

Immunoprecipitation and western blotting

HeLa cells were transfected with pEGFP-N1–MeCP2 plasmids using JetPEI (PolyPlus Transfection) and harvested after 24–48 h. Fresh or frozen cell pellets were Dounce homogenized in 1.5 mL in NE1 (20 mM HEPES pH 7.5; 10 mM NaCl; 1mM $MgCl_2$; 0.1% Triton X-100; 10 mM β-mercaptoethanol; protease inhibitor tablet (Roche)). Snap-frozen brain hemispheres (harvested at 8-18 weeks of age) were Dounce homogenized in 2.5 mL NE1. Nuclei were collected by centrifugation at 845 *g* (5 min, 4∘C) and resuspended in NE1 (HeLa nuclei 200 μl; brain nuclei 2 ml). Nuclei were treated with 250 U/500 U Benzonase (Sigma) for 5 min at room temperature before increasing NaCl concentration to 150 mM and incubated under rotation for 20 min at 4∘C. Samples were centrifuged at 16,000 *g* (20 min, 4∘C) and the supernatant was taken as the nuclear extract. MeCP2–EGFP complexes were captured from nuclear extract using GFP-Trap_A beads (Chromotek) under rotation at 4°C for 20-30 min. Beads were washed four times in NE1 containing 150 mM NaCl and then proteins were eluted in 2x Laemmli Sample Buffer (Sigma) at 100°C, 5 min. Proteins were analyzed by western blotting using antibodies against MeCP2 (Sigma M7443; RRID:AB_477235) at a dilution of 1:2,000, GFP (NEB 2956; RRID: AB_1196615), NCOR1 (Bethyl A301-146A; RRID: AB_873086), TBL1XR1 (Santa Cruz sc-100908; RRID:AB_1130006) or HDAC3 (Sigma 3E11; RRID: AB_1841895), all at a dilution of 1:1,000. For detection on a LI-COR Odyssey CLx machine, blots were probed with LI-COR secondary antibodies: IRDye 800CW Donkey anti-Mouse (926-32212), IRDye 800CW Donkey anti-Rabbit (926-32213) or IRDye 680LT Donkey anti-Rabbit (926-68023) at a dilution of 1:10,000. For detection by ECL, blots were probed with anti-mouse-HRP (GE Healthcare, NA931) or anti-rabbit-HRP (GE Healthcare, NA934) at a dilution of 1:5,000.

Protein levels in whole-brain crude extracts were quantified using western blotting. Frozen half-brains (harvested at 9 weeks of age) were Dounce homogenized in 750 μL cold NE1 (20 mM HEPES pH7.9, 10 mM KCl, 1 mM MgCl2, 0.1% Triton X-100, 20% glycerol, 0.5 mM DTT, and protease inhibitors (Roche)) and treated with 750 U Benzonase (Sigma) for 15 min at room temperature. Next, 750 μL of 2x Laemmli Sample Buffer (Sigma) was added and samples were boiled for 3 min at 100°C, snap-frozen on dry ice, and

boiled again for 5 min. Samples were diluted 1/6 with 1x sample buffer before analysis by western blotting. Membranes were probed with an antibody against MeCP2 (Sigma M7443; RRID:AB_477235) at a dilution of 1:1,000, followed by LI-COR secondary antibodies (listed above). Histone H3 (Abcam ab1791) was used as a loading control (dilution 1:10,000).

Flow cytometry

Fresh brains were harvested from 12-week-old animals and Dounce-homogenized in 5 mL homogenization buffer (320 mM sucrose, 5 mM CaCl2, 3 mM Mg(Ac)2, 10 mM Tris HCl pH 7.8, 0.1 mM EDTA, 0.1% NP40, 0.1 mM PMSF, 14.3 mM β -mercaptoethanol, protease inhibitors (Roche)), and 5 mL 50% OptiPrep gradient centrifugation medium (50% Optiprep (Sigma D1556-250ML), 5 mM CaCl2, 3 mM Mg(Ac)2, 10 mM Tris HCl pH 7.8, 0.1 M PMSF, 14.3 mM β -mercaptoethanol) was added. This was layered on top of 10 mL 29% OptiPrep solution (v/v in H2O, diluted from 60% stock) in Ultra-Clear Beckman Coulter centrifuge tubes, and samples were centrifuged at 10,100 *g* (30 min, 4°C). Pelleted nuclei were resuspended in resuspension buffer (20% glycerol in DPBS (GIBCO) with protease inhibitors (Roche)). For flow cytometry analysis, nuclei were pelleted at 600 *g* (5 min, 4°C), washed in 1 mL PBTB (5% (w/v) BSA, 0.1% Triton X-100 in DPBS with protease inhibitors (Roche)) and then resuspended in 250 μl PBTB. To stain for NeuN, NeuN antibody (Millipore MAB377) was conjugated to Alexa Fluor 647 (APEX Antibody Labeling Kit, Invitrogen A10475), added at a dilution of 1:125 and incubated under rotation for 45 min at 4°C. Flow cytometry (BD LSRFortessa SORP using FACSDIVA version 8.0.1 software) was used to obtain the mean EGFP fluorescence for the total nuclei (n = 50,000 per sample) and the high-NeuN (neuronal) subpopulation (n > 8,000 per sample). Three biological replicates of each genotype were analyzed.

ATAC-seq library preparation

Hypothalami were dissected from *MM2-EGFP* (n = 3), *Mecp2* null (KO) (n = 3) and WT (n = 4, 3 *MM2-EGFP* littermates and 1 KO littermate) mice at 6-7 weeks of age. Freshly dissected tissues were homogenized using a hypotonic buffer (10 mM Tris-HCl pH 7.4, 10 mM NaCl, 3mM $MgCl_2$, 0.1% [v/v] Igepal CA-630). Isolated nuclei were counted, and 50,000 nuclei were resuspended in 50 μl of a transposition reaction mix containing 2.5 μl Nextera Tn5 Transposase and 2x TD Nextera reaction buffer. The mix was incubated for 30 min at 37 °C. DNA was purified by either the MinElute PCR kit (QIAGEN) or the Agencourt AMPure XP beads (Beckman Coulter) and PCR amplified with the NEBNext High Fidelity reaction mix (NEB) to generate DNA libraries. The libraries were sequenced as 75 bp paired-end reads on a HiSeq 2500 Illumina platform.

RNA-seq library preparation

Hypothalami were harvested at 6-7 weeks of age (referred to as "6 weeks") from *Mecp2* null (KO) mice (n = 4) and their WT littermate controls (n = 3) and *MM2-EGFP* mice (n = 3) and their WT littermate controls (n = 4); and at 12-15 weeks of age (referred to as "12 weeks") from *MM2-EGFP* mice (n = 4) and their WT littermate controls (n = 4). Total RNA was isolated from mouse hypothalami using Qiazol lysis reagent followed by purification with the RNeasy Mini kit (QIAGEN) according to manufacturer's protocol. Genomic DNA contamination was removed with the DNA-free kit (Ambion) and remaining DNA-free RNA was tested for purity using PCR for genomic loci. Total RNA was tested on the 2100 Bioanalyzer (Agilent Technologies) to ensure a RIN quality higher than 9, and quantified using a Nanodrop. Equal amounts of total RNA were taken forward for library preparation and ERCC RNA Spike-in control mix (Ambion) was added according to the manufacturer's guide. Ribosomal RNA was depleted using the RiboErase module (Roche) followed by library preparation using KAPA RNA HyperPrep Kit (Roche). The libraries were sequenced as 50 bp pair-end reads using a Nova-seq Illumina platform.

Phenotypic analysis

Consistent with previous studies (Brown et al., 2016; Tillotson et al., 2017), mice were backcrossed for four generations onto C57BL/6J before undergoing phenotypic characterization. Two separate cohorts, each consisting of 10 *MM2-EGFP* animals and 10 wild-type littermates, were produced. One cohort was scored weekly from 4 weeks of age (excluding week 31) until each mutant reached its humane end-point (wild-type controls until 52 weeks) using a system developed by Guy et al. (2007). Two wild-type controls were culled due to injuries, aged 38 and 45 weeks (censored on survival plot). Mice were scored in six categories: spontaneous activity, gait, hind-limb clasping, tremor, abnormal breathing and general appearance. Mice received a score between 0 and 2 for each category, where 0 = as wild-type, 1 = present, and 2 = severe. Intermediate scores of 0.5 and 1.5 were also used in all categories except hind-limb clasping (Cheval et al., 2012). The scores in each category were added together to give the aggregate symptomatic score for each animal. The mean scores ± SEM for all animals were plotted over time. Animals were also weighed during scoring sessions. Animals were culled if they lost more than 20% of their maximum body weight. Survival was graphed using Kaplan–Meier plots. Previously published (Brown et al., 2016) data for *Mecp2 null*, *T158M-EGFP, R306C-EGFP* and *R133C-EGFP* (all backcrossed onto C57BL/6J) were used for comparison. Scoring: *null* (n = 12), *T158M-EGFP* (n = 7), *R306C-EGFP* (n = 11) *R133C-EGFP* (n = 10); survival: *null* (n = 24), *T158M-EGFP* (n = 11), *R306C-EGFP* (n = 11), *R133C-EGFP* (n = 10); body weight: *null* (n = 20), *T158M-EGFP* (n = 15), *R306C-EGFP* (n = 11), *R133C-EGFP* (n = 10). Scoring data for the *MM2-EGFP*, *R306C-EGFP* and *R133C-EGFP* cohorts was plotted as heatmaps shaded according to the mean score for each category per week (aligned by age and time before death). Two R133C animals survived to one year of age so were excluded from the scoring analysis for the five weeks before death. Some animals reached their humane point > 4 days after they were last scored so were included in weeks −5 to −1 only; for 0 weeks prior to death: *MM2-EGFP* (n = 7), *R306C-EGFP* (n = 8), *R133C-EGFP* (n = 8).

The second backcrossed cohorts underwent behavioral analysis at 10-11 weeks of age, consistent with a previous study (Brown et al., 2016). Tests were performed over a two week period: elevated plus maze on day 1, open field test on day 2, hanging wire on day 3 and accelerating rotarod on days 6–9 (1 day of training followed by 3 days of trials). The elevated plus maze is a cross-shaped maze 65 cm above the floor with two open arms (20 × 8 cm), two closed arms (20 × 8 cm, with 25 cm high walls) and a central area (8 × 8 cm). It is set up in a dimly lit room with uniform lighting between each of the closed arms and each of the open arms. Animals were placed in the central area and left to explore for 15 min (during which the experimenter left the room). Mice were tracked using ANY-maze software (Stoelting) and the time in each area was determined. Mice were deemed to be in a particular area of the maze if ≥ 70% of their body was inside this region. The open field apparatus used consisted of a square arena measuring 50 by 50 cm, which was evenly lit (in a dimly lit room) and littered with fresh wood chippings. The animals were placed in the center of the area and left to explore for 20 min each (during which the experimenter left the room). Mice were tracked using ANYmaze software (Stoelting). Activity was assessed by total distance traveled. The hanging wire apparatus consists of a 1.5 mm diameter horizontal wire, 35 cm above the bench. Animals were placed on the wire with their forepaws by the experimenter, and the time taken to bring a hind paw to the wire was recorded. Animals were given a maximum of 30 s to complete this task. Animals that took longer or fell off the wire were given the maximum score of 30 s. The test was performed three times for each animal (with an inter-test interval of 30 min) and the mean of the three tests was calculated. The accelerating rotarod consisted of a 3 cm diameter rod that can rotate between 4 and 40 revolutions per minute (rpm). On the first day, the animals were accustomed to the apparatus in a short training session where they must stay on the track for 30 s at its lowest speed (4 rpm). On the subsequent three days, each animal underwent four trials (with an inter-trial interval of 75 min) where the speed is slowly increased from 4 rpm to 40 rpm over 5 min. The time taken to fall (latency) was recorded and the average time for each day was calculated.

All analysis was performed blind to genotype. Animals were randomly assigned to the two backcrossed cohorts and the order in which they were analyzed was randomized. Cohorts of this size have been used to successfully detect RTT-like symptoms in mice carrying patient mutations including the milder mutation, *R133C* (Brown et al., 2016).

QUANTIFICATION AND STATISTICAL ANALYSIS

Information on sample size, definition of center and dispersion, the statistical tests used and the resulting *P values* are included in the figure legends. All statistical analysis was performed using Prism 8 Software. Significance was defined as $p < 0.05$.

Evolutionary conservation among MBD proteins

The primary protein sequences of the methyl-CpG binding domains (MBDs) of human and mouse MeCP2, MBD1, MBD2 and MBD4 were aligned using ClustalWS on Jalview 2.8.2.

BLI quantification

The binding sensorgrams were analyzed using the Octet data analysis software 9.0 (ForteBio, Pall). Experimental data were fitted using a 2:1 model with global fitting (Rmax unlinked by sensors). All experiments were repeated on three independent protein batches and data represents the mean of high-confidence K_D (fit $R^2 > 0.9$ and narrow CI). K_D values were compared using t tests (unpaired, two-tailed).

EMSA quantification

Percentage shift of the probe in electromobility shift assays (EMSAs) were quantified using ImageJ (NIH) from three technical replicates. Binding affinities were compared using Two-way ANOVA.

Quantification of TBL1X recruitment

The number of co-transfected cells with TBL1X–mCherry recruitment to heterochromatic foci was determined for each MeCP2 construct. Three independent transfections were performed, and a total of 156, 150 and 151 cells were counted that expressed WT-EGFP, R306C –EGFP and MM2-EGFP, respectively. This analysis was performed blind. The total proportion of cells with TBL1X–mCherry recruitment by each mutant MeCP2 protein was compared with WT using Fisher's exact tests.

FRAP analysis

FRAP analysis was performed on NIH 3T3 cells transiently transfected with EGFP-tagged WT (n = 27) and MM2 (n = 28), over independent three transfections. Images were analyzed using a custom plugin (GitHub: https://doi.org/10.5281/Zenodo.2654602) written for ImageJ (NIH). Fluorescence values were normalized using the following equation:

$$\text{Normalised fluorescence} = \frac{\text{Mean Cell Control preBleach} - \text{Background}(t)}{\text{Control Cell Bleach}(t) - \text{Background}(t)} * \frac{(\text{FrapSpot}(t) - \text{Background}(t)}{\text{Mean FrapSpot preBleach} - \text{Background}(t)}$$

Half-lives were calculated for each cell using the following equation:

$t_{1/2}$[S] = t((F'-F0)*0.5 + F0)-t(F0), where F' = fluorescence maximum at the end of the measurement (mean of the last 10 measurements) and F0 = the fluorescence minimum at t0 (first image after the bleach); logarithmic regression lines were used (calculated in Microsoft Excel, between 0 and 150 s as the curves fitted a logarithmic trend in this portion). The half-lives reported are the mean ± SEM calculated from the replicate cells. The half-lives of WT-EGFP and MM2-EGFP where compared using a Mann-Whitney test.

Quantification of western blots

Western blots were processed using Image Studio Lite version 5.2 software. Protein levels of WT-EGFP and MM2-EGFP were compared using a t test (unpaired, two-tailed).

Quantification of flow cytometry

Protein levels in neuronal nuclei were quantified by EGFP fluorescence by Flow cytometry (BD LSRFortessa SORP using FACSDIVA version 8.0.1 software) to obtain the mean EGFP fluorescence for the total nuclei (n = 50,000 per sample) and the high-NeuN (neuronal) subpopulation (n > 8,000 per sample). Three biological replicates of each genotype were analyzed. The protein levels in MM2-EGFP mice were compared with WT-EGFP controls using t tests (unpaired, two-tailed).

Quantification of microscopy

Images of the dentate gyrus of the hippocampus were processed and quantified using Fiji (ImageJ). Pericentromeric chromatin was defined as DAPI foci above a fixed brightness threshold. The sum of the area of these foci was divided by the cell number to calculate the area of pericentromeric heterochromatin per cell. Total number of cells per genotype: *WT-EGFP* n = 967; *MM2-EGFP* n = 1129, from three biological replicates of each (*WT-EGFP1* n = 386; *WT-EGFP2* n = 318; *WT-EGFP3* n = 263; *MM2-EGFP1* n = 533; *MM2-EGFP2* n = 359; *MM2-EGFP3* n = 237). Graphs show mean ± SEM of the three biological replicates and genotypes were compared using t tests (unpaired, two-tailed).

Quantification of genomic cytosine methylation

To calculate the percentage methylation in the Whole genome bisulphite sequencing data (GEO: GSE84533) (Lagger et al., 2017), a threshold of ≥ 5-fold coverage was used. The proportion of methylation for each site was determined based on the reads covering the site for each motif, and the sum of these values was divided by the number of times that motif occurred. To determine the number of sites with different levels of methylation (100%, 75%–99%, 50%–74%, 25%–49%, 1%–24% and 0%), a threshold of ≥ 5-fold coverage was used. To calculate the approximate number of sites with different levels of methylation in the whole genome, these values were multiplied by total number of motifs in the genome/number of motifs with ≥ 5-fold coverage.

Analysis of ATAC-seq footprinting

Nextera adaptor sequence removal was performed on 75 bp paired-end reads using Trimmomatic version 0.32 with the following parameters: ILLUMINACLIP:nextera.fa:2:40:15 MINLEN:25. Surviving reads were then mapped to the mouse mm9 reference genome with bwa mem version 0.7.5a-r405 using the -M parameter. Alignments were filtered based on mapping quality (< 20) so that only uniquely mapped reads were taken forward, furthermore, reads aligning to mitochondrial DNA or Encode mm10 blacklisted regions were removed from further analysis. Read alignments were converted into normalized coverage files for visualization and downstream quantification using deepTools bamCoverage version 3.1.3 with the following parameters: -bs 1–normalizeUsing BPM–skipNAs -e. The positions of Tn5 transposase cutsites were inferred from the 5′ end of aligned sequencing reads and converted into bigWig format for further analysis.

Whole genome bisulphite sequencing data (GEO: GSE84533) (Lagger et al., 2017) was employed to visualize a footprint of MeCP2 binding across methylated and unmethylated cytosines in several sequence contexts. For mCG, 1 million randomly selected sites with 100% methylation (≥5-fold coverage between both strands) were used. For CG, sites with 0% methylation (≥10-fold coverage between both strands) were used (n = 935,582). For mCAC, sites with ≥ 75% methylation (≥5-fold coverage) were used (n = 95,017). To increase the number of sites used, the thresholds were lowered to ≥ 4-fold coverage and ≥ 50% methylation for other forms for mCH: mCAT n = 167,159; mCAG n = 172,250; mCAA n = 72,688; mCT n = 263,578; mCC n = 7,643. For all unmethylated CH motifs, 1 million randomly selected sites with 0% methylation (≥10-fold coverage) were used. The distribution of ATAC-seq cut sites around methylated and unmethylated cytosines was computed in R using the seqPlots package. The region 300 bases upstream and downstream of each cytosine was divided into single base bins and for each bin the total number of cut sites was summed across all cytosines. These values were plotted to show the number of cut-sites in the 600 bases surrounding cytosines of different methylation context. The values were normalized to the flanking regions by calculating the ratio of each bin to the mean of all bins within the outer 100 bases of the plot. For each sample the mean values of each bin were computed across all biological replicates before plotting. To determine footprints of MeCP2-dependent or MM2-EGFP-dependent chromatin inaccessibility, ATAC-seq profiles over each motif were divided by the equivalent KO profile. Graphs show ln(WT/KO) or ln(*MM2-EGFP*/KO). To directly compare occupancy of mCG sites between WT and MM2-EGFP proteins, graphs show ln(*MM2-EGFP*/WT).

Analysis of RNA-seq datasets

Raw sequencing reads were quality and adaptor trimmed using Trimmomatic v0.33 (Bolger et al., 2014). Quality trimmed reads were aligned to mm10 genome assembly and M19 transcriptome assembly using STAR v2.4.2a (Dobin et al., 2013) and read counts spanning exons were calculated using featureCounts v1.6.4 (Liao et al., 2014). Differential gene expression analysis was performed using DESeq2 v1.24.0 (Love et al., 2014) and significance threshold for a gene was set at p-adjusted value < 0.05.

Whole genome bisulphite sequencing data (GEO: GSE84533) (Lagger et al., 2017) was used to determine the number of mCG and mCAC binding sites per gene body (between the transcriptional start site and transcriptional termination site). The proportion of DNA methylation at a given site *i* corresponds to the ratio of methylated C basecalls (*mC*) for that site to the count of all reads mapping to that site ($m^i = mC / C$). The total number of methylated sites for CG and CAC motifs across a gene body was calculated by summing the respective m^i. Since a single molecule of an MBD protein binds a symmetrically methylated mCG/mCG site, the number of mCG sites was divided by two. Ordinary least-squares (OLS) linear regression analysis was used to compare the number of mCG versus mCAC binding sites per gene. Comparisons of gene expression changes to the number of methylated binding sites used all genes that were detected in all datasets (n = 14,391). Gene expression changes were determined by comparing the following pairs of datasets: KO/WT (littermate controls, at 6 weeks on C57BL/6J;CBA/CA), *MM2-EGFP*/WT (littermate controls, at 6 weeks on C57BL/6J), *MM2-EGFP*/WT (littermate controls, at 12 weeks on C57BL/6J). Genes were binned by the number of mCG or mCAC binding sites, each bin contained 500 genes (the first and last bins were excluded due to the high variance in the motif used for binning. Slopes of linear regression (with 95% confidence interval) were determined and plotted for each bin. For each bin, it was determined whether the slope was significantly non-zero. The ratio of mCG/total binding sites was calculated for genes in each of the mCAC bins and the ratio of mCAC/total binding sites was calculated for genes in each of the mCG bins. Total = mCG + mCAC. Table S5 (Excel file) contains expression change and methylation data for the 14,391 expressed genes.

Genes significantly dysregulated in mutants compared to age-matched wild-type littermate controls were identified in each dataset (KO/WT, 6 weeks; *MM2-EGFP*/WT 6 weeks; and *MM2-EGFP*/WT, 12 weeks) using an adjusted p value (padj) of < 0.05. OLS linear regression analysis was used to compare transcriptional changes of shared genes in WT/KO (6 weeks) versus *MM2-EGFP*/WT (6 weeks) and in WT/KO (6 weeks) versus *MM2-EGFP*/WT (12 weeks). Comparisons between the shared differentially expressed genes and those dysregulated in KO or MM2-EGFP only were performed separately on the age-matched and symptom-matched datasets. This analysis compared: the total number of mCG + mCAC binding sites per gene, the ratio of mCAC/total binding sites per gene and expression changes (relative to wild-type littermate controls). Shared genes were statistically compared to those unchanged in either mutant and to those affected in KO or *MM2-EGFP* only using Mann-Whitney tests. Expression changes of the neurological disease-associated genes from previously published RNA-seq datasets were added for comparison: KO/WT hypothalamus 6 weeks (GEO: GSE66870) (Chen et al., 2015) and KO/WT cortex 8 weeks (GEO: GSE128178) (Boxer et al., 2019).

Disease Ontology analysis

Dysregulated gene identifiers were converted to their *H. sapiens* orthologs and disease ontology enrichment analysis was performed using clusterProfiler (Yu et al., 2012). Over representation analysis (ORA) was used to determine whether any known disease ontologies are over-represented in the list of dysregulated genes. Hypergeometric test was used to estimate the significance of the disease ontologies and p values were adjusted to account for Type I errors using Bonferroni correction. Type I errors were also accounted for using a separate independent method called Benjamini/Hochberg correction to calculate false-discovery rates (FDR).

Statistical analysis of behavioral data

Growth curves were compared using Mixed-effects model (REML) analysis for weeks 4-48 (excluding week 31). Survival curves were compared using a Mantel–Cox test. For behavioral analysis, when all data fitted a normal distribution (open field), genotypes were compared using t tests (unpaired, two-tailed). For non-parametric datasets (elevated plus maze, hanging wire and accelerating rotarod latency to fall), genotypes were compared using Kolmogorov–Smirnov tests. Change in performance over time in the accelerating rotarod test was determined for each genotype using Friedman tests.

Molecular Cell, Volume *81*

Supplemental information

Neuronal non-CG methylation is an essential target for MeCP2 function

Rebekah Tillotson, Justyna Cholewa-Waclaw, Kashyap Chhatbar, John C. Connelly, Sophie A. Kirschner, Shaun Webb, Martha V. Koerner, Jim Selfridge, David A. Kelly, Dina De Sousa, Kyla Brown, Matthew J. Lyst, Skirmantas Kriaucionis, and Adrian Bird

SUPPLEMENTAL INFORMATION

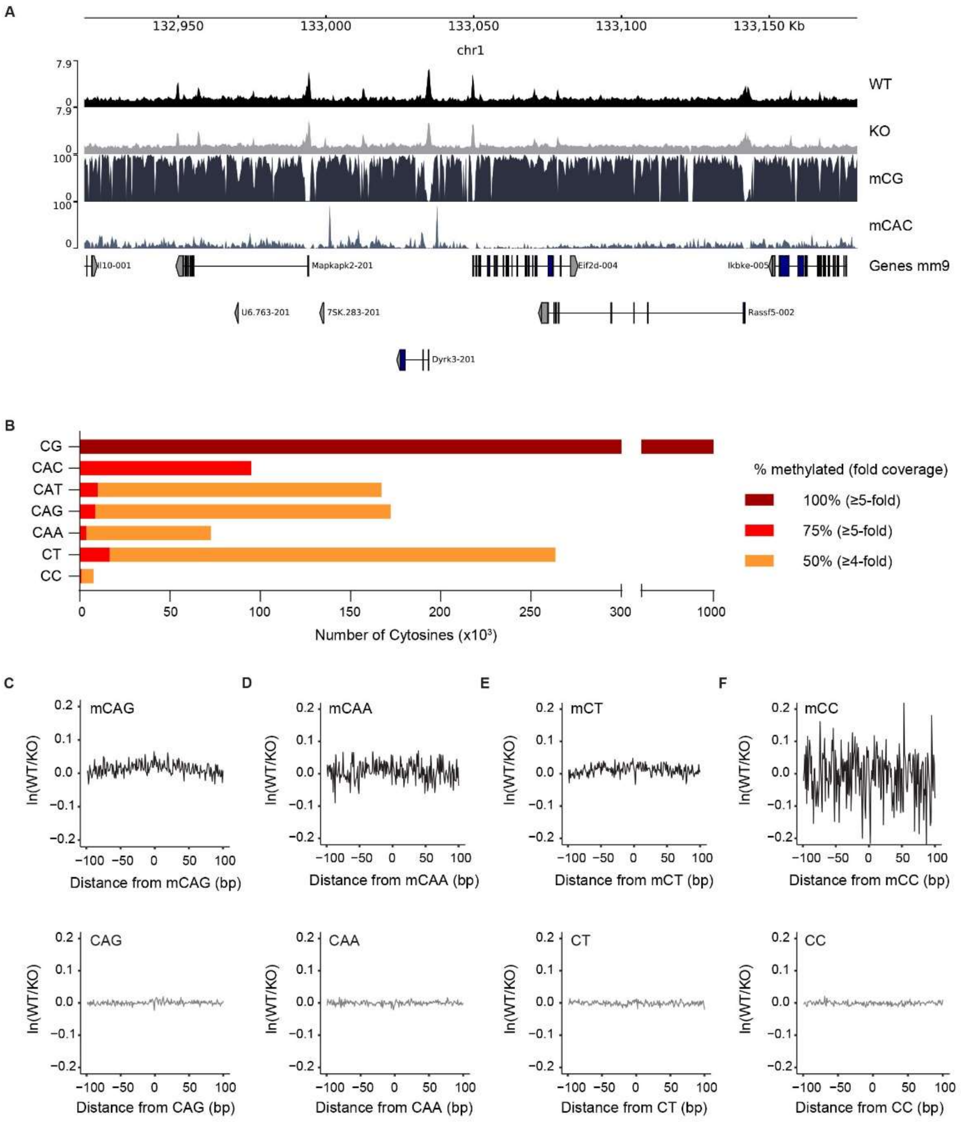

Figure S1. MeCP2 binds mCG and mCAC and to a lesser extent mCAT. ***Related to Figure 1***

A. Representative ATAC-seq tracks (cut sites) for WT (black) and KO (grey) shown alongside mCG and mCAC tracks (percentage methylation) from WGBS. Screenshot covers a region of chromosome 1 with several annotated transcriptional units (bottom). Deletion of *Mecp2* does not result in gross changes to chromatin accessibility. B. Bar graph showing the number of highly methylated sites used for ATAC-seq footprinting. mCG sites are 100% methylated on both strands, with a combined ≥5-fold coverage. mCAC sites are ≥75% methylated, with a ≥5-fold coverage. All others are ≥50% methylated, with a ≥4-fold coverage. C-F. ATAC-seq footprints over methylated (upper) and unmethylated (lower) sequences. All CH footprints used 1 million sites with 0% methylation and ≥10-fold coverage.

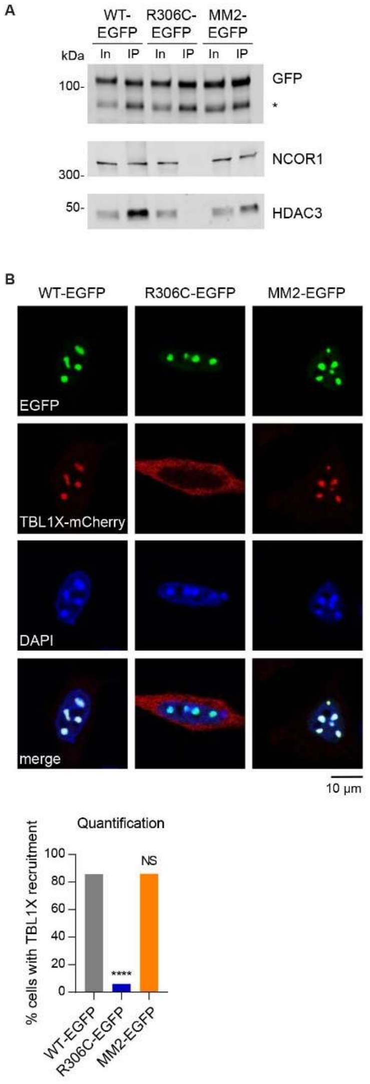

Figure S2. Chimeric protein MM2 retains the ability to bind to key interaction partners of MeCP2, the NCOR1/2 repressor complexes. ***Related to Figure 2***

A. EGFP-tagged MM2 immunoprecipitated components of the NCOR1/2 co-repressor complexes: NCOR1 and HDAC3. WT and R306C were used as positive and negative controls for binding, respectively. In = input; IP = immunoprecipitate; * band from degraded protein. B. Representative images showing recruitment of TBL1X–mCherry to heterochromatic foci by EGFP-tagged MM2 when it is co-overexpressed in NIH-3T3 cells. WT and R306C were used as positive and negative controls for TBL1X–mCherry recruitment, respectively. Scale bar, 10 μm. Quantification (below) shows the percentage of cells with focal TBL1X–mCherry localization, evaluated relative to WT-EGFP using Fisher's exact tests: R306C-EGFP, **** $P < 0.0001$; MM2-EGFP, $P > 0.99$. Total numbers of cells counted: WT-EGFP, n = 156; R306C-EGFP, n = 150; MM2-EGFP, n = 151; over three independent transfection experiments.

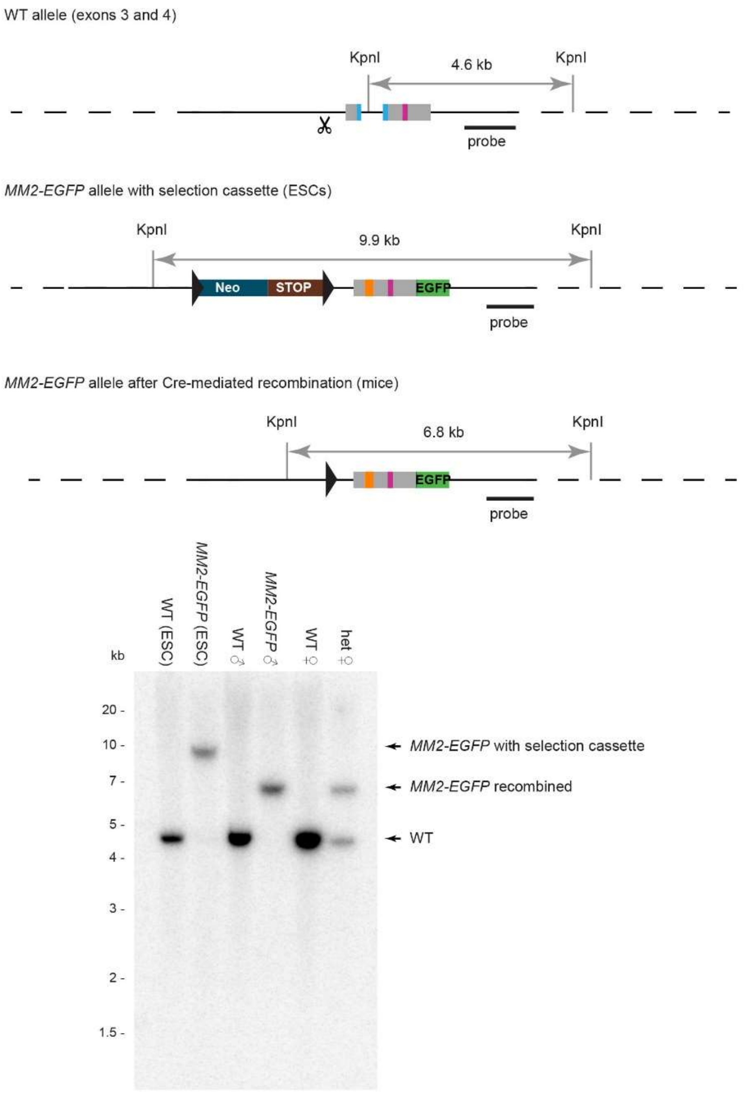

Figure S3. Generation of *MM2-EGFP* mice, Related to STAR methods: Generation of *MM2-EGFP* mice. *Related to Figure 3*

Diagrammatic representation of *MM2-EGFP* mouseline generation. The endogenous *Mecp2* allele was targeted in male ES cells. The site of Cas9 cleavage in the WT sequence is shown by the scissors symbol. The floxed selection cassette was removed *in vivo* by crossing chimeras with deleter (*CMV-cre*) transgenic mice to produce constitutively expressing *MM2-EGFP* mice. The solid black line represents the sequence encoded in the targeting vector and the dotted lines indicate the flanking regions of mouse genomic DNA. *LoxP* sites are shown as triangles. Key: *Mecp2* MBD = blue, *MM2* MBD = orange, NID = pink, intervening *Mecp2* exonic sequences = grey, EGFP = green, linker = dark green, Neomycin resistance gene = dark blue, transcriptional stop cassette = dark red. Southern blot analysis shows correct targeting of ES cells and successful cassette deletion in the *MM2-EGFP* knock-in mice.

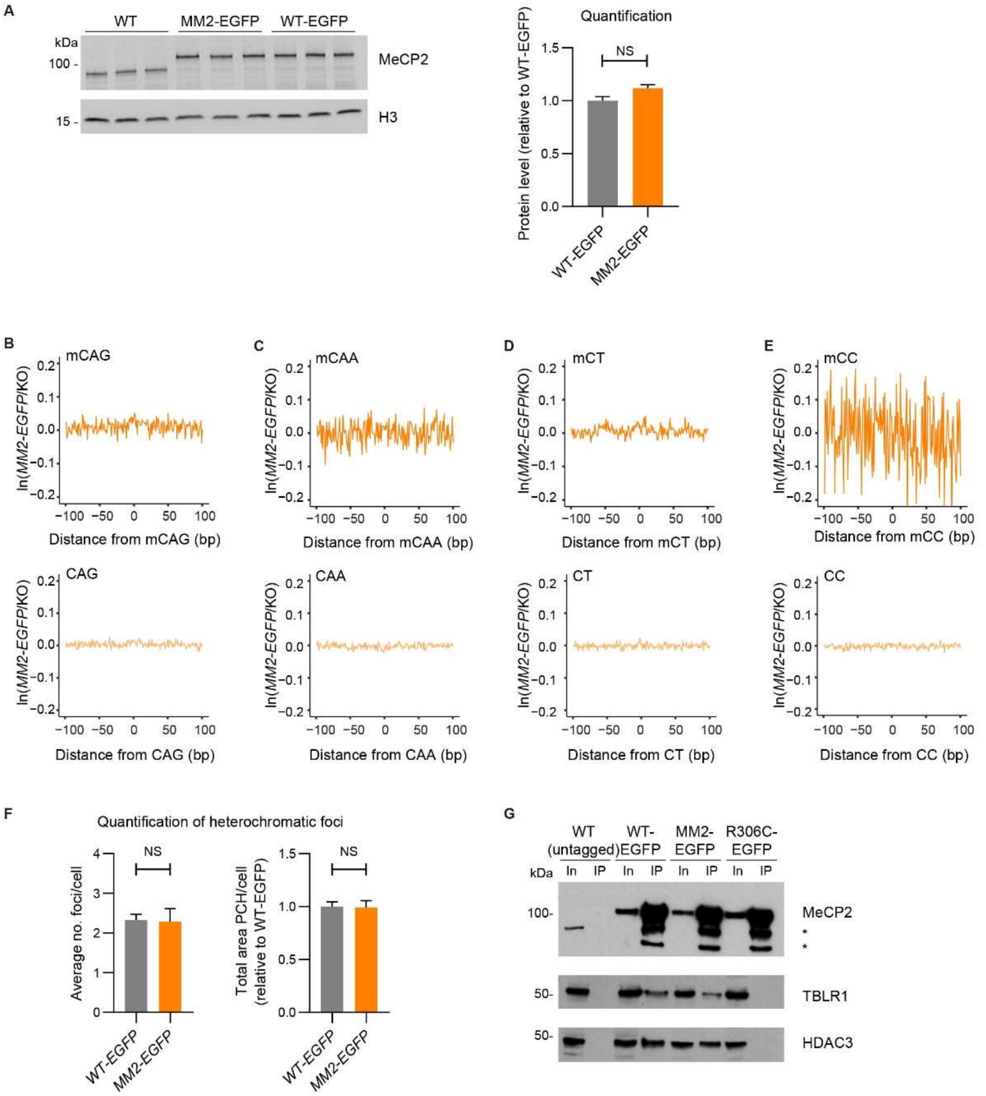

Figure S4: MM2 binds specifically to mCG *in vivo*. *Related to Figure 3*

A. Western blot analysis of whole-brain extract showing protein sizes and abundance of MM2-EGFP compared to WT and WT-EGFP controls, detected using an N-terminal MeCP2 antibody. Histone H3 was used as a loading control. Quantification (right) of WT-EGFP and MM2-EGFP protein levels, showing mean ± S.E.M. Genotypes were compared using a t-test: NS P = 0.088. B-E. ATAC-seq footprinting of MM2-EGFP at methylated (upper) and unmethylated (lower) CAG (B), CAA (C), CT (D) and CC (E) sites. Equivalent WT ATAC-seq footprinting is shown in Figure S1C-F. F. Quantification of the microscopy analysis shown in Figure 3F. The average number of foci per cell (left) and the total area of pericentromeric heterochromatin per cell (PCH) (right). Total number of cells per genotype: *WT-EGFP* n = 967; *MM2-EGFP* n = 1129, from three biological replicates of each. Graphs show mean ± SEM of biological replicates. Genotypes were compared using t-tests: number of foci NS P = 0.93; total area NS P = 0.94. G. GFP pulldowns from whole brain nuclear extract showing immunoprecipitation of members of the NCOR1/2 co-repressor complexes, TBLR1 and HDAC3, with WT-EGFP and MM2-EGFP. WT (untagged) and R306C were used as negative controls. In = input; IP = immunoprecipitate. * bands from degraded protein.

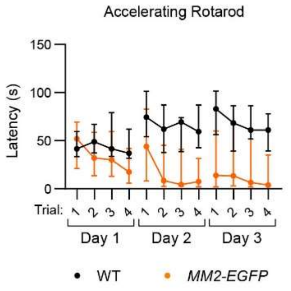

Figure S5: MM2 knock-in mice display behavioural defects associated with the RTT-like phenotype. ***Related to Figure 5***

Latency to fall from the accelerating rotarod in four trials was calculated for each of the 3 days of the experiment. Graph shows median and interquartile range between animals. WT animals did not show altered performance over the course of any of the three days: day 1 P = 0.98; day 2 P = 0.39; and day 3 P = 0.08. *MM2-EGFP* animals show fatigue over the course of day 1 * P = 0.022 and day 2 ** P = 0.004, but no change on day 3 P = 0.95. Change in performance was analysed with Friedman tests.

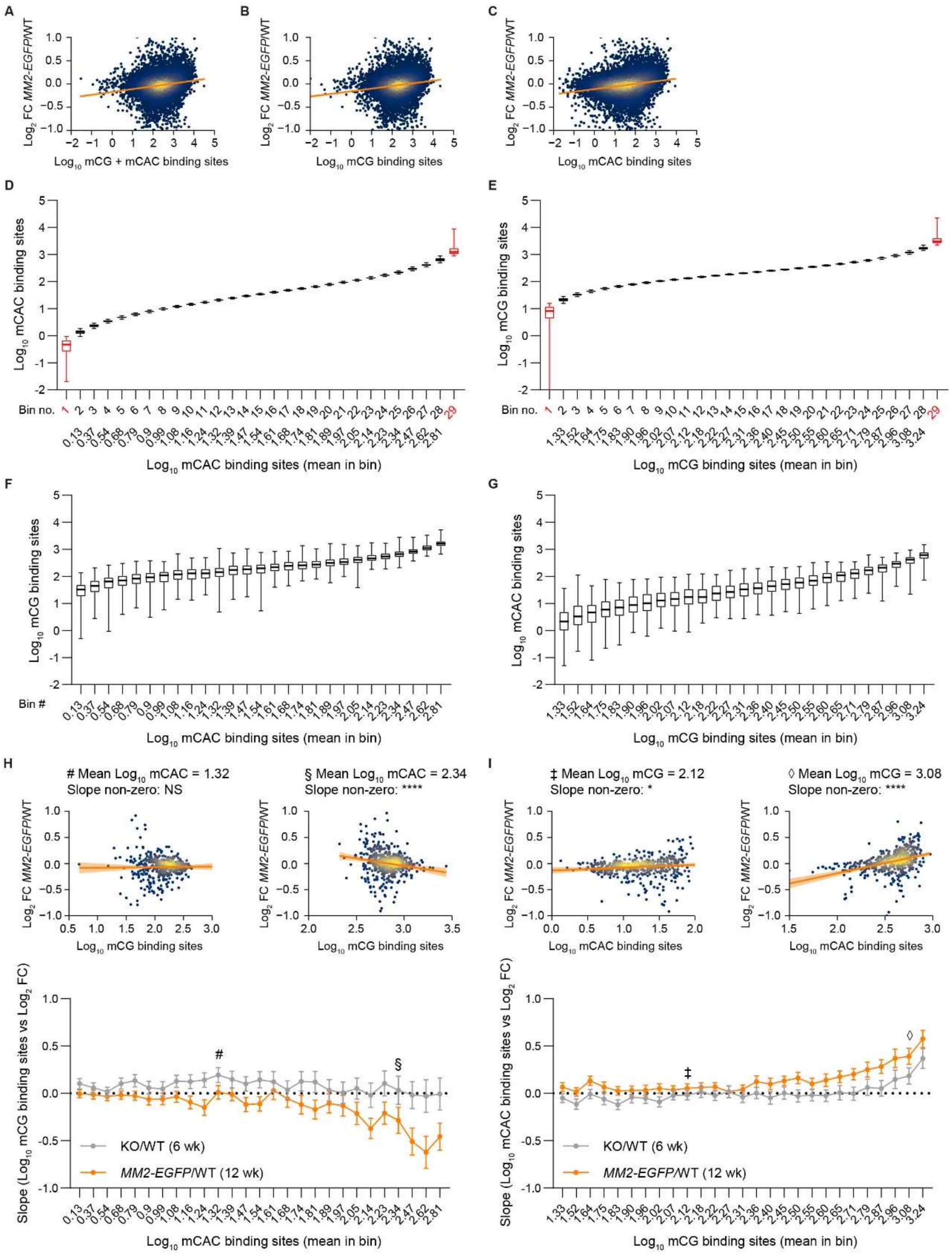

Figure S6. MM2 represses transcription at mCG but not mCAC sites. ***Related to Figure 6***

Legend on next page

Figure S6. MM2 represses transcription at mCG but not mCAC sites. ***Related to Figure 6***

A-C. Correlations between the total number of MeCP2 binding sites per gene (between TSS and TTS): mCG + mCAC (A), mCG (B) and mCAC (C) and transcriptional changes in *MM2-EGFP*/WT hypothalamus tissue at 12 weeks of age. D-G. Genes were binned by the number of mCAC binding sites to determine the effect of mCG on transcription; and by the number of mCG binding sites to determine the effect of mCAC on transcription. Whiskers show minimum-maximum. Bins no. 1 and 29 (shown in red) were excluded due to the high variance in the motif used for binning. D. Number of mCAC binding sites per gene in mCAC bins. E. Number of mCG binding sites per gene in mCG bins. F. Number of mCG binding sites per gene in mCAC bins. G. Number of mCAC sites per gene in mCG bins. H. Correlations between the number of mCG binding sites and transcriptional changes in *MM2-EGFP-EGFP/WT* (12 weeks), in mCAC bins. Two example bins are shown (# Bin 12, mean $\log_{10}$ mCAC = 1.32; and § Bin 25, mean $\log_{10}$ mCAC = 2.34). The slopes of all bins are shown below. I. Correlations between the number of mCAC binding sites and transcriptional changes in *MM2-EGFP-EGFP/WT* (12 weeks), in mCG bins. Two example bins are shown (‡ Bin 11, mean $\log_{10}$ mCG = 2.12; and ◊ Bin 27, mean $\log_{10}$ mCAC = 3.08). The slopes of all bins are shown below. Error on all slopes: 95% CI. See Tables S1-2.

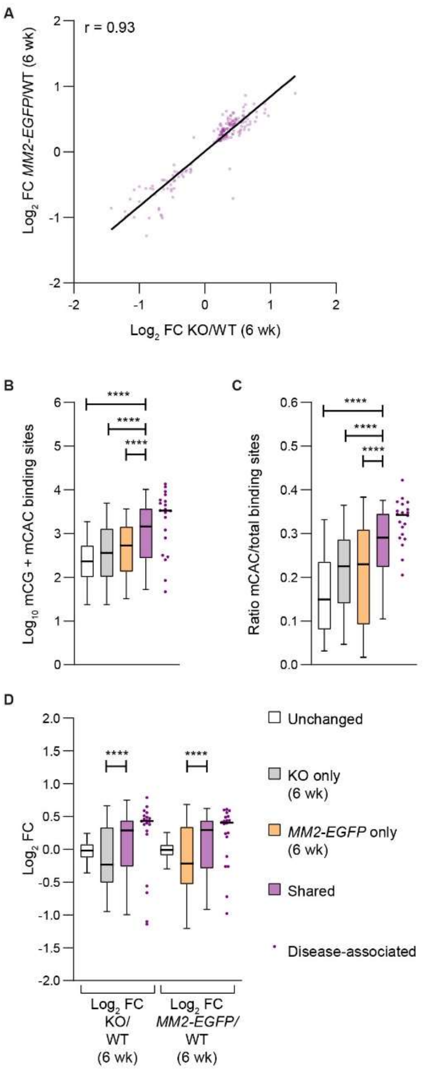

Figure S7. Shared dysregulated genes are enriched in mCAC and highly upregulated. *Related to Figure 7*

A. Shared genes are dysregulated in the same direction in KO/WT (6 weeks) and *MM2-EGFP*/WT (6 weeks). B. Shared genes have more mCG + mCAC binding sites, compared to genes unchanged in either mutant at 6 weeks and to genes dysregulated in only KO or only *MM2-EGFP* (6 weeks). C. Shared genes have higher mCAC/total binding sites ratios, compared to unchanged genes and to genes dysregulated in only KO or only *MM2-EGFP* (6 weeks). D. Shared genes are more upregulated in both mutants, compared to genes dysregulated in only KO or only *MM2-EGFP* (6 weeks). B-D. Disease-associated genes have exceptionally high mCG + mCAC levels, mCAC/total ratios and are highly upregulated. Median values are indicated by a black bar. Whiskers show 5-95 percentiles. Pairs of gene sets were compared using Mann-Whitney tests: **** $P < 0.0001$.

Table S1. Correlations between number of mCG binding sites and transcriptional changes in mCAC bins. ***Related to Figures 6 and S6***

Bin no.	Mean mCAC in bin	KO/WT (6 wk) slopes				*MM2-EGFP*/WT (6 wk) slopes				*MM2-EGFP*/WT (12 wk) slopes			
		Mean	Min	Max	Non-zero?	Mean	Min	Max	Non-zero?	Mean	Min	Max	Non-zero?
2	0.13	0.1019	0.1565	0.04727	0.0003	-0.04461	-0.000002	-0.08922	0.05	-0.00426	0.03661	-0.04513	0.8378
3	0.37	0.05908	0.1122	0.005918	0.0295	-0.03683	0.006171	-0.07982	0.093	-0.01325	0.03468	-0.06118	0.5873
4	0.54	0.0173	0.06477	-0.03018	0.4744	-0.09874	-0.04817	-0.1493	0.0001	-0.03245	0.01221	-0.0771	0.154
5	0.68	0.1036	0.1612	0.04588	0.0005	-0.09853	-0.04867	-0.1484	0.0001	-0.01876	0.02493	-0.06245	0.3993
6	0.79	0.1336	0.1981	0.06913	<0.0001	-0.0094	0.04641	-0.0652	0.7409	-0.02627	0.02125	-0.0738	0.278
7	0.9	0.05882	0.125	-0.00736	0.0814	-0.03391	0.02106	-0.08889	0.2261	-0.06336	-0.00488	-0.1218	0.0338
8	0.99	0.04805	0.1248	-0.0287	0.2193	-0.04938	0.003244	-0.102	0.0658	-0.06007	0.000066	-0.1202	0.0503
9	1.08	0.1272	0.1909	0.06347	0.0001	-0.08529	-0.02803	-0.1425	0.0036	-0.03586	0.0221	-0.09382	0.2248
10	1.16	0.1233	0.1961	0.05062	0.0009	-0.07127	0.002592	-0.1451	0.0586	-0.09496	-0.02807	-0.1618	0.0055
11	1.24	0.1405	0.2175	0.06363	0.0004	-0.05294	0.01903	-0.1249	0.149	-0.1497	-0.06818	-0.2312	0.0003
12 #	1.32	0.1944	0.2732	0.1155	<0.0001	-0.01177	0.05242	-0.07596	0.7188	0.01062	0.08139	-0.06015	0.7683
13	1.39	0.1462	0.2347	0.05766	0.0013	-0.1526	-0.08457	-0.2206	<0.0001	-0.01244	0.05329	-0.07816	0.7102
14	1.47	0.1009	0.1778	0.02407	0.0102	-0.1109	-0.04047	-0.1812	0.0021	-0.1175	-0.04409	-0.1908	0.0018
15	1.54	0.144	0.2298	0.05826	0.001	-0.1058	-0.02929	-0.1823	0.0068	-0.1118	-0.03542	-0.1882	0.0042
16	1.61	0.121	0.2295	0.01244	0.029	-0.06327	0.02889	-0.1554	0.178	0.02969	0.1231	-0.06376	0.5328
17	1.68	0.03768	-0.05625	0.1316	0.431	-0.09179	-0.01437	-0.1692	0.0202	-0.05623	0.03499	-0.1474	0.2264
18	1.74	0.127	0.2293	0.02469	0.0151	-0.1347	-0.04546	-0.2239	0.0032	-0.1194	-0.02161	-0.2172	0.0168
19	1.81	0.1207	0.2323	0.009107	0.0341	-0.1794	-0.04696	-0.3118	0.008	-0.1707	-0.07098	-0.2705	0.0008
20	1.89	0.03366	0.1459	-0.0786	0.5561	-0.09814	-0.0082	-0.1881	0.0325	-0.1061	-0.00581	-0.2063	0.0382
21	1.97	0.007044	0.1192	-0.1052	0.9019	-0.131	-0.032	-0.2301	0.0096	-0.1311	-0.02231	-0.2399	0.0183
22	2.05	0.05148	0.163	-0.06003	0.3648	-0.1941	-0.08622	-0.3019	0.0004	-0.212	-0.09465	-0.3294	0.0004
23	2.14	-0.02023	0.1098	-0.1502	0.7599	-0.3301	-0.2304	-0.4298	<0.0001	-0.3708	-0.2644	-0.4773	<0.0001
24	2.23	0.1049	0.2365	-0.02661	0.1177	-0.2197	-0.0944	-0.345	0.0006	-0.2083	-0.09173	-0.325	0.0005
25 §	2.34	0.0322	0.1802	-0.1158	0.6693	-0.3358	-0.2159	-0.4557	<0.0001	-0.2851	-0.1483	-0.422	<0.0001
26	2.47	-0.01775	0.1237	-0.1592	0.8053	-0.4808	-0.3275	-0.6341	<0.0001	-0.5098	-0.3681	-0.6515	<0.0001
27	2.62	-0.02933	0.1423	-0.2009	0.7371	-0.5756	-0.4388	-0.7125	<0.0001	-0.6225	-0.4547	-0.7903	<0.0001
28	2.81	-0.00665	0.1623	-0.1756	0.9384	-0.4337	-0.2959	-0.5715	<0.0001	-0.4575	-0.3142	-0.6008	<0.0001

Key

Positive correlation, with non-zero slope * P < 0.05, ** P < 0.01, *** P < 0.001, **** P < 0.0001.
Negative correlation, with non-zero slope P < 0.05 > 0.01; (lighter blue), P < 0.01 (darker blue).

Table S2. Correlations between number of mCAC binding sites and transcriptional changes in mCG bins. ***Related to Figures 6 and S6***

Bin no.	Mean mCG in bin	KO/WT (6 wk) slopes				*MM2-EGFP*/WT (6 wk) slopes				*MM2-EGFP*/WT (12 wk) slopes			
		Mean	Min	Max	Non-zero?	Mean	Min	Max	Non-zero?	Mean	Min	Max	Non-zero?
2	1.33	-0.04999	-0.00387	-0.0961	0.0337	0.0531	0.09544	0.01076	0.0141	0.06635	0.1136	0.01908	0.006
3	1.52	-0.1146	-0.06085	-0.1683	<0.0001	0.03477	0.07956	-0.01002	0.1278	0.01593	0.06003	-0.02817	0.4782
4	1.64	-0.00743	0.04127	-0.05613	0.7646	0.1207	0.1692	0.07229	<0.0001	0.1326	0.1817	0.08355	<0.0001
5	1.75	-0.06007	-0.00047	-0.1197	0.0482	0.1037	0.1572	0.05017	0.0002	0.06772	0.1205	0.01492	0.012
6	1.83	-0.1202	-0.06815	-0.1723	<0.0001	0.04979	0.08987	0.009717	0.015	0.0267	0.07079	-0.01739	0.2346
7	1.90	-0.04154	0.01688	-0.09995	0.163	0.04245	0.08551	-0.0006	0.0533	0.03174	0.07704	-0.01356	0.1693
8	1.96	-0.05237	-0.00068	-0.1041	0.0471	0.04713	0.09706	-0.00279	0.0642	0.03311	0.08658	-0.02036	0.2243
9	2.02	-0.08982	-0.03728	-0.1424	0.0008	0.04312	0.09031	-0.00406	0.0732	0.05127	0.1009	0.001693	0.0427
10	2.07	-0.02131	0.02566	-0.06828	0.3732	0.08413	0.1306	0.0377	0.0004	0.03447	0.08002	-0.01108	0.1377
11 ‡	2.12	-0.01814	0.03362	-0.0699	0.4914	0.06764	0.1123	0.02303	0.003	0.05444	0.1016	0.007283	0.0237
12	2.18	0.01455	0.07166	-0.04257	0.617	0.03627	0.0932	-0.02067	0.2113	0.0622	0.1114	0.01295	0.0134
13	2.22	-0.02293	0.03024	-0.0761	0.3972	0.11	0.1547	0.06522	<0.0001	0.06964	0.1124	0.0269	0.0015
14	2.27	0.01534	0.07877	-0.0481	0.635	0.07052	0.1217	0.01934	0.007	0.01736	0.07809	-0.04337	0.5746
15	2.31	-0.03987	0.01721	-0.09695	0.1706	0.05309	0.1023	0.003867	0.0346	0.04217	0.08883	-0.0045	0.0764
16	2.36	0.003028	0.06179	-0.05573	0.9194	0.122	0.1724	0.0716	<0.0001	0.124	0.1851	0.06295	<0.0001
17	2.40	-0.0467	0.01897	-0.1124	0.163	0.07351	0.1462	0.000788	0.0476	0.09874	0.1589	0.03856	0.0013
18	2.45	0.003569	0.06852	-0.06138	0.9141	0.1414	0.1922	0.0905	<0.0001	0.1328	0.181	0.08452	<0.0001
19	2.50	-0.0281	0.03647	-0.09268	0.3929	0.1268	0.1862	0.06744	<0.0001	0.1624	0.2214	0.1034	<0.0001
20	2.55	-0.01817	0.04249	-0.07884	0.5564	0.1139	0.1686	0.05923	<0.0001	0.1011	0.1602	0.04188	0.0009
21	2.60	-0.02415	0.0367	-0.085	0.4359	0.1478	0.2102	0.08536	<0.0001	0.1388	0.2053	0.07225	<0.0001
22	2.65	0.007451	0.0836	-0.06869	0.8476	0.1503	0.2089	0.09167	<0.0001	0.1748	0.2355	0.1141	<0.0001
23	2.71	-0.0032	0.06665	-0.07304	0.9284	0.2298	0.2893	0.1703	<0.0001	0.2029	0.2622	0.1435	<0.0001
24	2.79	0.0633	0.1487	-0.02207	0.1458	0.2407	0.3155	0.1659	<0.0001	0.2515	0.3294	0.1736	<0.0001
25	2.87	0.05235	0.1343	-0.02956	0.2098	0.2316	0.3053	0.1578	<0.0001	0.2825	0.3549	0.2102	<0.0001
26	2.96	0.1464	0.2437	0.04904	0.0033	0.3486	0.435	0.2623	<0.0001	0.3694	0.4665	0.2722	<0.0001
27 ◊	3.08	0.1834	0.2686	0.0982	<0.0001	0.3241	0.4011	0.2472	<0.0001	0.391	0.475	0.307	<0.0001
28	3.24	0.3674	0.471	0.2638	<0.0001	0.5107	0.598	0.4233	<0.0001	0.5743	0.6647	0.484	<0.0001

Key

Positive correlation, with non-zero slope $P < 0.05 > 0.01$; (lighter orange), $P < 0.01$ (darker orange).
Negative correlation, with non-zero slope $P < 0.05 > 0.01$; (lighter blue), $P < 0.01$ (darker blue).

Table S3: Disease Ontology analysis of shared dysregulated genes. ***Related to Figures 7 and S7***

DOID	Description	Gene ratio	BgRatio	P value	Adj. P	q value	Genes
0060040	pervasive developmental disorder	16/155	201/8007	1.5E-06	0.00056	0.0005	*MEF2C; RBFOX1; AVPR1A; SEMA5A; GRM8; AUTS2; GRIP1; DOCK4; NOS1AP; CNTNAP2; BDNF; OXTR; GRIN2A; NTNG1; TAC1; CNTN4*
0060041	autism spectrum disorder	15/155	190/8007	3.6E-06	0.00056	0.0005	*MEF2C; RBFOX1; AVPR1A; SEMA5A; GRM8; AUTS2; GRIP1; DOCK4; NOS1AP; CNTNAP2; BDNF; OXTR; GRIN2A; TAC1; CNTN4*
12849	autistic disorder	15/155	190/8007	3.6E-06	0.00056	0.0005	*MEF2C; RBFOX1; AVPR1A; SEMA5A; GRM8; AUTS2; GRIP1; DOCK4; NOS1AP; CNTNAP2; BDNF; OXTR; GRIN2A; TAC1; CNTN4*
0060037	developmental disorder of mental health	20/155	373/8007	3.1E-05	0.00361	0.0035	*MEF2C; RBFOX1; AVPR1A; SEMA5A; GRM8; AUTS2; KIRREL3; GRIP1; DOCK4; KCNK9; NOS1AP; CNTNAP2; CCSER1; CA8; BDNF; OXTR; GRIN2A; NTNG1; TAC1; CNTN4*

Key

DOID: Disease Ontology Unique Identification Number

Gene ratio: Ratio of genes which are part of the disease ontology in the query/input list (subset of the overlap of KO vs WT and MM2 vs WT which are associated with any disease term, not every gene is associated with disease ontology)

BgRatio: Ratio of genes which are part of the disease ontology in the background list (subset of all the genes that are associated with any disease ontology, not every gene is associated with disease ontology)

P-value: p-value associated with the hypergeometric test

Adj. P: Adjusted p-value after performing Benjamini-Hochberg correction

q value/FDR: False Discovery Rate after performing the Benjamini-Hochberg correction

Table S4: Primer sequences. ***Related to STAR methods***

Primer name	Sequence
Primers for cloning MBD2[MBD] into pNIC28-Bsa4	
MBD2 F:	TACTTCCAATCCATGAGGGGACCCCGGGCCAC
MBD2 R:	TATCCACCTTTACTGTCATCTCTGTTTGTTCTTCTGTAATTTAC
Primers for cloning MeCP2[N-term] and MM2[N-term] into pET30b	
N-term NdeI F:	GATATACATATGGTAGCTGGGATGTTAGGG
N-term His-tag EcoRI R:	AGCTCGAATTCTCAGTGGTGGTGGTGGTGGTGTTCTGATGCTGCTGCCT TTG
Primers for site-directed mutagenesis to insert R169G into MM2	
MM2 RG F:	CTGAGCCCTGATTTTCCGATCACTTCCTCCTTC
MM2 RG R:	GAAGGAGGAAGTGATCGGAAAATCAGGGCTCAG
Primers for amplifying fragments for recombineering MM2 targeting vector	
RN F:	AAGCCTCGGCTTCCCCCAAACAGCGGCGCTCCATTATCCGTGACCGGGG AGGCCTGGTGATGATGGCGGGATCG
RN R:	GGAGCTTTGGGAGATTTGGGCTTCTTAGGTGGTTTCTGCTCTCTCCTGG ATCAGAAGAACTCGTCAAGAAGGCG
TV F:	CCCCAGCAGTGCCAGAA
TV R:	CGACCCCTGCCAGTTCC
Primers for screening ES cell clones (5' end)	
F4:	TCACCATAACCAGCCTGCTCGC
R5:	TGCTAAAGCGCATGCTCCAGACT
R3:	ATTCGATGACCTCGAGGATCCG
Primers for screening ES cell clones (3' end)	
F1:	AGTTCTGGAATGGTGAGCAAGG
F2:	GACGTAAACGGCCACAAGTTCA
R2:	TCAAAAGCAGAGCAGGCAAAAG
Primers for sequencing predicted gRNA off-target	
Dock5 F:	AGTAAAGTAGATTTGTGGGTTCCG
Dock5 R:	GTAGAATGTGGAGATTTCCTCTAGC
MM2 genotyping primers	
P5:	TGGTAAAGACCCATGTGACCCAAG
P7:	GGCTTGCCACATGACAAGAC
Cre genotyping primers	
Cre F:	GACCGTACACCAAAATTTGCCTGC
Cre R:	TTACGTATATCCTGGCAGCGATC
